# Reaction Pathways and Redox States in α‐Selective Cobalt‐Catalyzed Hydroborations of Alkynes

**DOI:** 10.1002/anie.202009625

**Published:** 2020-10-15

**Authors:** Clemens K. Blasius, Vladislav Vasilenko, Regina Matveeva, Hubert Wadepohl, Lutz H. Gade

**Affiliations:** ^1^ Anorganisch-Chemisches Institut Universität Heidelberg Im Neuenheimer Feld 270 69120 Heidelberg Germany

**Keywords:** alkenyl boronates, alkynes, cobalt, pincer ligand, T-shaped complex

## Abstract

Cobalt(II) alkyl complexes supported by a monoanionic *NNN* pincer ligand are pre‐catalysts for the regioselective hydroboration of terminal alkynes, yielding the Markovnikov products with α:β‐(E) ratios of up to 97:3. A cobalt(II) hydride and a cobalt(II) vinyl complex appear to determine the main reaction pathway. In a background reaction the highly reactive hydrido species specifically converts to a coordinatively unsaturated cobalt(I) complex which was found to re‐enter the main catalytic cycle.

## Graphical abstract

Redox Dualism: A cobalt(II) hydride complex with an ancillary *NNN* pincer ligand displays twofold reactivity. It acts as catalyst and key intermediate in the α‐selective hydroboration of terminal alkynes. In the absence of alkynes, however, it provides facile access to a coordinatively unsaturated cobalt(I) species which undergoes reversible dimerization.

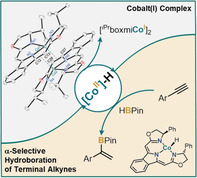

The burgeoning field of base metal catalysis has been stimulated by novel reactivity patterns and their potential in synthesis.^[1]^ Molecularly defined cobalt complexes have been established as versatile tools for a variety of catalytic transformations.^[2]^ Compared to noble metals, the propensity of cobalt compounds to engage in one‐electron redox processes has been found as a distinctive difference, such as the tendency of cobalt(II) alkyl or hydride complexes to be readily convertible into the corresponding cobalt(I) complexes.^[3]^ Cobalt(II) hydrides[^4^, ^5^]—often postulated as transient species—are thought to be key intermediates in the functionalization of C−C multiple bonds as well as hydride transfer reactions.^[6]^ On the other hand, coordinatively unsaturated cobalt(I) complexes have been efficiently employed in small molecule or inert bond activations[^3b^, ^7^] as well as catalytic transformations.^[8]^

Cobalt‐mediated hydrometallation reactions provide a convenient access to synthetically useful reagents,[^1a^, ^9^] and in particular the catalytic hydroboration of alkynes allows for the synthesis of alkenyl boronates and related species.^[10]^ In case of terminal alkynes, the potential formation of three different isomers with either α‐, β‐(*Z*)‐ or β‐(*E*)‐configuration necessitates control of regio‐ and stereoselectivity. Chirik and co‐workers established bis(imino)pyridine cobalt complexes as effective catalysts for the stereodivergent hydroboration of terminal alkynes to (*Z*)‐configured alkenyl boronates, which stem from a *syn*‐hydrometallation step of a alkynyl boronate intermediate.^[11]^ For the cobalt‐mediated synthesis of (*E*)‐alkenyl boronates, both an isolated α‐diimine cobalt(II) hydride complex^[5]^ and a bench‐stable cobalt(II) coordination polymer^[12]^ have been recently reported.

The α‐selective hydroboration of terminal alkynes to produce α‐borylated alkenes provides access to valuable reagents for the synthesis of 1,1‐disubstituted, terminal alkenes, a common structural motif in a range of bioactive compounds.^[13]^ Current synthetic methods include a Ni‐catalyzed hydroalumination procedure,^[14]^ NHC‐Cu‐mediated hydroboration reactions with diboron reagents in the presence of *tert*‐butoxide salts,[^13^, ^15^] as well as a Pd‐catalyzed hydroborylation approach,^[16]^ requiring elevated reaction temperatures of 80 °C. In view of the structural relevance of α‐borylated, terminal alkenes, the need for further synthetic strategies under mild conditions seems apparent.

Based on our previous work on boxmi^[17]^ complexes in catalytic transformations,^[18]^ we chose the synthesis of alkyl complexes ^R′,R^boxmiCoCH_2_SiMe_3_ (**1**) as starting point for our investigation (Scheme 1).

**Scheme 1:**
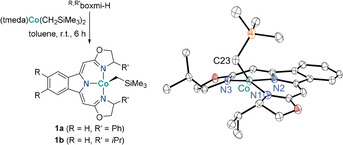
*Left*: Synthesis of cobalt(II) alkyl complexes **1**. *Right*: Molecular structure of ^H,*i*Pr^boxmiCoCH_2_SiMe_3_ (**1 b**) in the solid‐state. Displacement ellipsoids set at 30 % probability level, hydrogen atoms omitted for clarity, only one of two independent molecules shown. Selected bond lengths [Å] and angles [°], values for the second independent molecule are given in square brackets: Co–C23 1.963(7) [1.981(7)], Co–N1 1.905(6) [1.891(6)], Co–N2 1.963(6) [1.947(5)], Co–N3 1.929(6) [1.917(6)], N2‐Co‐C23 134.5(3) [130.7(3)], N1‐Co‐N3 153.5(2) [160.1(2)].^[30]^

Treating dialkyl precursor (tmeda)Co(CH_2_SiMe_3_)_2_ (tmeda=*N*,*N*,*N*′,*N*′‐tetramethylethylen‐diamine) with equimolar amounts of protio‐ligand ^R′,R^boxmi‐H provided facile access to cobalt(II) *high‐spin* complexes **1** in quantitative yields. The molecular structure of the four‐coordinate distorted tetrahedral precatalyst **1 b** was established by X‐Ray diffraction, with all Co‐N and Co‐C23 bond lengths being in good agreement with similar 3*d* metal boxmi *high‐spin* complexes.[^18e^, ^19^] The cobalt complexes **1** were found to be suitable precatalysts for the selective hydroboration of alkynes. In a first assay, we reacted 1‐ethynyl‐4‐fluorobenzene (**2 a**) with two equivalents pinacolborane (HBPin) in the presence of 2.5 mol % ^H,Ph^boxmiCoCH_2_SiMe_3_ (**1 a**), finding the selective formation of mono‐borylated products **3 a** in a remarkable ratio of **α‐3 a**:**β‐(*E*)‐3 a**=8:1 (Figure 1, top).

**Figure 1:**
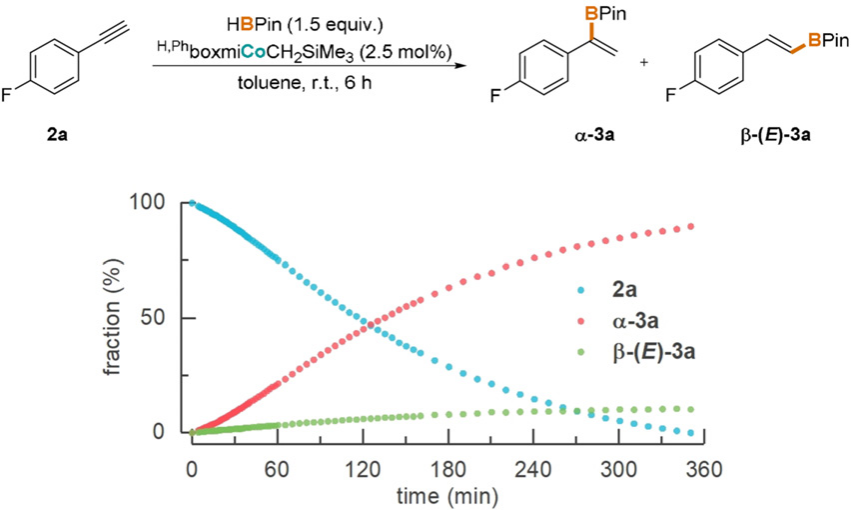
Conversion profile for a cobalt‐catalyzed hydroboration of 1‐ethinyl‐4‐fluorobenzene ([Alkyne]_0_/M=0.22, [HBPin]_0_/M=0.33, [Co]_0_/M 0.005, room temperature, toluene) monitored by in situ ^19^F NMR spectroscopy. Relative amounts in reaction mixture given.

Notably, the resulting synthetically useful, branched regioisomer **α‐3 a** as main product is in stark contrast to previously reported procedures for cobalt‐catalyzed hydroborations, all of which favor the linear *anti*‐Markovnikov products with (*E*)‐[^5^, ^12^] or (*Z*)‐selectivity.^[11]^ As monitored by in situ ^19^F NMR spectroscopy (Figure 1, bottom), the *c*‐*t* profile for the hydroboration by alkyl complex **1 a** features a distinct sigmoidal course, indicating a significant induction period prior to the reduction process.

Upon treatment of the alkyl complexes **1** with an excess of pinacolborane, slow consumption of HBPin and the emergence of the σ‐bond metathesis product Me_3_SiCH_2_BPin were detected. From a mass balance point of view, the rise of the latter implies the presence of a cobalt hydride complex (Scheme 2, middle), the instability of which precluded its pure isolation. However, the reaction of ^H,*i*Pr^boxmiCoCH_2_SiMe_3_ with pinacolborane in the presence of an internal alkyne, namely 1‐phenyl‐1‐propyne, resulted in the generation of vinyl complex **4** (Scheme 2, top) which confirms the in situ generation of a cobalt hydride complex. More importantly, the reaction outcome also renders this insertion reaction a probable elementary step in the catalytic cycle for the cobalt‐catalyzed hydroboration. Although alkyne insertions into a cobalt hydride bond are frequently postulated for various transformations,^[20]^ the isolation of catalytically relevant cobalt(II) vinyl complexes remains scarce. This is in stark contrast to the fact that the fundamental feasibility of this transformation has long been known, in fact also for terminal alkynes.^[21]^ Notably, a Markovnikov‐selective hydrometallation step was very recently proposed to facilitate the cobalt‐catalyzed sequential hydrogenation/hydrohydrazidation of terminal alkynes.^[22]^ There was no evidence for the formation of a cobalt boryl species or the occurrence of an oxidative addition step as discussed for similar catalysts.[^11^, ^23^]

**Scheme 2:**
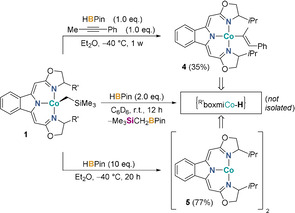
Reaction of precatalyst ^H,R^boxmiCoCH_2_SiMe_3_ (**1**) with stoichiometric amounts of pinacolborane as well as the internal alkyne 1‐phenyl‐1‐propyne yielding vinyl complex **4** (*top*), pinacolborane to generate the non‐isolable hydrido key intermediate (*middle*) as well as an excess of pinacolborane to give the dinuclear Co^I^ complex **5** (*bottom*).

The *d* ^7^ *low‐spin* vinyl complex **4** adopts a distorted square‐planar coordination sphere with all bond metrics being in the expected range^[19]^ (Figure 2, left). In solution, well‐resolved paramagnetic ^1^H and ^13^C NMR spectra with comparatively small shift dispersions are consistent with the *C*_2_ symmetry of the pincer complex in agreement with other square‐planar cobalt(II) compounds (SI).^[24]^

**Figure 2:**
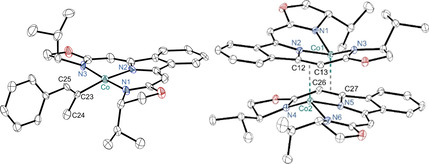
Molecular structures of ^H,***i***Pr^boxmiCoC(Me)C(H)Ph (**4**, *left*) and [^H,*i*Pr^boxmiCo]_2_ (**5**, *right*). Displacement ellipsoids set at 30 % (**4**) and 50 % (**5**) probability level, hydrogen atoms omitted for clarity, only one of four independent molecules shown for **4**. Selected bond lengths [Å] and angles [°], given as range for all independent molecules in case of **4**; **4**: Co–C23 1.922(11)…1.956(11), Co–N1 1.921(7)…1.942(7), Co–N2 1.966(8)…1.994(8), Co–N3 1.916(7)…1.934(7); N2‐Co‐C23 162.6(4)…167.3(4), N1‐Co‐N3 171.5(4)…174.8(4). **5**: Co1–center(C26‐C27) 2.029(2), Co2–center(C12‐C13) 2.047(2), Co1–N1 2.002(3), Co1–N2 1.976(3), Co1–N3 2.029(3), Co2–N4 2.062(3), Co2–N5 1.987(3), Co2–N6 2.009(3), C12–C13 1.412(4), C26–C27 1.420(4); N2‐Co1‐center(C26‐C27) 104.6(1), N1‐Co1‐N3 133.84(12), N5‐Co2‐center(C12‐C13) 106.0(1), N4‐Co2‐N6 139.60(12).^[30]^

All attempts to isolate the hydrido species failed, due to its selective but irreversible transformation, formally resulting from H_2_ elimination, to a coordinatively unsaturated cobalt(I) complex **5** (Scheme 2, bottom).[^3c^, ^3e^]

The molecular structure of cobalt(I) complex **5** was determined by X‐ray diffraction to be dimeric in the solid‐state (Figure 2, right). With the cobalt centers positioned slightly above the boxmi ligand plane, additional weak interactions of the metal to a C=C double bond of theneighboring boxmi ligand are observed (*d*=2.029 Å and 2.047 Å) and give rise to a distorted tetrahedral coordination sphere around the cobalt centers. With T‐shaped geometries being scarce,[^3e^, ^25^] this form of dimerization constitutes a hitherto not observed stabilization mode for this type of complex, as the majority of coordinatively unsaturated cobalt(I) compounds is further stabilized by additional arene,[^7b^, ^26^] carbon monoxide,^[3d]^ or nitrogen ligands^[3a]^ or undergo ligand rearrangements upon dimerization.^[27]^

The magnetic moment of complex **5** was found to be *μ*_eff_=5.07 μ_B_ at room temperature in solution, indicating a moderate antiferromagnetic coupling between the two cobalt centers, Accordingly, an antiferromagnetic coupling constant of *J*_AFC_=−54 cm^−1^ was derived from solid‐state (SQUID) experiments (see SI). A strong temperature‐dependence of the paramagnetic ^1^H NMR spectra was observed, resulting in a non‐Curie and coalescence behavior. As a consequence, the signal number is diminished from 32 in the low temperature limit, which is consistent with a nonsymmetrical dimer, to eight at elevated temperatures which can be attributed to a reversible dynamic dissociation giving the monomeric form dominating at elevated temperatures (see SI).

Apart from the absence or presence of an internal alkyne, both vinyl species **4** and the cobalt(I) species **5** were prepared under similar conditions, indicating that in the presence of the alkyne the insertion reaction to give **4** is kinetically favored over H_2_ elimination giving **5** and, thus, highlighting the reactivity of the cobalt(II) hydride species. Moreover, complex **5** was found to operate as a hydroboration catalyst in its own right, albeit with a distinctively different kinetic trace (see SI). Its reactivity towards pinacolborane was found to be sluggish but reaction with stoichiometric amounts of pinacolborane and 1‐phenyl‐1‐propyne regenerated vinyl complex **4** as main product (see SI).

Several catalytic and stoichiometric control experiments were conducted to obtain insight into the main catalytic pathway (Scheme 3). The successful hydroboration of an internal alkyne, such as 1‐phenyl‐1‐propyne, indicated that alkynyl species were not key intermediates. In this reaction primarily the **α‐(*Z*)** and **β‐(*Z*)** product in a 1:2 ratio and only trace amounts of the formally *anti*‐borylated products were observed (Scheme 3 a). Deuterium labeling studies were performed for the cobalt‐catalyzed reaction of phenyl acetylene with pinacolborane to probe potential hydride transfer pathways (Scheme 3 b). Both the treatment of PhCCD with 1.1 equivalents of HBPin as well as the same reaction with reversely deuterated reagents led to complete proton/deuterium scrambling with all possible H/D isomers equally represented. This indicates that the selectivity‐determining step is reversible, revealing a fast equilibrium between the cobalt hydride complex and the insertion product. In addition, these results imply that there is a rapid equilibration between the (*E*)‐ and (*Z*)‐vinyl species. Such behavior has been reported previously by Chirik^[11]^ and Ojima^[28]^ for related cobalt and rhodium complexes and was proposed to occur via a zwitterionic carbene intermediate. Finally, reacting isolated vinyl complex **4** with stoichiometric amounts of pinacolborane yielded the anticipated σ‐bond metathesis product as sole species after protolytic workup, demonstrating the feasibility of this elementary reaction within the given context and its importance for the catalytic process (Scheme 3 c).

**Scheme 3:**
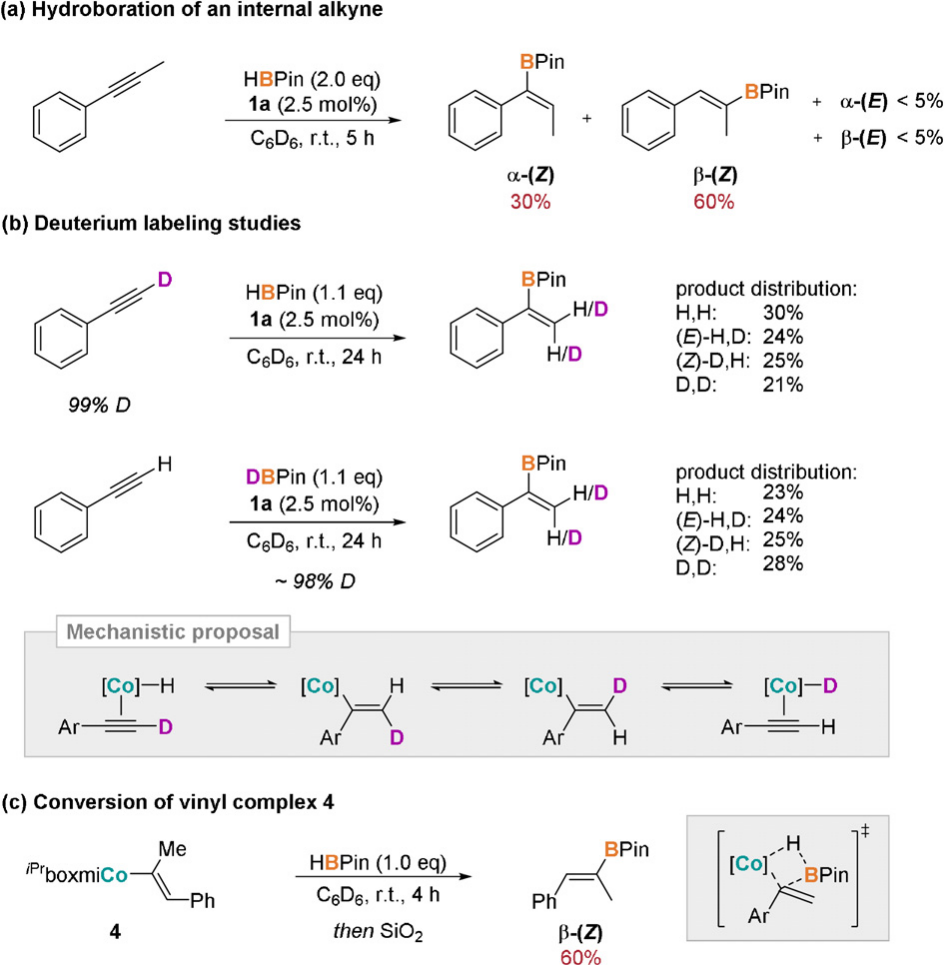
Mechanistic control experiments. In all schematic illustrations, [**Co**] denotes the complex fragment ^H,R^boxmiCo; a) product distribution given; b) product distribution with respect to **α**‐**3 b** given; c) NMR yield given.

Based on these findings, a catalytic cycle consisting of reversible alkyne insertion into a cobalt(II) hydride and subsequent product release via σ‐bond metathesis is proposed for the cobalt‐catalyzed hydroboration of alkynes (Scheme 4).

**Scheme 4:**
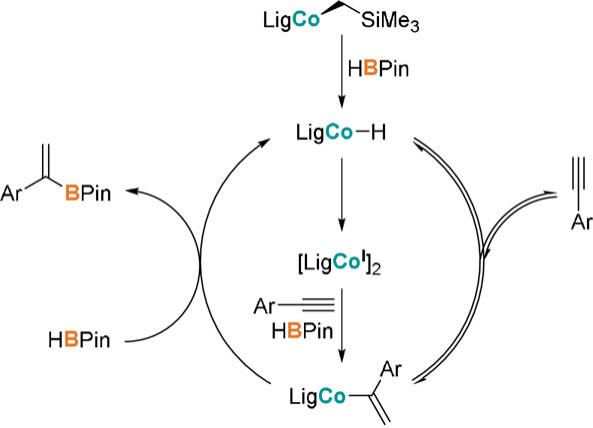
Proposed mechanistic cycle for the cobalt‐catalyzed hydroboration of alkynes.

To probe the synthetic potential of the cobalt‐catalyzed hydroboration of terminal alkynes a screening of differently substituted boxmi ligands and reaction conditions established the phenyl‐substituted complex **1 a** as the most efficient pre‐catalyst, allowing catalyst loadings as low as 0.5 mol % for full conversion within 6 h at room temperature (Table 1, for details see SI).

**Table 1:** Screening of reaction conditions for the hydroboration of terminal alkynes. 
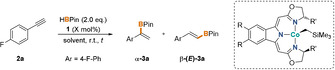

#	R	R′	solvent	catalyst [mol %]	*t* [h]	conv. [%]^[a]^	α:β^[a]^
1	H	Ph	toluene	2.5	5	>99	92:8
2	Me	Ph	toluene	2.5	5	>99	91:9
3	Ph	Ph	toluene	2.5	5	>99	92:8
4	H	*i*Pr	toluene	2.5	3	>99	86:14
5	H	*t*Bu	toluene	2.5	14	31	74:26
6	Me	Bn	toluene	2.5	3	>99	77:23
7	H	Ph	tol/*n*‐hex^[b]^	0.5	6	>99	91:9

[a] Determined by in situ ^19^F NMR spectroscopy. [b] Solvent mixture of toluene and *n*‐hexane (*v*:*v*=1:5). [c] Identical results were obtained for isolated and in situ generated precatalysts; reactions were performed at 0.1 mmol scale.

With these optimized conditions in hand, we explored the applicability of the cobalt‐catalyzed method and subjected a variety of alkynes to the Markovnikov‐selective hydroboration with the phenyl‐substituted alkyl complex **1 a** (Scheme 5, top). Both electron‐withdrawing substituents (**3 a,c,d**) and the presence of *ortho*‐substituents (**3 h,l,m**) appeared to be beneficial for high α:β ratios (up to 97:3), thereby matching the selectivities obtained from previous approaches.^[15]^ Regarding terminal alkyl alkynes, cyclohexyl derivative **2 n** turned out to be detrimental to a satisfactory enrichment of the α‐isomer, whereas only sluggish hydroboration was observed for the trimethylsilyl substituted alkyne **2 o**. In the case of ester, cyano, or nitro groups being attached to an aryl alkyne, slow conversions of the substrate were accompanied by the formation of inseparable side products (see SI), preventing the pure isolation of the target compounds. As mentioned above, internal alkynes (**2 p**) were also tolerated in this cobalt‐catalyzed hydroboration protocol, albeit with inverted α:β selectivity. In addition, compound **β‐3 p** was obtained as the major product from the catalytic conversion of 3‐phenyl‐1‐propyne with pinacolborane, demonstrating the tendency of propargylic substrates to form the respective internal alkene derivatives (see SI for details).

**Scheme 5:**
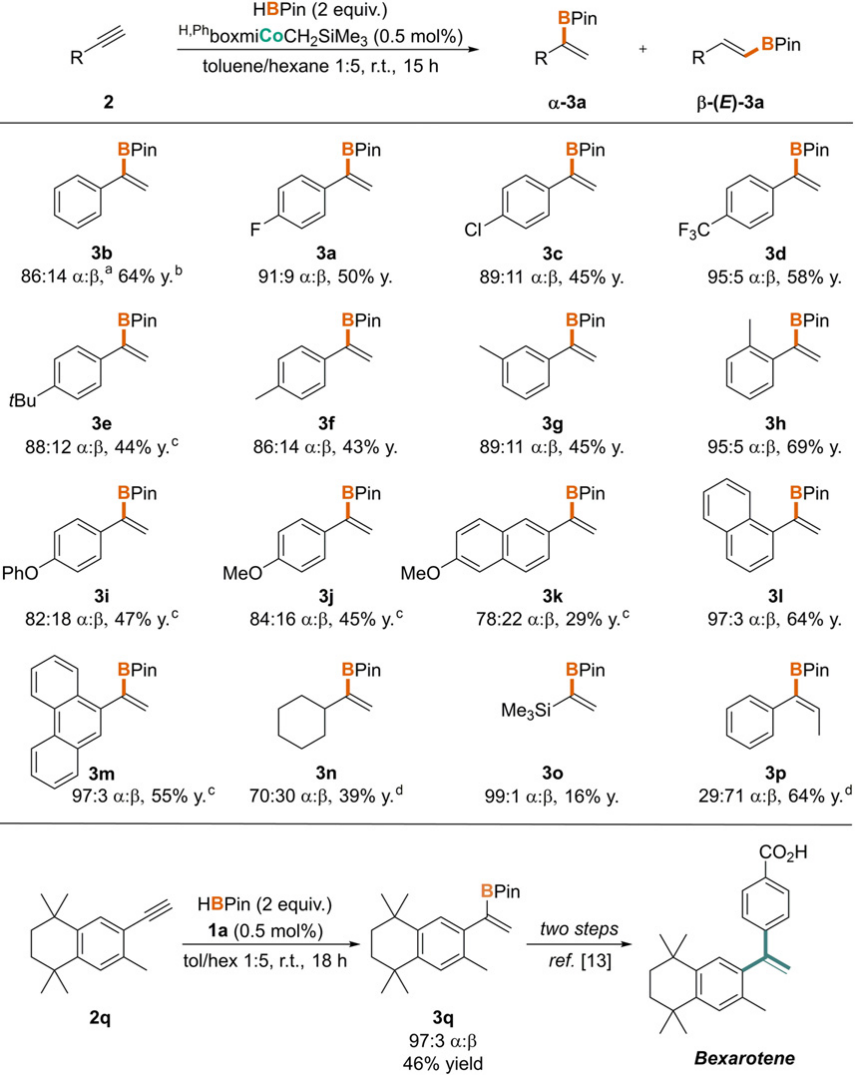
*Top*: Substrate scope of the cobalt‐catalyzed α‐selective hydroboration of terminal alkynes. Reaction conditions: 300 μmol alkyne, 600 μmol HBPin, 0.5 mol % **1 a**. [a] α:β‐(E) ratio was determined by ^1^H NMR spectroscopy before purification; estimated error: ±2 %. [b] Yield of enriched α‐product. [c] 1 mol % catalyst loading. [d] Yield of isomer mixture. *Bottom*: Formal synthesis of Bexarotene by cobalt‐catalyzed α‐selective hydroboration.

The facile access to the respective α‐isomers in the case of aryl alkynes prompted us to apply this cobalt‐based method in the synthesis of a biologically active compound with the given structural core motif (Scheme 5, bottom). Reacting the readily accessible alkyne **2 q** in the presence of precatalyst **1 a** with pinacolborane yielded the borylated alkene **3 q** in a selectivity of 97:3, allowing for the isolation of the 1,1‐disubstituted alkene **α‐3 q** in 46 % yield. Following a literature procedure,^[13]^ this compound can be converted in two steps into Bexarotene, a pharmaceutical for the treatment of cutaneous T‐cell lymphomas.^[29]^

In conclusion, the boxmi‐cobalt(II) alkyl complexes effectively catalyze the Markovnikov‐selective hydroboration of terminal alkynes, furnishing the respective branched alkenyl boronate esters in moderate to good yields (α:β ratio up to 97:3). The facile accessibility of a coordinatively unsaturated cobalt(I) compound demonstrates the marked one‐electron redox chemistry of 3*d* metal complexes, an off‐cycle reactivity in the case at hand but opening up new perspectives for future applications in homogeneous catalysis.

## Conflict of interest

The authors declare no conflict of interest.

## Supporting information

As a service to our authors and readers, this journal provides supporting information supplied by the authors. Such materials are peer reviewed and may be re‐organized for online delivery, but are not copy‐edited or typeset. Technical support issues arising from supporting information (other than missing files) should be addressed to the authors.

Supplementary
