# Supplementary material for: Reaction Pathways and Redox States in α‐Selective Cobalt‐Catalyzed Hydroborations of Alkynes
Source: Angew Chem Int Ed Engl. 2020 Oct 15;59(51):23010–4. doi: 10.1002/anie.202009625 (PMC7756293; doi:10.1002/anie.202009625)
Supplement: Supplementary file 1 — Supplementary [file ANIE-59-23010-s001.pdf]

Angewandte Chemie
Eine Zeitschrift der Gesellschaft Deutscher Chemiker

Supporting Information

Reaction Pathways and Redox States in α-Selective Cobalt-Catalyzed Hydroborations of Alkynes

*Clemens K. Blasius, Vladislav Vasilenko, Regina Matveeva, Hubert Wadepohl, and Lutz H. Gade**

anie_202009625_sm_miscellaneous_information.pdf

Contents

1 General Informations **S2**

2 Synthetic Procedures **S3**

2.1 Complex Synthesis S3

2.2 Catalytic Procedures S6

2.2.1 Screening of Reaction Conditions S6

2.2.2 Cobalt-Catalyzed Hydroboration of Alkynes S7

2.2.3 Analytical Data S10

2.2.4 Synthesis of a Bexaroten Precursor S16

3 Mechanistic Experiments **S17**

3.1 Precatalyst Activation S17

3.2 Hydroboration of an Internal Alkyne S19

3.3 Deuterium Labeling Studies S20

3.3.1 Evaluation of the H/D Distribution S20

3.3.2 Evidence for H/D Exchange S22

3.4 Conversion of ^{iPr}boxmiCo(C(Me)C(H)Ph) (**4**) S23

3.5 Conversion of [^{iPr}boxmiCo]$_2$ (**5**) S24

3.6 Kinetic Profiles S26

4 X-ray Crystal Structure Determinations **S27**

5 Spectroscopic Data **S29**

5.1 Temperature-Dependent Analysis of [^{iPr}boxmiCo]$_2$ (**5**) S29

5.2 Squid Magnetometry S31

6 NMR Data **S32**

References **S55**

1 General Informations

All manipulations, except when indicated otherwise, were carried out under exclusion of air and moisture using standard Schlenk and glovebox techniques. As inert gas, Argon 5.0, purchased from Messer Group GmbH, was used after drying over Granusic phosphorus pentoxide granulate. Solvents were dried over activated alumina columns using a solvent purification system (M. Braun SPS 800) or according to standard literature-known methods and stored in glass ampules under an argon atmosphere.[1] Toluene was distilled from sodium, *n*-pentane from sodium/potassium alloy, and tetrahydrofuran, benzene and *n*-hexane from potassium. The same procedures were used to dry the deuterated solvents. Degassed solvents and liquid substrates were obtained by three successive freeze-pump-thaw-cycles. NMR spectra were recorded on Bruker Avance (400 MHz, 600 MHz) instruments. Chemical shifts (δ) are reported in parts per million (ppm) and are referenced to residual proton solvent signals or carbon resonances.[2,3] $BF_3 \cdot OEt_2$ (^{11}B), CCl_3F (^{19}F) and $SiMe_4$ (^{29}Si) were used as external standard. Mass spectra were acquired on a Bruker ApexQe hybrid 9.4 T FT-ICR (ESI) or a JEOL AccuTOF GCx time-of-flight (EI, LIFDI) spectrometer at the mass spectrometry facility of the Institute of Organic Chemistry at the University of Heidelberg. Elemental analyses were carried out in the Microanalysis Laboratory of the Heidelberg Chemistry Department on a vario MICRO cube (Elementar). All ^{R}boxmi-H ligands were synthesized according to literature procedures.[4] (tmeda)Co(CH_2SiMe_3)$_2$ was synthesized either according to a literature procedure[5] or following a method adapted from the synthesis of the iron congener.[6] Alkynes **7c**[7] and **7d**[8] were synthesized according to literature procedures. All other substrates and reagents were obtained from commercial suppliers and were used without further purification. All cobalt salts were purchased with a trace metal purity of 99.99 % or higher.

2 Synthetic Procedures

2.1 Complex Syntheses

Synthesis of ^{R}boxmiCo(CH_2SiMe_3) (1)

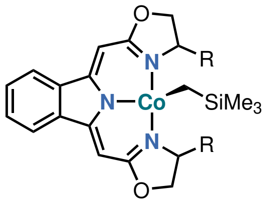

GP1. Equimolar amounts of (tmeda)Co(CH_2SiMe_3)$_2$ (usually 250 µmol, 1.0 eq.) and $^{R',R}$boxmi-H (1.0 eq.) were suspended in toluene (about 5 ml) and stirred at room temperature for 6 h. The resulting brown solution was freed from any volatiles *in vacuo*, yielding the product in quantitative yield. Single crystals of **1b** suitable for X-ray diffraction analysis were obtained by recrystallization from *n*-pentane at −40 °C.

Compound ^{Ph}boxmiCo(CH_2SiMe_3) (1a):

MS (LIFDI$^+$): $[M]^+ = C_{32}H_{33}CoN_3O_2Si^+$, calcd.: 578.16740 found: 578.11683.

EA: calcd.: C: 66.42 %, H: 5.75 %, N: 7.26 %; found: C: 65.80 %, H: 6.19 %, N: 7.26 %.

Magnetic Susceptibility (Evans, Tol-d_8, 295 K): $\mu_{eff} = 4.11\ \mu_B$.

Compound ^{iPr}boxmiCo(CH_2SiMe_3) (1b):

EA: calcd.: C: 61.16 %, H: 7.30 %, N: 8.23 %; found: C: 60.87 %, H: 7.20 %, N: 8.45 %.

Magnetic Susceptibility (Evans, C_6D_6, 295 K): $\mu_{eff} = 4.07\ \mu_B$.

Synthesis of ^{iPr}boxmiCo(C(Me)C(H)Ph) (4)

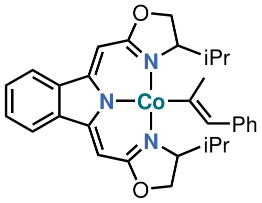

A mixture of ^{iPr}boxmi-H (45.1 mg, 123 µmol, 1.0 eq) and (tmeda)Co(CH_2SiMe_3)$_2$ (43.1 mg, 123 µmol, 1.0 eq) in 2.0 ml Et_2O was stirred at room temperature for 5 h and then filtered. Pinacolborane (17.8 µl, 123 µmol, 1.0 eq) and 1-phenyl-1-propyne (15.4 µl, 123 µmol, 1.0 eq) were sequentially added to the filtrate at −40 °C. The brown solution was directly layered with cold *n*-pentane (approximately 8 ml) and stored at −40 °C for one week, resulting in the formation of dark green crystals. Subsequently, the supernatant was removed and the residue was washed with cold *n*-pentane (2 × 2 ml). The product was obtained as dark green, crystalline solid (23.2 mg, 42.7 µmol, 35 %).

^{1}H NMR (600.13 MHz, C_6D_6, 295 K, paramagnetic): δ [ppm] = 58.84 (s, 2H), 22.10 (s, 2H), 19.74 (s, 2H), 12.89 (s, 6H), 10.96 (s, 2H), 7.93 (s, 1H), 7.66 (s, 2H), 6.50 (s, 6H), 6.22 (s, 2H), 5.45 (s, 2H), 5.06 (s, 2H), −0.80 (*br* s, 2H), −27.90 (s, 1H), −55.21 (s, 3H).

^{13}C NMR (150.90 MHz, C_6D_6, 295 K, paramagnetic): δ [ppm] = 225.4 (s), 217.5 (d, J = 151.0 Hz), 212.7 (s), 208.9 (s), 187.7 (s), 138.0 (d, J = 155.5 Hz), 120.6 (d, J = 154.7 Hz), 111.2 (d, J = 160.1 Hz), 105.0 (d, J = 157.0 Hz), 94.8 (t, J = 148.0 Hz), 36.3–33.9 (m), 33.9–31.8 (m), −2.0 (s), −121.7 (s), −152.8 (s).

MS (LIFDI$^+$): [M]$^+$ = $C_{31}H_{35}CoN_3O_2^+$ (11 %), calcd.: 540.20613 found: 540.2.
[M+O]$^+$ = $C_{31}H_{35}CoN_3O_3^+$ (68 %), calcd.: 556.20104 found: 556.2.

Magnetic Susceptibility (Evans, C_6D_6, 295 K): μ_{eff} = 2.21 μ_B.

EA: calcd.: C: 68.88 %, H: 6.53 %, N: 7.77 %; found: C: 68.80 %, H: 6.71 %, N: 8.07 %.

Synthesis of $[^{iPr}boxmiCo]_2$ (5)

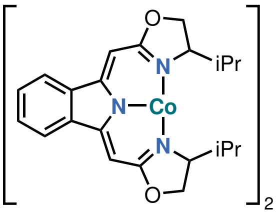

A mixture of ^{iPr}boxmi-H (100.0 mg, 273.6 µmol, 1.0 eq) and (tmeda)Co(CH_2SiMe_3)$_2$ (95.7 mg, 274 µmol, 1.0 eq) was dissolved in 3.0 ml Et_2O and stirred at room temperature for 5 h. The reaction mixture was then filtered and the filtrate was cooled to −40 °C, before neat pinacolborane (397 µl, 2.74 mmol, 10.0 eq) was added in one portion. The brown solution was directly layered with cold *n*-pentane (approximately 8 ml) and stored at −40 °C for 1 d, resulting in the formation of black crystals. Subsequently, the supernatant was removed and the residue was washed with cold *n*-pentane (2 × 2 ml). The mother liquor was freed from any volatiles *in vacuo* and recrystallized from Et_2O and *n*-pentane at −40 °C, yielding a second product batch. The product was obtained as black, crystalline solid (89.2 mg, 105 µmol, 77 %).

MS (LIFDI$^+$): $[M]^+ = C_{22}H_{26}CoN_3O_2^+$, calcd.: 423.1357 found: 423.2.

Magnetic Susceptibility (Evans, Tol-d_8, 295 K): $\mu_{eff} = 5.07\ \mu_B$.

EA: calcd.: C: 62.41 %, H: 6.19 %, N: 9.92 %; found: C: 62.28 %, H: 6.43 %, N: 9.93 %.

2.2 Catalytic Procedures

2.2.1 Screening of Reaction Conditions

Table S1: Screening of reaction conditions for the cobalt-catalyzed hydroboration of terminal alkynes.

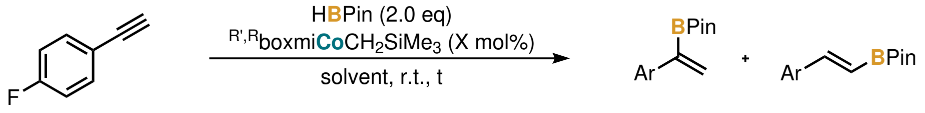

2a α-**3a** β-**3a**

#[a]	R'	R	solvent[b]	mol%	t_1 [h]	conv.$_1$ [%][c]	t_2 [h]	conv.$_2$ [%][c]	α:β[c]
1[d]	H	Ph	toluene	2.5	0.5	33	5	>99	92:8
2[d]	Me	Ph	toluene	2.5	0.6	33	5	>99	91:9
3[d]	Ph	Ph	toluene	2.5	0.7	25	5	>99	92:8
4[d]	H	*i*Pr	toluene	2.5	0.8	99	3	>99	86:14
5[d]	H	*t*Bu	toluene	2.5	0.8	1	14	31	74:26
6[d]	Me	Bn	toluene	2.5	0.9	85	3	>99	77:23
7	H	Ph	MeCN	2.5	1.5	11	16	21	90:10
8	H	Ph	CH_2Cl_2	2.5	1.5	21	16	79	92:8
9	H	Ph	THF	2.5	1.5	45	16	>99	92:8
10	H	Ph	Et_2O	2.5	1.5	>99	16	>99	93:7
11	H	Ph	toluene	2.5	1.5	66	16	>99	92:8
12[e]	H	Ph	*n*-hexane	2.5	1.5	>99	16	>99	92:8
13	H	Ph	t/h 1:0	2.5	0.7	49	1.5	76	90:10
14	H	Ph	t/h 1:1	2.5	0.7	66	1.5	94	91:9
15	H	Ph	t/h 1:5	2.5	0.7	82	1.5	>99	92:8
16	H	Ph	t/h 1:11	2.5	0.7	89	1.5	>99	93:7
17[e]	H	Ph	t/h 0:1	2.5	0.7	>99	1.5	>99	92:8
18	H	Ph	t/h 1:5	2.0	–	–	1.2	>99	92:8
19	H	Ph	t/h 1:5	1.5	–	–	3	>99	93:7
20	H	Ph	t/h 1:5	1.0	–	–	4	>99	92:8
21	H	Ph	t/h 1:5	0.5	–	–	6	>99	91:9

[a] Reactions were performed at 0.1 mmol scale; [b] t/h indicates solvent mixtures of toluene and *n*-hexane (*v*:*v* = x:x); [c] Conversions determined by *in situ* ^{19}F NMR spectroscopy; [d] Precatalyst was synthesized *in situ* according to **GP1**; [e] Precatalyst not completely dissolved.

2.2.2 Cobalt-Catalyzed Hydroboration of Alkynes

General Catalytic Procedure

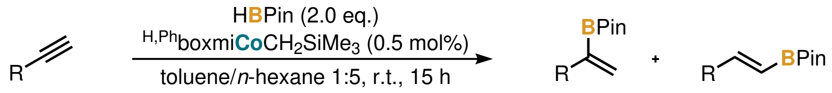

2 α-**3** β-**3**

GP2. A solution of precatalyst **1a** (870 µg,[1] 1.5 µmol, 0.5 mol%, if not stated otherwise) in 1.2 ml toluene/*n*-hexane (*v*/*v* = 1:5) was added to the respective substrate (300 µmol, 1.0 eq). Subsequently, neat pinacolborane (87.2 µl, 600 µmol, 2.0 eq) was added in one portion. After stirring the reaction mixture at room temperature for 15 h, 4 ml of 2 M aqueous HCl were added, the mixture was rapidly extracted with dcm (3 × 5 ml), and the combined organic phases were dried over $NaSO_4$. Alternatively, the reaction mixture was filtered through a short plug of silica, eluted with Et_2O (3 × 2 ml) and freed from any volatiles *in vacuo* (for α-**3e**,**k**,**m**,**u**). The crude product was then purified by column chromatography (*n*-hexane/diethyl ether 19:1, if not stated otherwise). Analytical data for isolated products is provided in Section 2.2.3.

Determination of Regioselectivities

The crude product was analyzed by ^{1}H NMR spectroscopy in order to determine the regioselectivity of the hydroboration reactions. The signals corresponding to the vinylic protons were integrated and compared for α-**3** and β-**3**. The error of this approach was estimated to be ±2%. In case of overlapping signals, signal deconvolution as implemented in Bruker TopSpin 3.0 was carried out in order to determine the specific contributions. An exemplary spectrum illustrating the integration can be found in Figure S1.

[1] In general, stock solutions were prepared with weighable amounts of the catalyst and the relevant quantity was added using an Eppendorf pipette.

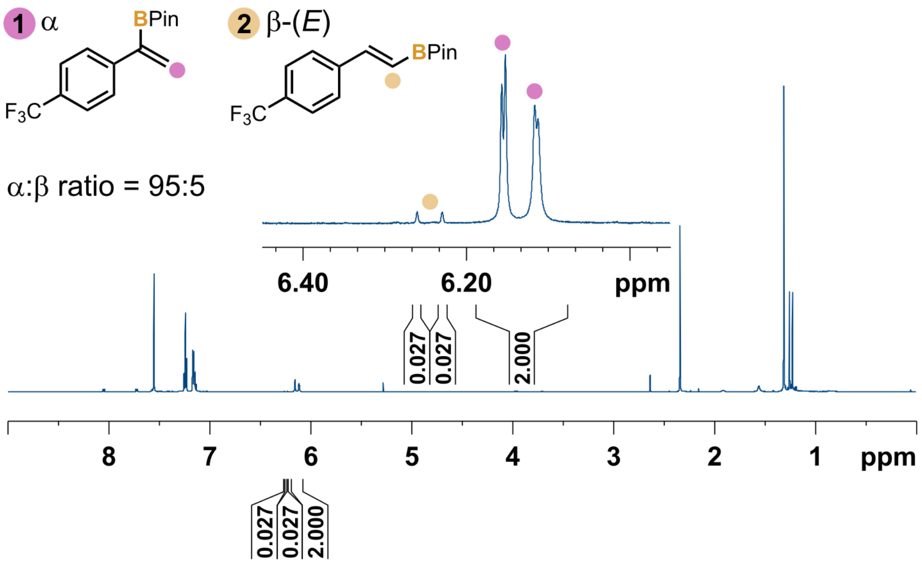

Figure S1: Crude ^{1}H NMR spectrum of α-**3d** for the determination of the product ratio.

Substrate Scope: Omitted Entries

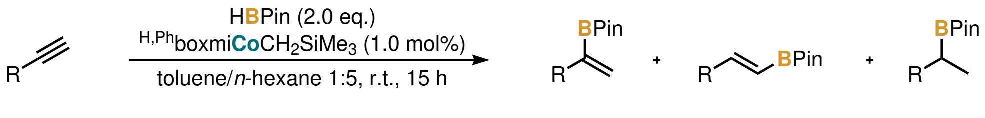

2 α-**3** β-**3** **6**

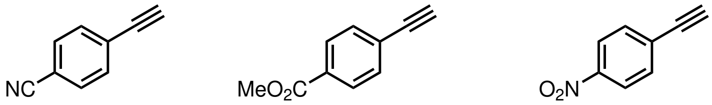

2r ~50% conv. 53:7:40 ratio

2s ~80% conv. 60:9:31 ratio

2t <5% conv.

The hydroboration reaction was carried out following **GP2** with 1.0 mol% catalyst loading (solvent = toluene for **2r**). Subsequently, the reaction mixture was filtered through a short plug of silica, eluted with Et_2O (3 × 2 ml) and freed from any volatiles *in vacuo*. The residue was then analyzed by ^{1}H NMR spectroscopy (ratios given as α-**3**:β-**3**:**6**).

In the hydroboration reaction of alkynes **2r**–**t** the specific functional group remained unaffected. However, in case of substrates **2r** and **2s**, slow conversion of the starting material was accompanied by the formation of the inseparable side product **6**. For alkyne **2t**, only poor conversion was detected.

Hydroboration of Propargylic Substrates

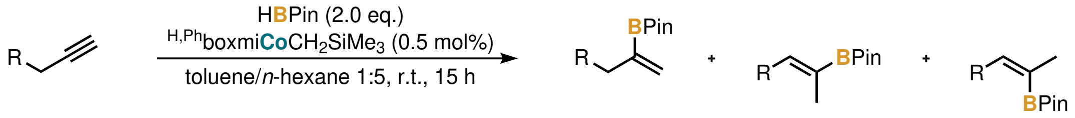

7 — *ter*-**8** + (*Z*)-*int*-**8** (major product) + (*E*)-*int*-**8**

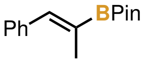

8a

0:92:8 ratio,
70% yield

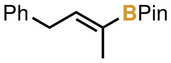

8b

10:67:23 ratio,
65% yield

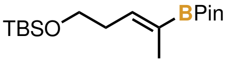

8c

1:80:19 ratio,
73% yield

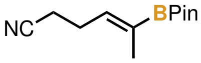

8d

5:62:33 ratio,
67% yield

GP3. A solution of precatalyst **1a** (870 µg, 1.5 µmol, 0.5 mol%) in 1.2 ml toluene/*n*-hexane (*v*/*v* = 1:5) was added to the respective substrate (300 µmol, 1.0 eq). Subsequently, neat pinacolborane (87.2 µl, 600 µmol, 2.0 eq) was added in one portion. After stirring the reaction mixture at room temperature for 15 h, the reaction mixture was filtered through a short plug of silica, eluted with Et_2O (3 × 2 ml) and freed from any volatiles *in vacuo*. The product ratio was determined by ^{1}H NMR spectroscopy (ratios given as *ter*-**8**:(*Z*)-*int*-**8**:(*E*)-*int*-**8**). The crude product was purified by column chromatography (*n*-hexane/diethyl ether 49:1, if not stated otherwise). Yields are given for isomeric mixtures. Analytical data for isolated products is provided in Section 2.2.3.[2]

[2]Note: Compound (*Z*)-*int*-**8a** equals compound (*Z*)-β-**3p**.

2.2.3 Analytical Data

1-Phenylvinylboronic acid pinacol ester (α-3b)

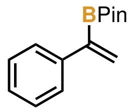

α-**3b**

Known compound.[9,10] Employing **GP2**, reduction of **2b** yields the hydroboration products in ratio α:β = 86:14. The product α-**3b** (along with ~10% β-**3b**) is obtained in 64 % yield. **^{1}H NMR (399.89 MHz, $CDCl_3$, 295 K):** δ [ppm] = 7.52–7.49 (m, 2H), 7.36–7.32 (m, 2H), 7.28–7.24 (m, 1H), 6.15–6.05 (m, 2H), 1.35 (s, 12H). **^{13}C NMR (100.56 MHz, $CDCl_3$, 295 K):** δ [ppm] = 141.5, 131.0, 128.3, 127.3, 127.1, 83.9, 24.9. **^{11}B NMR (128.30 MHz, $CDCl_3$, 295 K):** δ [ppm] = 30.5. **HR-MS (EI$^+$):** $[M]^+$ = $C_{14}H_{19}BO_2^+$, calcd.: 230.1478, found: 230.1474.

1-(4-Fluorophenyl)vinylboronic acid pinacol ester (α-3a)

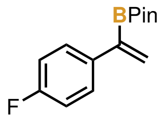

α-**3a**

Known compound.[9,10] Employing **GP2**, reduction of **2a** yielded the hydroboration products in ratio α:β = 91:9. The product α-**3a** (along with ~13% β-**3a**) was obtained in 50 % yield. **^{1}H NMR (600.13 MHz, $CDCl_3$, 295 K):** δ [ppm] = 7.47–7.43 (m, 2H), 7.02–6.98 (m, 2H), 6.05–6.03 (m, 2H), 1.32 (s, 12H). **^{13}C NMR (150.90 MHz, $CDCl_3$, 295 K):** δ [ppm] = 162.3 (d, J = 245.4 Hz), 137.5 (d, J = 3.3 Hz), 130.9 (d, J = 1.4 Hz), 128.9 (d, J = 7.8 Hz), 115.1 (d, J = 21.1 Hz), 84.0 (s), 24.9 (s). **^{19}F NMR (376.27 MHz, $CDCl_3$, 295 K):** δ [ppm] = −116.1. **^{11}B NMR (128.30 MHz, $CDCl_3$, 295 K):** δ [ppm] = 30.4. **HR-MS (EI$^+$):** $[M]^+$ = $C_{14}H_{18}BFO_2^+$, calcd.: 248.13784, found: 248.13829.

1-(4-Chlorophenyl)vinylboronic acid pinacol ester (α-3c)

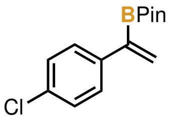

α-**3c**

Known compound.[9] Employing **GP2**, reduction of **2c** yielded the hydroboration products in ratio α:β = 89:11. The product α-**3c** (along with ~10% β-**3c**) was obtained in 45 % yield. **^{1}H NMR (399.89 MHz, $CDCl_3$, 295 K):** δ [ppm] = 7.41–7.37 (m, 2H), 7.27–7.23 (m, 2H), 6.05–6.03 (m, 2H), 1.29 (s, 12H). **^{13}C NMR (100.56 MHz, $CDCl_3$, 295 K):** δ [ppm] = 140.0, 133.0, 131.5, 128.7, 128.4, 84.1, 24.9. **^{11}B NMR (128.30 MHz, $CDCl_3$, 295 K):** δ [ppm] = 30.4. **HR-MS (EI$^+$):** $[M]^+$ = $C_{14}H_{18}BClO_2^+$, calcd.: 264.10829, found: 264.10815.

1-(4-(Trifluoromethyl)phenyl)vinylboronic acid pinacol ester (α-3d)

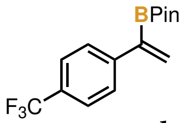

α-3d

Known compound.[9,10] Employing **GP2**, reduction of **2d** yielded the hydroboration products in ratio α:β = 95:5. The product α-**3d** (along with ~4% β-**3d**) was obtained in 58 % yield. **^{1}H NMR (399.89 MHz, CDCl$_3$, 295 K):** δ [ppm] = 7.57 (s, 4H), 6.17 (d, *J* = 2.5 Hz, 1H), 6.13 (d, *J* = 2.5 Hz, 1H), 1.33 (s, 12H). **^{13}C NMR (100.56 MHz, CDCl$_3$, 295 K):** δ [ppm] = 133.2 (s), 127.6 (s), 125.2 (q, *J* = 4.0 Hz), 84.2 (s), 24.9 (s). **^{19}F NMR (376.27 MHz, CDCl$_3$, 295 K):** δ [ppm] = −62.4 **^{11}B NMR (128.30 MHz, CDCl$_3$, 295 K):** δ [ppm] = 30.2. **HR-MS (EI$^+$):** [M]$^+$ = $C_{15}H_{18}BF_3O_2^+$, calcd.: 298.13465, found: 298.13516.

1-(4-*tert*-Butylphenyl)vinylboronic acid pinacol ester (α-3e)

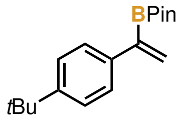

α-3e

Known compound.[11] Employing **GP2** with 1 % catalyst loading, reduction of **2e** yielded the hydroboration products in ratio α:β = 88:12. Column chromatography (*n*-hexane/diethyl ether = 49:1) afforded the product α-**3e** (along with ~10% β-**3e**) in 44 % yield. **^{1}H NMR (600.13 MHz, CDCl$_3$, 295 K):** δ [ppm] = 7.46–7.45 (m, 2H), 7.37–7.35 (m, 2H), 6.09 (d, *J* = 2.8 Hz, 1H), 6.03 (d, *J* = 2.9 Hz, 1H), 1.34 (s, 12H), 1.33 (s, 9H). **^{13}C NMR (150.90 MHz, CDCl$_3$, 295 K):** δ [ppm] = 150.0, 138.5, 130.3, 126.9, 125.3, 83.9, 34.6, 31.5, 24.9. **^{11}B NMR (192.55 MHz, CDCl$_3$, 295 K):** δ [ppm] = 30.5. **HR-MS (EI$^+$):** [M]$^+$ = $C_{18}H_{27}BO_2^+$, calcd.: 286.20986, found: 286.21094.

1-(*p*-Tolyl)vinylboronic acid pinacol ester (α-3f)

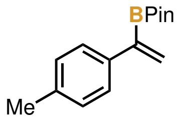

α-3f

Known compound.[9] Employing **GP2**, reduction of **2f** yielded the hydroboration products in ratio α:β = 86:14. The product α-**3f** (along with ~13% β-**3f**) was obtained in 43 % yield. **^{1}H NMR (399.89 MHz, CDCl$_3$, 295 K):** δ [ppm] = 7.40–7.35 (m, 2H), 7.15–7.10 (m, 2H), 6.04 (d, *J* = 2.9 Hz, 1H), 6.00 (d, *J* = 2.9 Hz, 1H), 2.33 (s, 3H), 1.32 (s, 12H). **^{13}C NMR (100.56 MHz, CDCl$_3$, 295 K):** δ [ppm] = 138.7, 136.8, 130.2, 129.0, 127.2, 83.9, 24.9, 21.3. **^{11}B NMR (128.30 MHz, CDCl$_3$, 295 K):** δ [ppm] = 30.4. **HR-MS (EI$^+$):** [M]$^+$ = $C_{15}H_{21}BO_2^+$, calcd.: 244.16291, found: 244.16267.

1-(*m*-Tolyl)vinylboronic acid pinacol ester (α-3g)

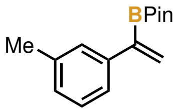

α-**3g**

Known compound.[9] Employing **GP2**, reduction of **2g** yielded the hydroboration products in ratio α:β = 89:11. The product α-**3g** (along with ~10% β-**3g**) was obtained in 45 % yield. **^{1}H NMR (399.89 MHz, $CDCl_3$, 295 K):** δ [ppm] = 7.31–7.25 (m, 2H), 7.23–7.20 (m, 1H), 7.08–7.06 (m, 1H), 6.06–6.04 (m, 2H), 2.36 (s, 3H), 1.33 (s, 12H). **^{13}C NMR (100.56 MHz, $CDCl_3$, 295 K):** δ [ppm] = 141.5, 137.8, 130.8, 128.2, 127.9, 124.6, 83.9, 24.9, 21.7. **^{11}B NMR (128.30 MHz, $CDCl_3$, 295 K):** δ [ppm] = 30.6.

1-(*o*-Tolyl)vinylboronic acid pinacol ester (α-3h)

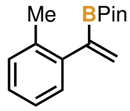

α-**3h**

Known compound.[9,10] Employing **GP2**, reduction of **2h** yielded the hydroboration products in ratio α:β = 95:5. The product α-**3h** (along with ~4% β-**3h**) was obtained in 69 % yield. **^{1}H NMR (399.89 MHz, $CDCl_3$, 295 K):** δ [ppm] = 7.28–7.19 (m, 4H), 6.28 (d, *J* = 3.6 Hz, 1H), 5.91 (d, *J* = 3.5 Hz, 1H), 2.39 (s, 3H), 1.41 (s, 12H). **^{13}C NMR (100.56 MHz, $CDCl_3$, 295 K):** δ [ppm] = 142.8, 135.1, 133.1, 129.8, 128.5, 127.0, 125.9, 83.9, 24.9, 20.3. **^{11}B NMR (128.30 MHz, $CDCl_3$, 295 K):** δ [ppm] = 30.1. **HR-MS (EI$^+$):** $[M]^+$ = $C_{15}H_{21}BO_2^+$, calcd.: 244.1629, found: 244.16197.

1-(4-Phenoxyphenyl)vinylboronic acid pinacol ester (α-3i)

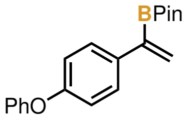

α-**3i**

Employing **GP2** with 1 % catalyst loading, reduction of **2i** yielded the hydroboration products in ratio α:β = 82:18. The product α-**3i** (along with ~5% β-**3i**) was obtained in 47 % yield as colorless solid. **^{1}H NMR (600.13 MHz, $CDCl_3$, 295 K):** δ [ppm] = 7.50–7.48 (m, 2H), 7.35–7.33 (m, 2H), 7.12–7.09 (m, 1H), 7.05–7.03 (m, 2H), 6.98–6.96 (m, 2H), 6.07 (d, *J* = 2.6 Hz, 1H), 6.04 (d, *J* = 2.8 Hz, 1H), 1.34 (s, 12H). **^{13}C NMR (150.90 MHz, $CDCl_3$, 295 K):** δ [ppm] = 157.4, 156.6, 136.5, 130.2, 129.8, 128.6, 123.3, 119.1, 118.6, 84.0, 24.9. **^{11}B NMR (192.55 MHz, $CDCl_3$, 295 K):** δ [ppm] = 30.5. **HR-MS (EI$^+$):** $[M]^+$ = $C_{20}H_{23}BO_3^+$, calcd.: 322.17438, found: 322.17369.

1-(4-Methoxyphenyl)vinylboronic acid pinacol ester (α-3j)

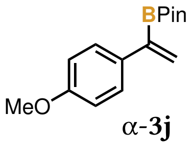

α-**3j**

Known compound.[9] Employing **GP2** with 1 % catalyst loading, reduction of **2j** yielded the hydroboration products in ratio α:β = 84:16. The product α-**3j** (along with ~4% β-**3j**) was obtained in 45 % yield. **^{1}H NMR (399.89 MHz, $CDCl_3$, 295 K):** δ [ppm] = 7.46–7.43 (m, 2H), 6.90–6.85 (m, 2H), 6.02 (d, *J* = 2.8 Hz, 1H), 5.96 (d, *J* = 2.8 Hz, 1H), 3.80 (s, 3H), 1.33 (s, 12H). **^{13}C NMR (100.56 MHz, $CDCl_3$, 295 K):** δ [ppm] = 159.0, 134.1, 129.2, 128.4, 113.8, 83.9, 55.4, 24.9. **^{11}B NMR (128.30 MHz, $CDCl_3$, 295 K):** δ [ppm] = 30.7. **HR-MS (EI$^+$):** $[M]^+$ = $C_{15}H_{21}BO_3^+$, calcd.: 260.15783, found: 260.15691.

1-(6-Methoxynaphthalen-2-yl)vinylboronic acid pinacol ester (α-3k)

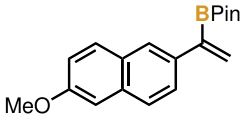

α-**3k**

Employing **GP2** with 1 % catalyst loading, reduction of **2k** yielded the hydroboration products in ratio α:β = 78:22. Column chromatography (*n*-hexane/diethyl ether = 49:1) afforded the product α-**3k** in 29 % yield as off-white solid. **^{1}H NMR (600.13 MHz, $CDCl_3$, 295 K):** δ [ppm] = 7.90 (s, 1H), 7.74 (d, *J* = 8.7 Hz, 1H), 7.69 (d, *J* = 8.5 Hz, 1H), 7.61 (dd, *J* = 8.5 Hz, *J* = 1.6 Hz, 1H), 7.13–7.11 (m, 2H), 6.19 (d, *J* = 2.6 Hz, 1H), 6.11 (d, *J* = 2.8 Hz, 1H), 3.92 (s, 3H), 1.36 (s, 12H). **^{13}C NMR (150.90 MHz, $CDCl_3$, 295 K):** δ [ppm] = 157.6, 136.7, 133.9, 130.5, 129.9, 129.1, 126.7, 126.2, 126.1, 118.7, 105.7, 84.0, 55.4, 25.0. **^{11}B NMR (192.55 MHz, $CDCl_3$, 295 K):** δ [ppm] = 30.7. **HR-MS (EI$^+$):** $[M]^+$ = $C_{19}H_{23}BO_3^+$, calcd.: 310.17348, found: 310.17327.

1-(Naphthalen-1-yl)vinylboronic acid pinacol ester (α-3l)

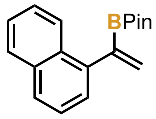

α-**3l**

Employing **GP2**, reduction of **2l** yielded the hydroboration products in ratio α:β = 97:3. The product α-**3l** (along with ~1% β-**3l**) was obtained in 67 % yield as yellow solid. **^{1}H NMR (399.89 MHz, $CDCl_3$, 295 K):** δ [ppm] = 7.88–7.82 (m, 2H), 7.75 (d, *J* = 8.2 Hz, 1H), 7.47–7.40 (m, 3H), 7.29 (dd, *J* = 7.0 Hz, *J* = 1.3 Hz, 1H), 6.32 (d, *J* = 3.6 Hz, 1H), 5.98 (d, *J* = 3.6 Hz, 1H), 1.28 (s, 12H). **^{13}C NMR (100.56 MHz, $CDCl_3$, 295 K):** δ [ppm] = 141.1, 134.0, 133.7, 131.6, 128.3, 127.3, 126.1, 125.8, 125.6, 125.4, 125.3, 84.0, 24.8. **^{11}B NMR (128.30 MHz, $CDCl_3$, 295 K):** δ [ppm] = 30.4. **HR-MS (EI$^+$):** $[M]^+$ = $C_{18}H_{21}BO_2^+$, calcd.: 280.16291, found: 280.16436.

1-(Phenanthren-9-yl)vinylboronic acid pinacol ester (α-3m)

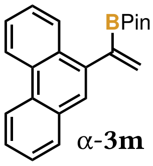

α-**3m**

Employing **GP2** with 1 % catalyst loading, reduction of **2m** yielded the hydroboration products in ratio α:β = 97:3. Column chromatography (*n*-hexane/diethyl ether = 49:1) afforded the product α-**3m** in 55 % yield as yellow oil. **^{1}H NMR (600.13 MHz, $CDCl_3$, 295 K):** δ [ppm] = 8.72 (d, *J* = 8.3 Hz, 1H), 8.67 (d, *J* = 8.2 Hz, 1H), 7.90 (d, *J* = 8.1 Hz, 1H), 7.86 (d, *J* = 7.7 Hz, 1H), 7.66–7.55 (m, 5H), 6.35 (d, *J* = 3.6 Hz, 1H), 6.09 (d, *J* = 3.6 Hz, 1H), 1.28 (s, 12H). **^{13}C NMR (150.90 MHz, $CDCl_3$, 295 K):** δ [ppm] = 140.0, 133.7, 132.2, 131.3, 130.5, 130.2, 128.7, 126.8, 126.7, 126.4, 126.2, 126.1, 125.5, 123.0, 122.6, 84.1, 24.8. **^{11}B NMR (192.55 MHz, $CDCl_3$, 295 K):** δ [ppm] = 30.5. **HR-MS (EI^+):** $[M]^+$ = $C_{22}H_{23}BO_2^+$, calcd.: 330.17856, found: 330.17883.

1-Cyclohexylvinylboronic acid pinacol ester (α-3n)

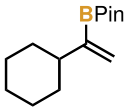

α-**3n**

Known Compound.[12] Employing **GP2**, reduction of **2n** yielded the hydroboration products in ratio α:β = 70:30. Column chromatography (*n*-hexane) afforded the product α-**3n** (along with ~30% β-**3n**) in 39 % yield. **^{1}H NMR (399.89 MHz, $CDCl_3$, 295 K):** δ [ppm] = 5.70 (d, *J* = 3.1 Hz, 1H), 5.54 (d, *J* = 2.1 Hz, 1H), 2.11–2.06 (m, 1H), 1.76–1.63 (m, 5H), 1.35–1.08 (m, 17H). **^{13}C NMR (100.56 MHz, $CDCl_3$, 295 K):** δ [ppm] = 160.0, 126.0, 83.3, 42.9, 32.6, 26.8, 26.5, 24.9. **^{11}B NMR (128.30 MHz, $CDCl_3$, 295 K):** δ [ppm] = 30.4. **HR-MS (EI^+):** $[M]^+$ = $C_{14}H_{25}BO_2^+$, calcd.: 236.19421, found: 236.19337.

1-Trimethylsilylvinylboronic acid pinacol ester (α-3o)

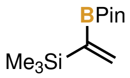

α-**3o**

Known compound.[13] Employing **GP2**, reduction of **2o** yielded the hydroboration products in ratio α:β = 99:1. The product α-**3o** was obtained in 16 % yield. **^{1}H NMR (399.89 MHz, $CDCl_3$, 295 K):** δ [ppm] = 6.58 (d, *J* = 5.4 Hz, 1H), 6.22 (d, *J* = 5.0 Hz, 1H), 1.25 (s, 12H), 0.09 (s, 9H). **^{13}C NMR (100.56 MHz, $CDCl_3$, 295 K):** δ [ppm] = 143.3, 83.1, 24.9, −1.2. **^{11}B NMR (128.30 MHz, $CDCl_3$, 295 K):** δ [ppm] = 30.9.

(Z)-(1-Phenylprop-1-en-2-yl)boronic acid pinacol ester ((Z)-*int*-8a)

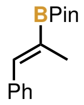

(Z)-*int*-**8a**

Known compound.[14] Employing **GP3**, reduction of alkyne **7a** yielded the hydroboration products (Z)-*int*-**8a** and (E)-*int*-**8a** in a ratio of 92:8. The products **8a** were obtained as isomeric mixture in 70 % yield. **^{1}H NMR (399.89 MHz, $CDCl_3$, 295 K):** [for major isomer] δ [ppm] = 7.41–7.33 (m, 4H), 7.29–7.17 (m, 2H), 2.01 (d, J = 1.7 Hz, 3H), 1.33 (s, 12H). **^{13}C NMR (100.56 MHz, $CDCl_3$, 295 K):** [for major isomer] δ [ppm] = 142.5, 138.1, 129.5, 128.2, 127.2, 83.6, 25.0, 16.0. **^{11}B NMR (128.30 MHz, $CDCl_3$, 295 K):** δ [ppm] = 30.8. **HR-MS (EI$^+$):** $[M]^+$ = $C_{15}H_{21}BO_2^+$, calcd.: 244.16291, found: 244.16381. Note: This compound was also prepared following **GP2** (see Section 3.2).

(Z)-(4-Phenylbut-2-en-2-yl)boronic acid pinacol ester ((Z)-*int*-8b)

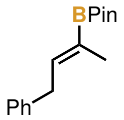

(Z)-*int*-**8b**

Known compound.[15] Employing **GP3**, reduction of alkyne **7b** yielded the hydroboration products *ter*-**8b**, (Z)-*int*-**8b** and (E)-*int*-**8b** in a ratio of 10:67:23. The products **8b** were obtained as isomeric mixture in 65 % yield. **^{1}H NMR (600.13 MHz, $CDCl_3$, 295 K):** for (Z)-*int*-**8b**: δ [ppm] = 7.30–7.25 (m, 2H), 7.22–7.16 (m, 3H), 6.49–6.45 (m, 1H), 3.48 (d, J = 7.1 Hz, 2H), 1.81 (s, 3H), 1.25 (s, 12H); for (E)-*int*-**8b**: δ [ppm] = 7.30–7.16 (m, 5H), 6.19 (t, J = 7.0 Hz, 1H), 3.70 (d, J = 7.8 Hz, 2H), 1.79 (s, 3H), 1.30 (s, 12H); for *ter*-**8b**: δ [ppm] = 7.30–7.16 (m, 5H), 5.79 (d, J = 3.2 Hz, 1H), 5.61 (s, 1H), 2.76–2.72 (m, 2H), 2.50–2.44 (m, 2H), 1.27 (s, 12H). **^{13}C NMR (150.90 MHz, $CDCl_3$, 295 K):** for (Z)-*int*-**8b**: δ [ppm] = 144.4, 140.6, 128.8, 128.6, 126.0, 83.3, 35.2, 24.9, 14.2. **^{11}B NMR (128.30 MHz, $CDCl_3$, 295 K):** δ [ppm] = 30.4. **HR-MS (EI$^+$):** $[M]^+$ = $C_{16}H_{23}BO_2^+$, calcd.: 258.17856, found: 258.18001.

(Z)-(4-((*tert*-Butyldimethylsilyl)oxy)but-2-en-2-yl)boronic acid pinacol ester ((Z)-*int*-8c)

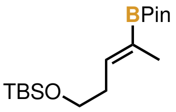

(Z)-*int*-**8c**

Known compound.[16] Employing **GP3**, reduction of alkyne **7c** yielded the hydroboration products *ter*-**8c**, (Z)-*int*-**8c** and (E)-*int*-**8c** in a ratio of 1:80:19. The products **8c** were obtained as isomeric mixture in 73 % yield. **^{1}H NMR (600.13 MHz, $CDCl_3$, 295 K):** for (Z)-*int*-**8c**: δ [ppm] = 6.29–6.26 (m, 1H), 3.65 (q, J = 7.4 Hz, 2H), 2.39–2.36 (m, 2H), 1.69 (s, 3H), 1.25 (s, 12H), 0.88 (s, 9H), 0.04 (s, 6H). **^{13}C NMR (150.90 MHz, $CDCl_3$, 295 K):** for (Z)-*int*-**8c**: δ [ppm] = 142.0, 83.3, 62.5, 32.7, 26.1, 24.9, 18.5, 14.1, −5.1. **^{11}B NMR (128.30 MHz, $CDCl_3$, 295 K):** δ [ppm] = 30.4. **HR-MS (EI$^+$):** $[M]^+$ = $C_{17}H_{35}BO_3Si^+$, calcd.: 326.24430, found: 326.24361.

(4-Cyanobut-2-en-2-yl)boronic acid pinacol ester (*int*-8d)

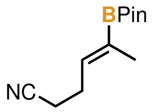

(*Z*)-*int*-**8d**

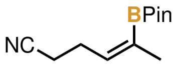

(*E*)-*int*-**8d**

Employing **GP3**, reduction of alkyne **7d** yielded the hydroboration products *ter*-**8d**, (*Z*)-*int*-**8d** and (*E*)-*int*-**8d** in a ratio of 5:62:33. Column chromatography (*n*-hexane/diethyl ether = 9:1) afforded the products **8d** as isomeric mixture in 67 % yield. Isomerically pure compounds (*Z*)-*int*-**8d** (colorless solid) and (*E*)-*int*-**8d** (colorless oil) could be isolated in 38 % and 16 % yield, respectively. **^{1}H NMR (600.13 MHz, $CDCl_3$, 295 K):** for (*Z*)-*int*-**8d**: δ [ppm] = 6.22–6.20 (m, 1H), 2.50–2.46 (m, 2H), 2.41–2.39 (m, 2H), 1.70 (s, 3H), 1.25 (s, 12H); for (*E*)-*int*-**8d** δ [ppm] = 6.06 (t, *J* = 7.2 Hz, 1H), 2.67 (q, *J* = 7.4 Hz, 2H), 2.37 (t, *J* = 7.3 Hz, 2H), 1.78 (s, 3H), 1.27 (s, 12H). **^{13}C NMR (150.90 MHz, $CDCl_3$, 295 K):** for (*Z*)-*int*-**8d**: δ [ppm] = 140.3, 119.6, 83.6, 24.9, 24.6, 16.8, 14.2; for (*Z*)-*int*-**8d**: δ [ppm] = 142.0, 119.8, 83.3, 26.8 25.0, 22.3, 18.0. **^{11}B NMR (128.30 MHz, $CDCl_3$, 295 K):** δ [ppm] = 29.9. **HR-MS (EI$^+$):** $[M]^+$ = $C_{12}H_{20}BNO_2^+$, calcd.: 221.15816, found: 221.15870.

2.2.4 Synthesis of a Bexaroten Precursor

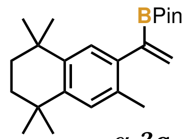

α-**3q**

Precatalyst **1a** (1.02 mg, 1.77 µmol, 0.5 mol%) was dissolved in 2 ml toluene and added to alkyne **2q** (80.1 mg, 353 µmol, 1.0 eq.). Subsequently, neat pinacolborane (102.6 µl, 707 µmol, 2.0 eq.) was added and the reaction mixture was stirred at room temperature for 18 h. After full conversion was detected by TLC, 2 ml of aqueous 2 M HCl were added and the mixture was extracted with dcm (3 × 2 ml). The combined organic phases were dried over $NaSO_4$ and purified by column chromatography (*n*-hexane/diethyl ether 49:1), affording the α-product as pale rose solid (58.1 mg, 164 µmol, 46 %). **^{1}H NMR (399.89 MHz, $CDCl_3$, 295 K):** δ [ppm] = 7.03 (s, 1H), 7.02 (s, 1H), 6.12 (d, *J* = 3.7 Hz, 1H), 5.81 (d, *J* = 3.6 Hz, 1H), 2.23 (s, 3H), 1.65 (s, 4H), 1.30 (s, 12H), 1.26 (s, 12H). **^{13}C NMR (100.56 MHz, $CDCl_3$, 295 K):** δ [ppm] = 143.3, 142,1, 139.6, 132.9, 131.9, 127.8, 126.8, 83.8, 35.4, 34.01, 34.00, 32.05, 32.02, 24.9, 20.2. **^{11}B NMR (128.30 MHz, $CDCl_3$, 295 K):** δ [ppm] = 30.3. **HR-MS (EI$^+$):** $[M]^+$ = $C_{23}H_{35}BO_2^+$, calcd.: 354.27246, found: 354.27309. Spectral properties are in accordance with reported data.[17]

3 Mechanistic Experiments

3.1 Precatalyst Activation

Reaction with HBPin

An equimolar mixture of (tmeda)Co(CH_2SiMe_3)$_2$ (4.0 mg, 11 µmol, 1.0 eq.) and ^{Ph}boxmi-H (5.0 mg, 11 µmol, 1.0 eq) was dissolved in 600 µl C_6D_6 and pinacolboran (3.3 µl, 22 µmol, 2.0 eq.) was added after 6 h. The reaction mixture was then monitored by ^1H, ^{11}B and ^{29}Si NMR spectroscopy, revealing the formation of Me_3SiCH_2BPin. A graphical illustration of the NMR spectra obtained is presented below (Figure S2).

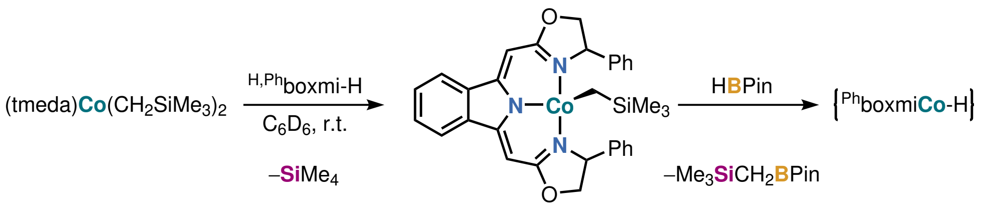

1a

In a separate experiment, the formation of [^{iPr}boxmiCo]$_2$ (**5**) upon addition of pinacolboran to precatalyst **1b** was observed (see Section 2.1).

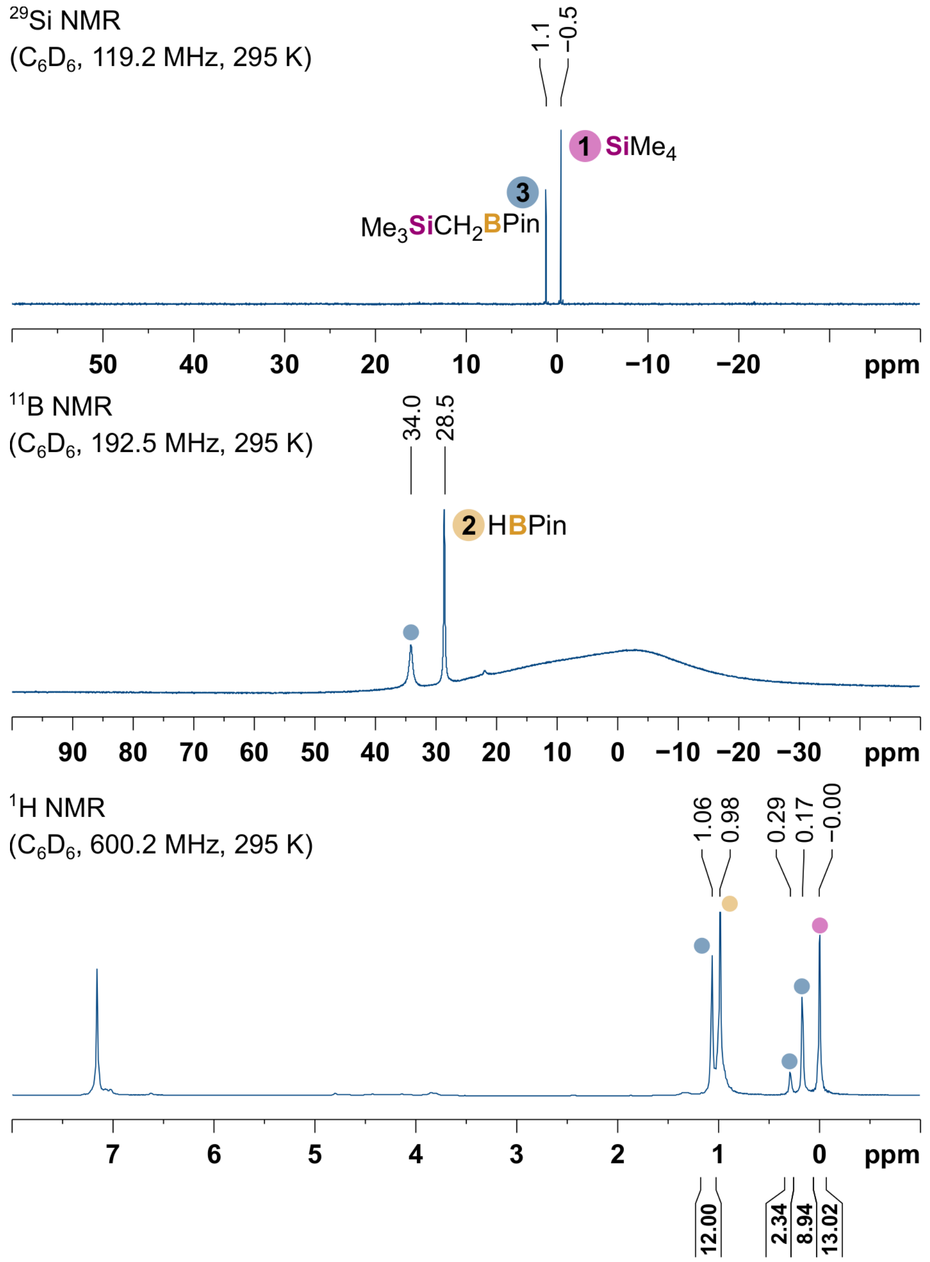

Figure S2: NMR spectra for the reaction of precatalyst **1a** with pinacolborane.

3.2 Hydroboration of an Internal Alkyne

The hydroboration of 1-phenyl-1-propyne (**2p**) was conducted on a 300 µmol scale following **GP2**. The mixture of four isomers was purified by column chromatography (*n*-hexane/diethyl ether 39:1), affording the two (*Z*)-isomers in ratio α:β = 1:2.5 and 56 % yield. The share of each (*E*)-isomer is lower than 5%. Isomers were assigned based on ^{1}H NMR spectroscopy by comparison to literature data.[14]

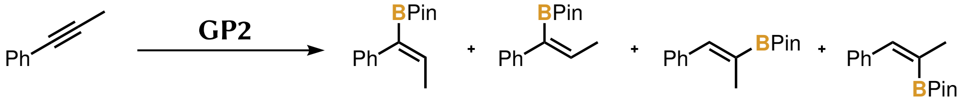

2p 56 % yield (*Z*)-α-**3p** 26 % (*E*)-α-**3p** <5 % (*Z*)-β-**3p** 65 % (*E*)-β-**3p** <5 %

^{1}H NMR (600.13 MHz, $CDCl_3$, 295 K): for (*Z*)-α-**3p**: δ [ppm] = 7.33–7.30 (m, 2H), 7.26–7.23 (m, 3H), 6.72 (q, *J* = 6.9 Hz, 1H), 1.77 (d, *J* = 6.8 Hz, 3H), 1.27 (s, 12H); for (*Z*)-β-**3p**: δ [ppm] = 7.40–7.38 (m, 2H), 7.36–7.33 (m, 2H), 7.21 (m, 1H), 7.16 (m, 1H), 1.99 (d, *J* = 1.6 Hz, 3H), 1.31 (s, 12H). **^{13}C NMR (100.56 MHz, $CDCl_3$, 295 K):** for both (*Z*)-α-**3p** and (*Z*)-β-**3p**: δ [ppm] = 142.8, 142.5, 139.9, 138.0, 129.5, 129.2, 128.2, 127.9, 127.2, 126.0, 83.6, 83.5, 25.0, 24.9, 16.1, 16.0. **^{11}B NMR (128.30 MHz, $CDCl_3$, 295 K):** δ [ppm] = 30.8.

The isomer ratio was determined by ^{1}H NMR spectroscopy as illustrated below (Figure S3). For a full spectrum, see Section 6.

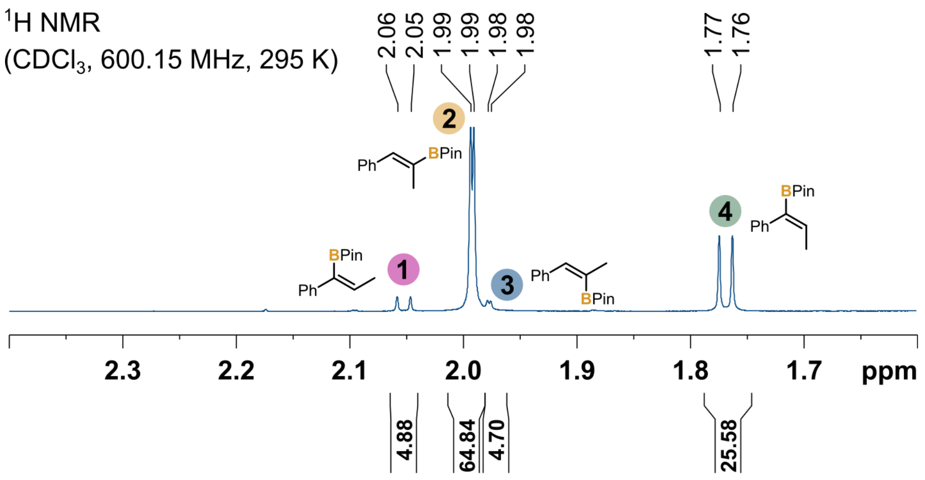

Figure S3: Excerpt of the ^{1}H NMR spectrum of isolated **9a** for the determination of isomer ratios.

3.3 Deuterium Labeling Studies

3.3.1 Evaluation of the H/D Distribution

To a solution of phenylacetylene (**2b**, 10.0 mg, 97.9 µmol, 1.0 eq.) and precatalyst **1a** (1.4 mg, 2.5 µmol, 2.5 mol%) in 600 µl C_6D_6 was added pinacolborane (15.6 µl, 108 µmol, 1.1 eq). The reaction progress was then monitored by ^{1}H NMR spectroscopy. After approximately 20 h, the reaction mixture was filtered through a plug of silica and eluted with 4 ml dichloromethane. The solvent was removed *in vacuo* and the residue was analyzed by ^{1}H NMR spectroscopy.

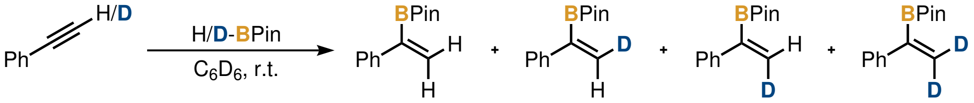

2b H,H-α-**3b** (*E*)-H,D-α-**3b** (*Z*)-H,D-α-**3b** D,D-α-**3b**

This procedure was performed twice with one deuterated reagent each, *i.e.* reacting phenylacetylene with DBPin and treating phenylacetylene-*d* with HBPin. The respective signals attributed to one proton position were completely integrated and signal deconvolution as implemented in Bruker TopSpin 3.0 was carried out in order to determine the specific contributions. The H/D distributions in the α-borylated product are summarized in Table S2. A representative ^{1}H NMR spectrum illustrating the deuterium incorporation can be found below (Figure S4). DBPin was prepared according to a literature procedure.[18]

Table S2: H/D distribution in α-borylated product α-**3b** for the cobalt-catalyzed hydroboration of phenylacetylene.

product	PhCCD	DBPin
H,H-α-**3b**	30 %	23 %
(*E*)-H,D-α-**3b**	24 %	24 %
(*Z*)-H,D-α-**3b**	25 %	25 %
D,D-α-**3b**	21 %	28 %
Total *D*	91 %	105 %

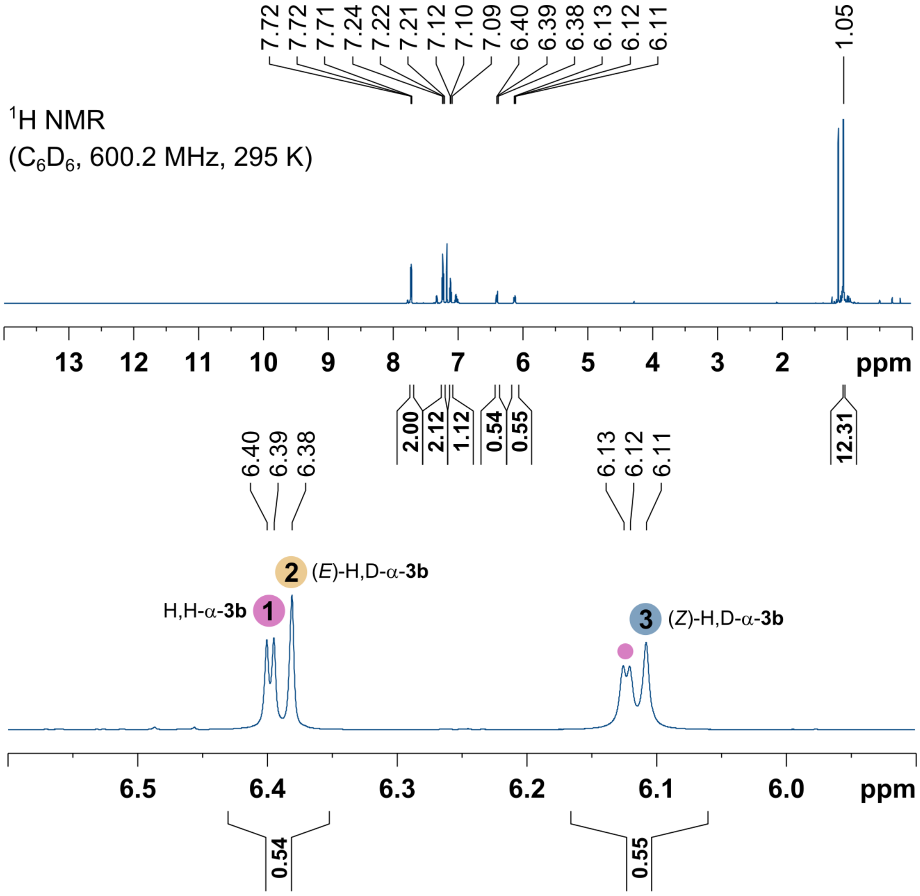

Figure S4: Representative ^{1}H NMR spectrum obtained from a deuterium labeling experiment and an excerpt for the evaluation of the H/D-distribution.

3.3.2 Evidence for H/D Exchange

To a solution of phenylacetylene-*d* (**2b**, 10.0 mg, 97.0 µmol, 1.0 eq.) and precatalyst **1a** (1.4 mg, 2.4 µmol, 2.5 mol%) in 600 µl C_6D_6 was added pinacolborane (15.5 µl, 107 µmol, 1.1 eq). The reaction progress was then monitored by ^{1}H NMR spectroscopy. After 5 h (approximately 85% conversion), the reaction mixture was filtered through a short plug of silica, eluted with 1 ml C_6D_6 and analyzed by ^{1}H NMR spectroscopy (Figure S5). The deuteration degree of the residual alkyne decreased from >99% in the starting material to approximately 95%, indicating the occurrence of H/D exchange at the alkyne. This phenomenon was not observed in the absence of the cobalt catalyst.

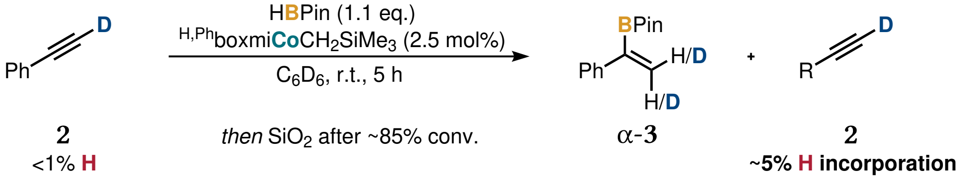

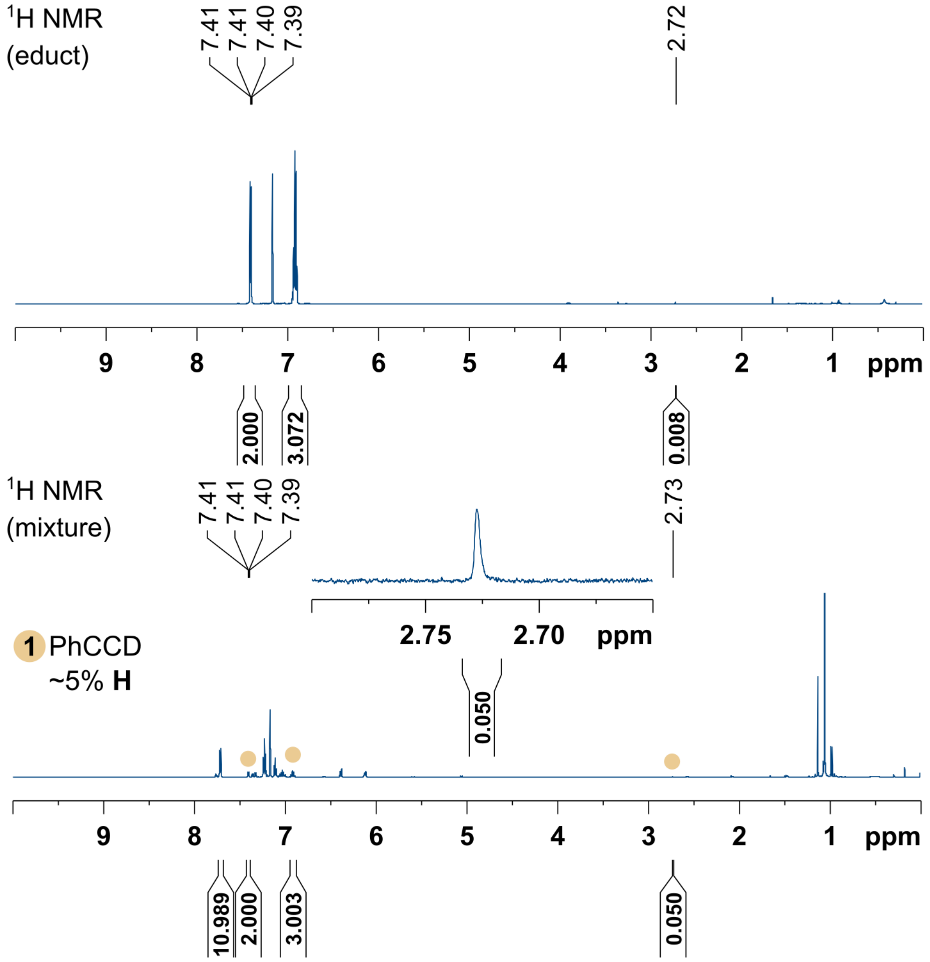

Figure S5: ^{1}H NMR spectra (C_6D_6, 600.2 MHz, 295 K) of phenylacetylene-*d* (*top*) and the reaction mixture (*bottom*), illustrating a significant H incorporation in the starting material.

3.4 Conversion of ^{iPr}boxmiCo(C(Me)C(H)Ph) (4)

Reaction with HBPin

Vinyl Complex **4** (7.8 mg, 14 µmol, 1.0 eq.) was dissolved in 600 µl C_6D_6 and neat pinacolboran (2.1 µl, 14 µmol, 1.0 eq.) was added. The reaction mixture was then monitored by ^{1}H and ^{11}B NMR spectroscopy. After 4 h, the reaction mixture was filtered through a plug of silica and eluted with dichloromethane. After removing all volatiles *in vacuo*, the residue was analyzed by ^{1}H NMR spectroscopy in CD_2Cl_2, showing the sole presence of (*Z*)-β-**3p** (Figure S6).

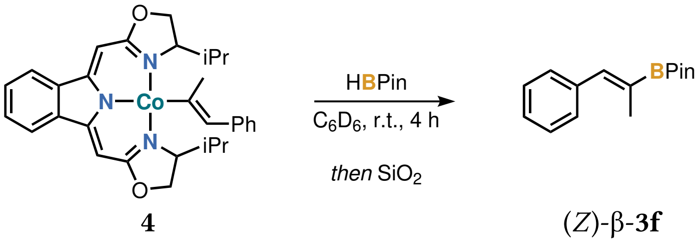

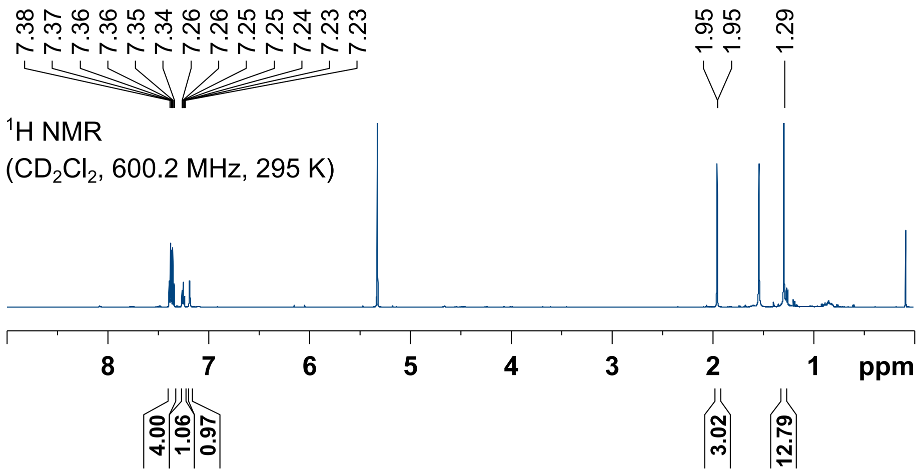

Figure S6: ^{1}H NMR spectrum after work-up for the reaction of complex **4** with pinacolborane.

3.5 Conversion of $[^{iPr}boxmiCo]_2$ (5)

Reaction with HBPin

A J. Young NMR tube was charged with cobalt(I) complex **5** (7.9 mg, 9.3 µmol, 0.5 eq.) and a solution of pinacolborane (2.7 µl, 19 µmol, 1.0 eq.) in 600 µl C_6D_6. The reaction progress was then followed by ^1H and ^{11}B NMR spectroscopy. Representative NMR spectra after 2 h are depicted below, indicating no depletion of $[^{iPr}boxmiCo]_2$ and the slow emergence of B_2Pin_3 (Figure S7).[19]

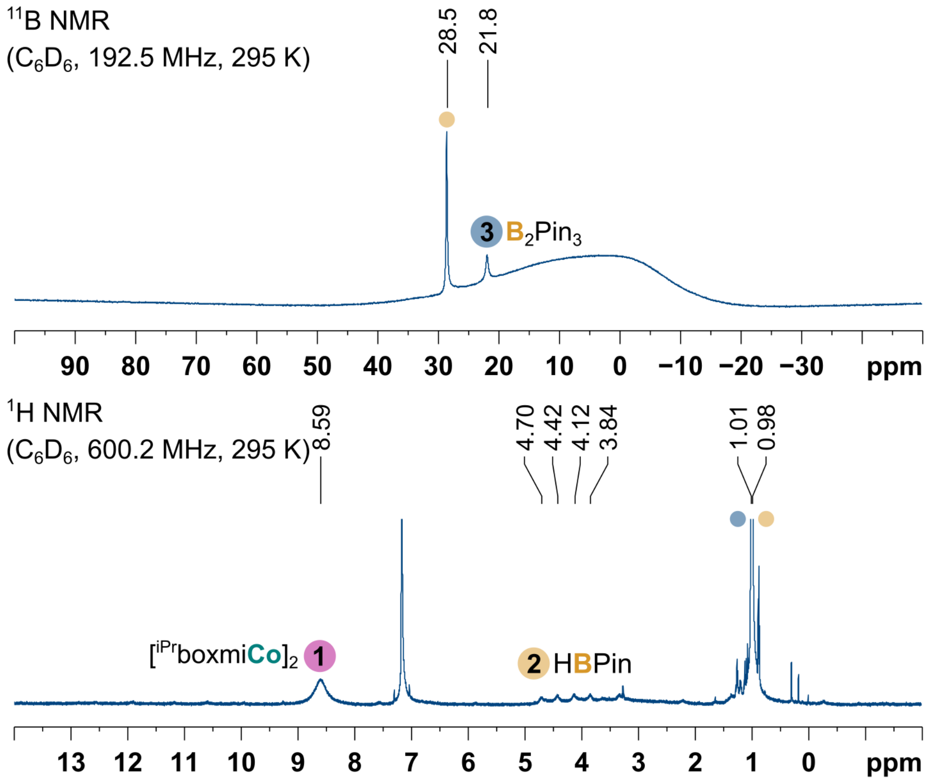

Figure S7: *In situ* NMR spectra for the reaction of cobalt(I) complex **5** with pinacolborane.

Reaction with an Internal Alkyne and Pinacolborane

Cobalt(I) complex $[^{iPr}boxmiCo]_2$ (**5**, 8.01 mg, 9.46 µmol, 0.5 eq.) was dissolved in 600 µl C_6D_6 and simultaneously treated with 1-phenyl-1-propyne (**2p**, 2.37 µl, 18.9 µmol, 1.0 eq.) and pinacolborane (2.75 µl, 18.9 µmol, 1.0 eq.). The mixture was then transferred into a J. Young NMR tube and the reaction progress was followed by ^{1}H and ^{11}B NMR spectroscopy. Representative NMR spectra after 15 min reaction time are depicted below, indicating the depletion of complex **5** and the formation of vinyl complex **4** as major product (Figure S8).

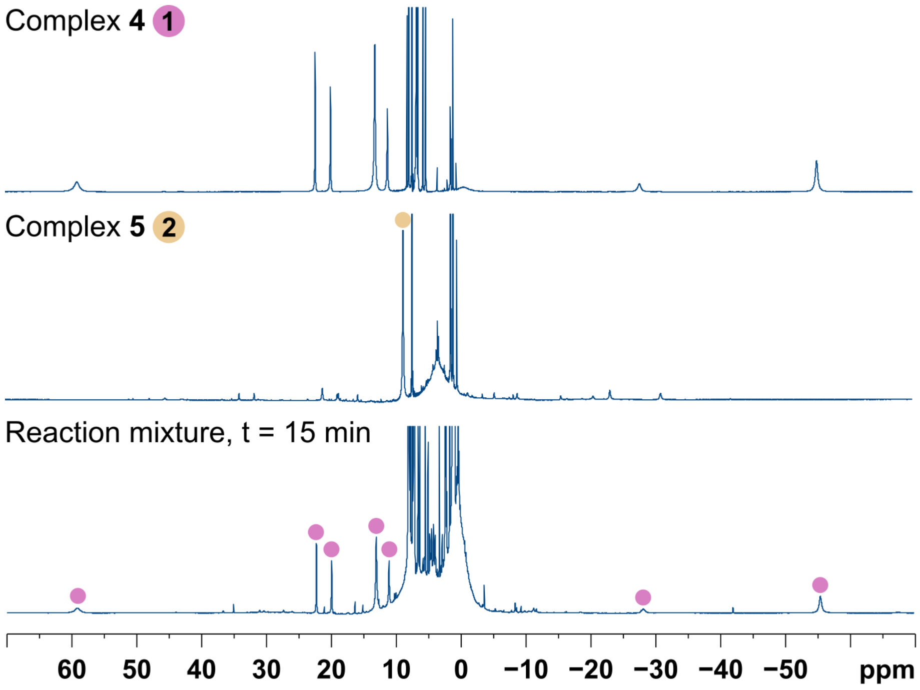

Figure S8: *In situ* ^{1}H NMR (C_6D_6, 600.2 MHz, 295 K) spectrum for the reaction of cobalt(I) complex **5** with 1-phenyl-1-propyne after 15 min and reference spectra for isolated vinyl complex **4** and cobalt(I) complex **5**.

3.6 Kinetic Profiles

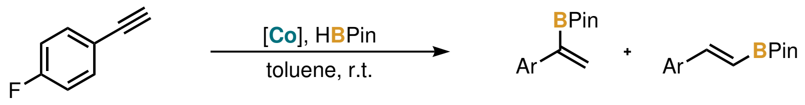

2a α-**3a** β-**3a**

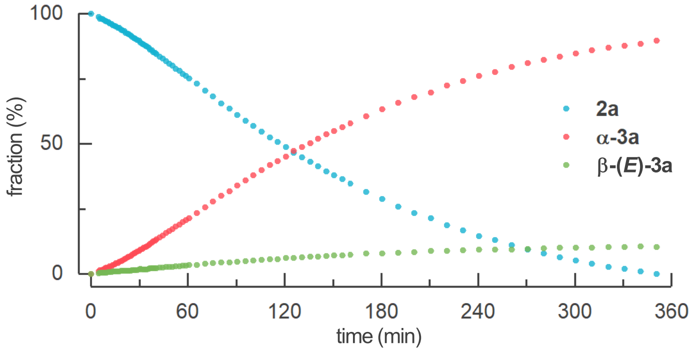

Figure S9: Reaction profile for the cobalt-catalyzed hydroboration with ^{Ph}boxmiCo(CH_2SiMe_3) (**1a**) monitored by ^{19}F NMR spectroscopy; fraction in reaction mixture of **2a** (blue), α-**3a** (red) and β-**3a** (green) given. Conditions: $[Alkyne]_0/M = 0.22$, $[HBPin]_0/M = 0.33$, $[Co]_0/M$ 0.005, room temperature, toluene.

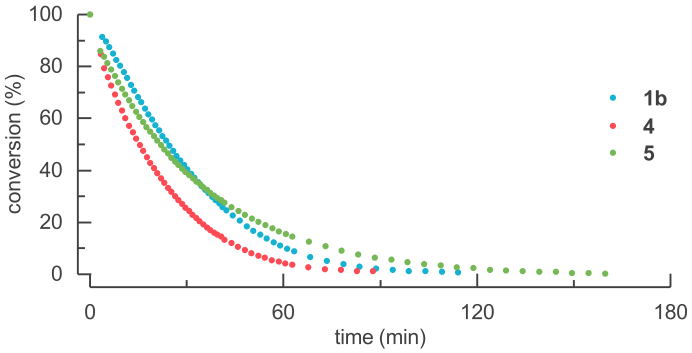

Figure S10: Reaction profile for the cobalt-catalyzed hydroboration with different catalysts monitored by ^{19}F NMR spectroscopy; conversion of alkyne given. Conditions: (a) ^{iPr}boxmiCo(CH_2SiMe_3), blue: $[Alkyne]_0/M = 0.21$, $[HBPin]_0/M = 0.33$, $[\mathbf{1b}]_0/mM = 5.2$; (b) ^{iPr}boxmiCo(C(Me)C(H)Ph), red: $[Alkyne]_0/M = 0.22$, $[HBPin]_0/M = 0.34$, $[\mathbf{4}]_0/mM = 5.1$; (c) [^{iPr}boxmiCo]$_2$, green: $[Alkyne]_0/M = 0.21$, $[HBPin]_0/M = 0.32$, $[\mathbf{5}]_0/mM = 2.6$.

4 X-ray Crystal Structure Determinations

Crystal data and details of the structure determinations are compiled in Table S3. Full shells of intensity data were collected at low temperature with an Agilent Technologies Supernova-E CCD diffractometer (Mo- or Cu-K_α radiation, microfocus X-ray tubes, multilayer mirror optics). Detector frames (typically ω-, occasionally φ-scans, scan width 0.4...1°) were integrated by profile fitting.[20] Data were corrected for air and detector absorption, Lorentz and polarization effects[21] and scaled essentially by application of appropriate spherical harmonic functions.[21,22,23] Absorption by the crystal was treated numerically (Gaussian grid).[22,24] An illumination correction was performed as part of the numerical absorption correction.[22]

The structures were solved by the charge flip procedure (compound **1b**)[25] or by ab initio dual space methods involving difference Fourier syntheses (VLD procedure)[26,27] and refined by full-matrix least squares methods based on F^2 against all unique reflections.[28] All non-hydrogen atoms were given anisotropic displacement parameters. Hydrogen atoms were generally input at calculated positions and refined with a riding model.[29] The positions of some hydrogen atoms in the structure of **5** were taken from difference Fourier syntheses and refined.

Crystals of **4** · 0.5 *n*-hexane were twinned; the structure was solved using only reflections with small overlap factors (typically < 0.4). Final refinement was carried out against all single and composite observations involving the major domain only. Suitable geometry and adp restraints were applied to the solvent molecules.[29,30]

CCDC 2014620–2014622 contains the supplementary crystallographic data for this paper. These data can be obtained free of charge from the Cambridge Crystallographic Data Centre's and FIZ Karlsruhe's joint Access Service *via* https://www.ccdc.cam.ac.uk/structures/?.

Table S3: Details of the crystal structure determinations of compounds **1b**, **4** and **5**.

Compound	**1b**	**4** · 0.5 *n*-hexane	**5**
Deposition number, CCDC	2014620	2014621	2014622
Empirical formula	$C_{26}H_{37}CoN_3O_2Si$	$C_{34}H_{42}CoN_3O_2$	$C_{44}H_{52}Co_2N_6O_4$
Formula weight M_r /g·mol^{-1}	510.60	583.63	846.77
Crystal system	monoclinic	monoclinic	triclinic
Space group	$P2_1$ (*IT* Nr. 4)	$P2_1$ (*IT* Nr. 4)	$P1$ (*IT* Nr. 1)
a /Å	10.5086(3)	11.09818(17)	8.44516(8)
b /Å	14.5982(3)	20.5961(4)	11.36855(9)
c /Å	16.7986(5)	26.0382(5)	11.37499(8)
α /°			115.4679(7)
β /°	90.009(3)	94.5148(16)	98.5568(7)
γ /°			92.9519(8)
V /Å^3	2577.02(12)	5933.32(19)	966.814(15)
Z	4	8	1
F_{000}	1084	2480	444
d_c /Mg·m^{-3}	1.316	1.307	1.454
X-radiation, λ /Å	Cu-K_α, 1.54184	Cu-K_α, 1.54184	Mo-$K\alpha$, 0.71073
μ/mm^{-1}	5.876	4.802	0.911
Transmission factors: max, min	0.702, 0.602	1.000, 0.431	1.000, 0.583
Data collect. temp. /K	120(1)	120(1)	120(1)
θ range for data collection /°	4.0 to 70.9	2.7 to 71.8	2.5 to 34.1
Index ranges h, k, l	−12...12, −17...17, −20...20	−13...13, −25...25, −31...31	−13...13, −17...17, −17...17
Reflections measured	99886	205393	93441
Independent refl. [R_{int}]	9724 [0.0844]	33358 [0.1027]	15242 [0.0999]
Observed refl. [$I \geq 2\sigma(I)$]	8392	17839	13688
Data / restraints / parameters	9724 / 1 / 609	33358 / 188 / 1462	15242 / 3 / 525
GooF on F^2	1.053	0.820	1.064
R [$F > 4\sigma(F)$], $R(F)$, $\omega R(F^2)$	0.0605, 0.1564	0.0521, 0.0893	0.0465, 0.1081
R (all data) $R(F)$, $\omega R(F^2)$	0.0720, 0.1651	0.1040, 0.1000	0.0517, 0.1127
Absolute structure parameter	−0.029(3)	−0.028(2)	−0.028(8)
Diff. density: rms, max, min /e·Å^3	0.085, 1.379, −0.574	0.052, 0.431, −0.379	0.103, 1.461, −1.187

5 Spectroscopic Data

5.1 Temperature-Dependent Analysis of [^{iPr}boxmiCo]$_2$ (5)

VT Evans Measurements

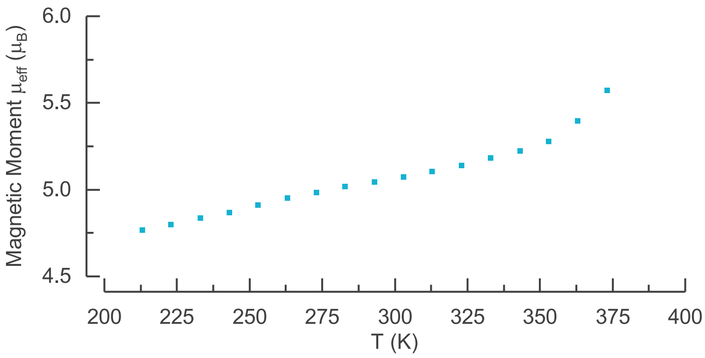

Figure S11: Evans measurements (Tol-d_8) of cobalt(I) complex **5** at various temperatures calculated as dimer.[31]

VT NMR Studies

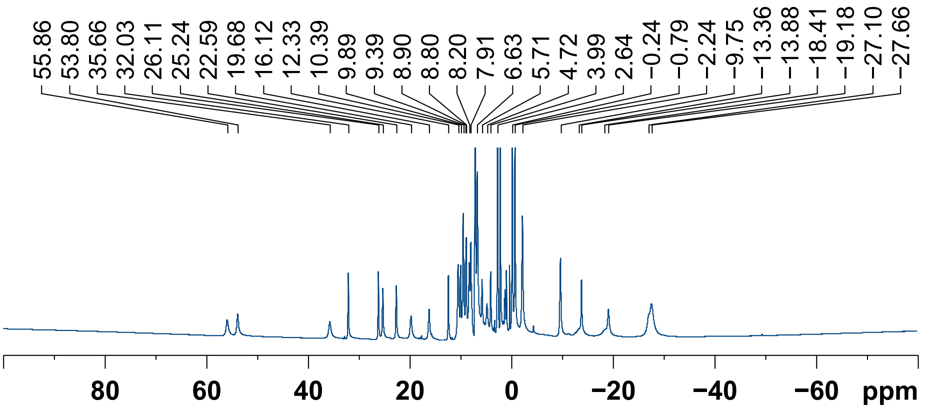

Figure S12: ^{1}H NMR spectrum (Tol-d_8, 399.9 MHz) of cobalt(I) complex **5** at 235 K.

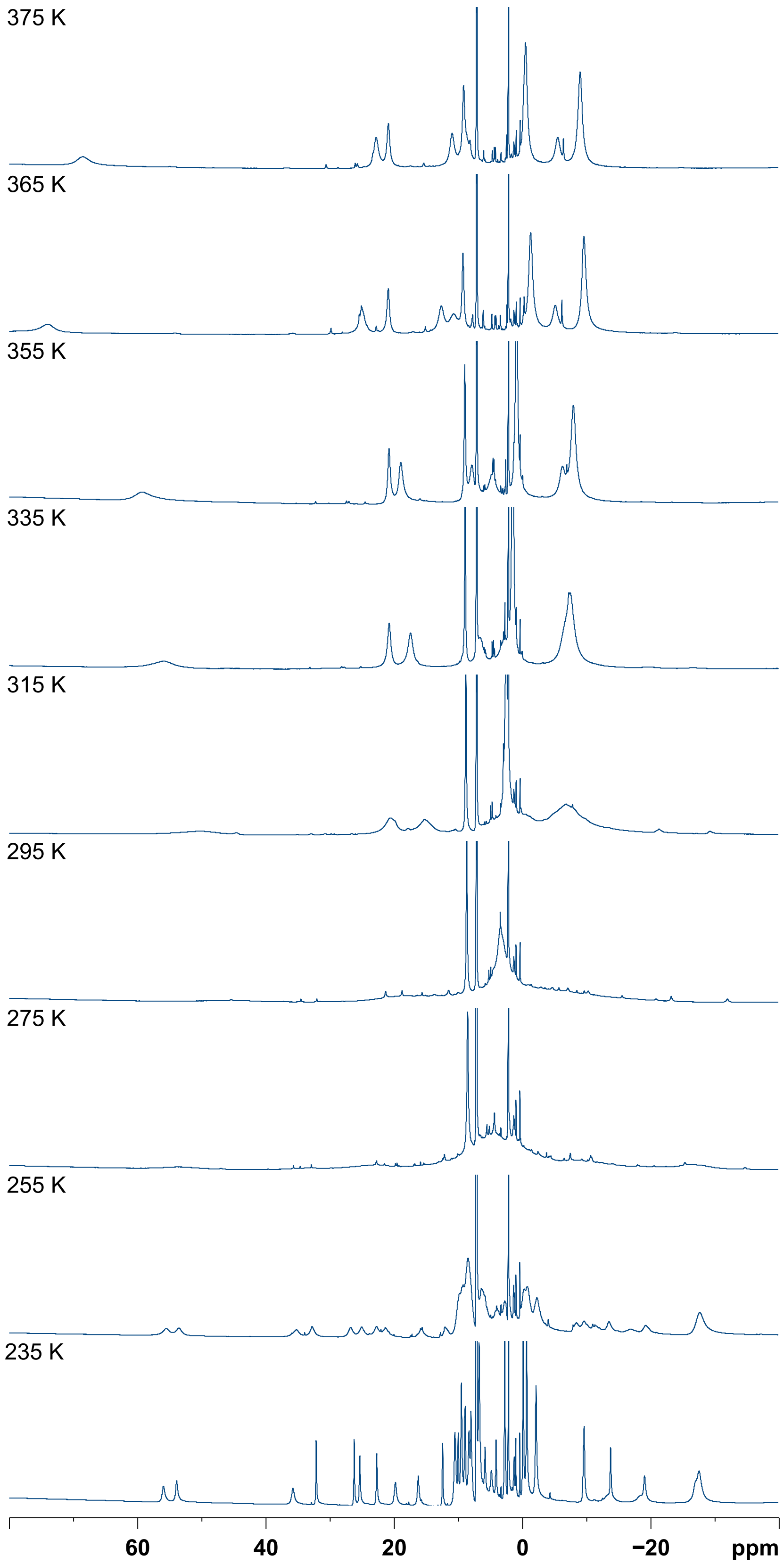

Figure S13: ^{1}H NMR (Tol-d_8, 399.9 MHz) spectra of cobalt(I) complex **5** at variable temperatures.

5.2 Squid Magnetometry

SQUID measurements were conducted on a Quantum Design MPMS XL SQUID magnetometer, employing polycarbonate capsules (Quantum Designs, QDC-AGC3). The solid compound was powdered (*agate mortar*) prior to transfer into the capsule. The loaded capsule was kept in an Argon filled Schlenk flask and was then quickly transferred to the magnetometer, which was promptly evacuated. The significant amount of paramagnetic impurities found for compound **5** might be attributed to its exceptional sensitivity towards oxygen and moisture, resulting in the formation of mononuclear reaction products during capsule transfer. The background data measured for an empty capsule and the diamagnetic contribution calculated from Pascal constants were subtracted from the experimental data. Data fitting was performed with the software package PHI.[32]

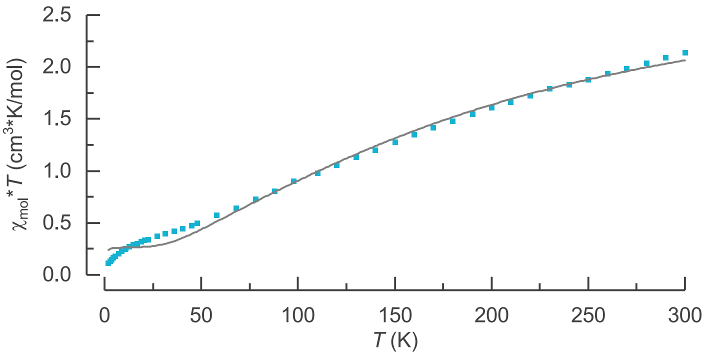

Figure S14: Variable temperature magnetic susceptibility data for [^{iPr}boxmiCo]$_2$ (**5**, external magnetic field of 1.0 T, temperature range 2 K to 290 K, experimental data as blue dots, fitted data as grey line). Parameters for fit: Two coupled S = 1 centers, 26 % S = 1 impurity, TIP = $5 \cdot 10^{-4}$ cm^3/mol, g = 2.67, J = 54 cm^{-1}.

6 NMR Data

Compound 4

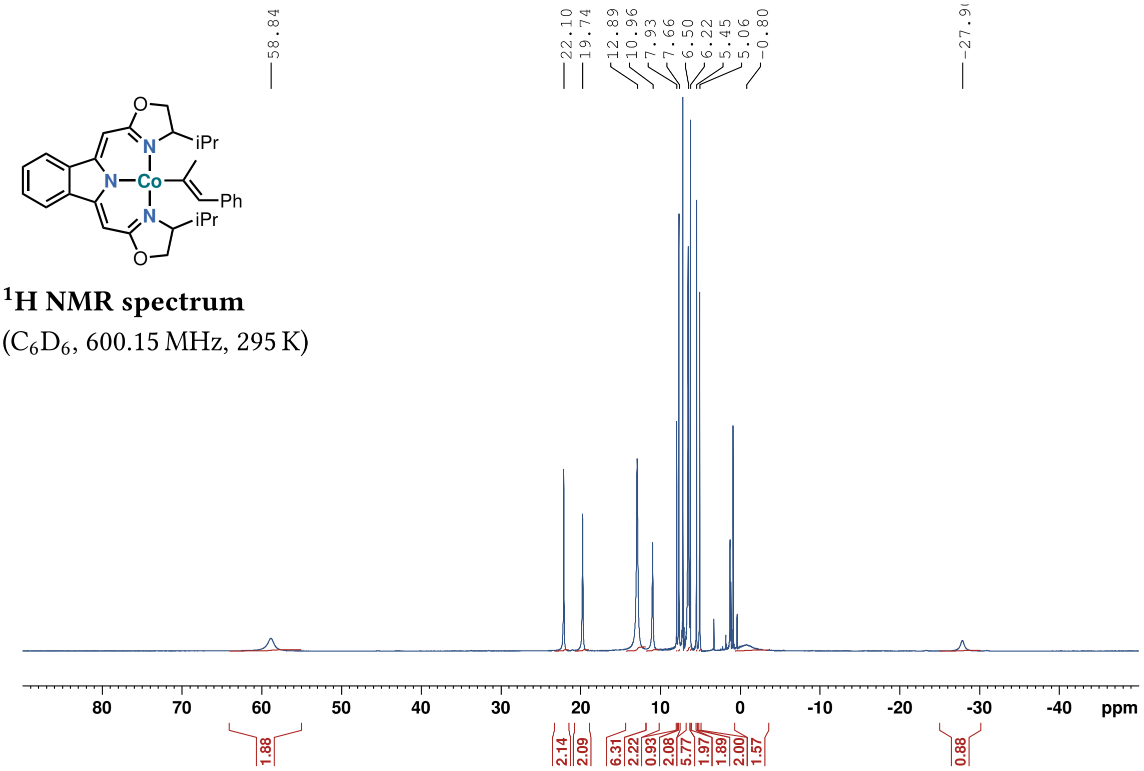

^{1}H NMR spectrum
(C_6D_6, 600.15 MHz, 295 K)

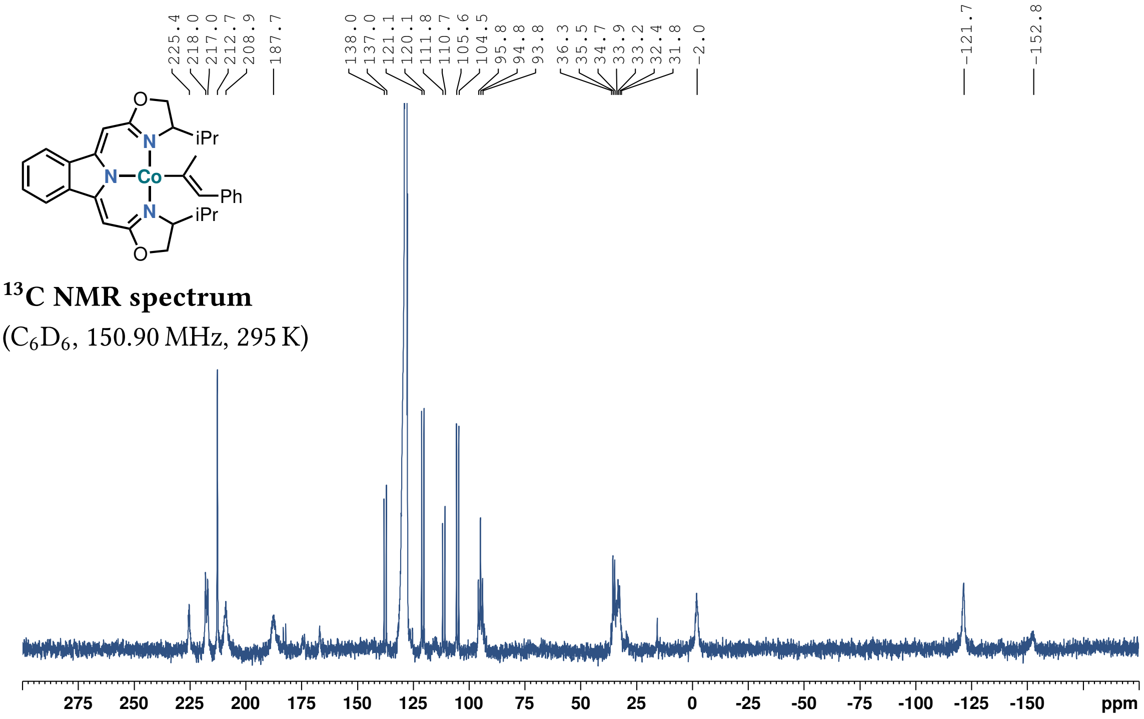

^{13}C NMR spectrum
(C_6D_6, 150.90 MHz, 295 K)

Compound α-3b

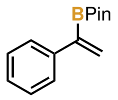

α-**3b**

^{1}H NMR spectrum
($CDCl_3$, 399.89 MHz, 295 K)

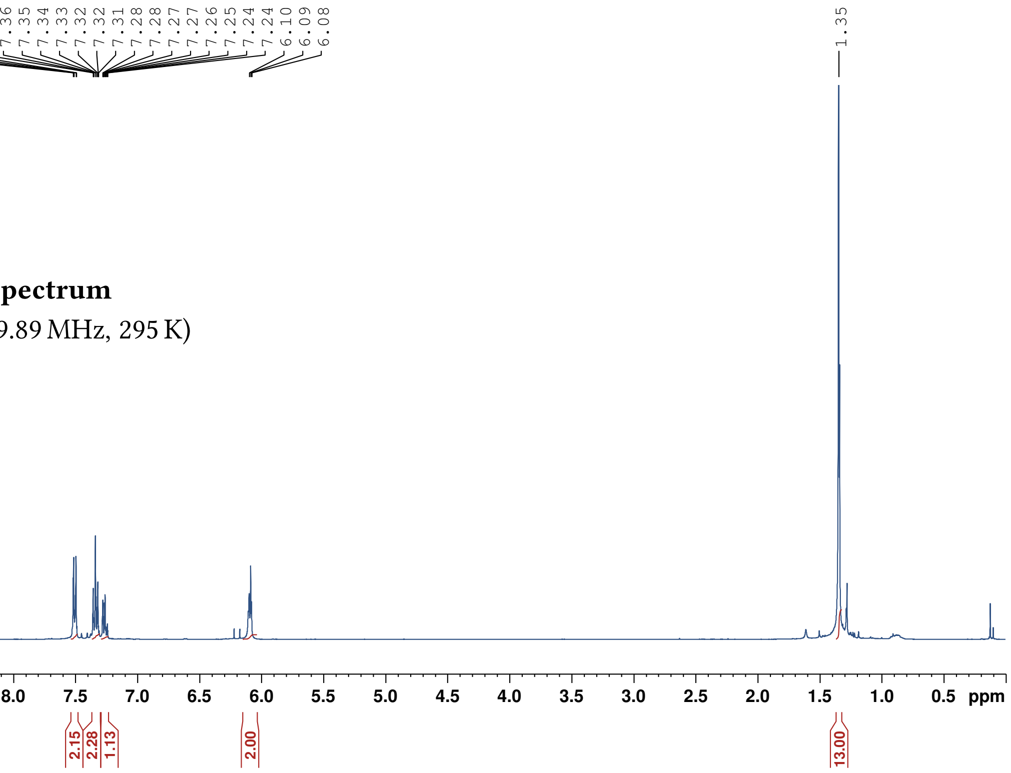

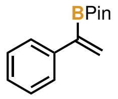

α-**3b**

^{13}C NMR spectrum
($CDCl_3$, 100.55 MHz, 295 K)

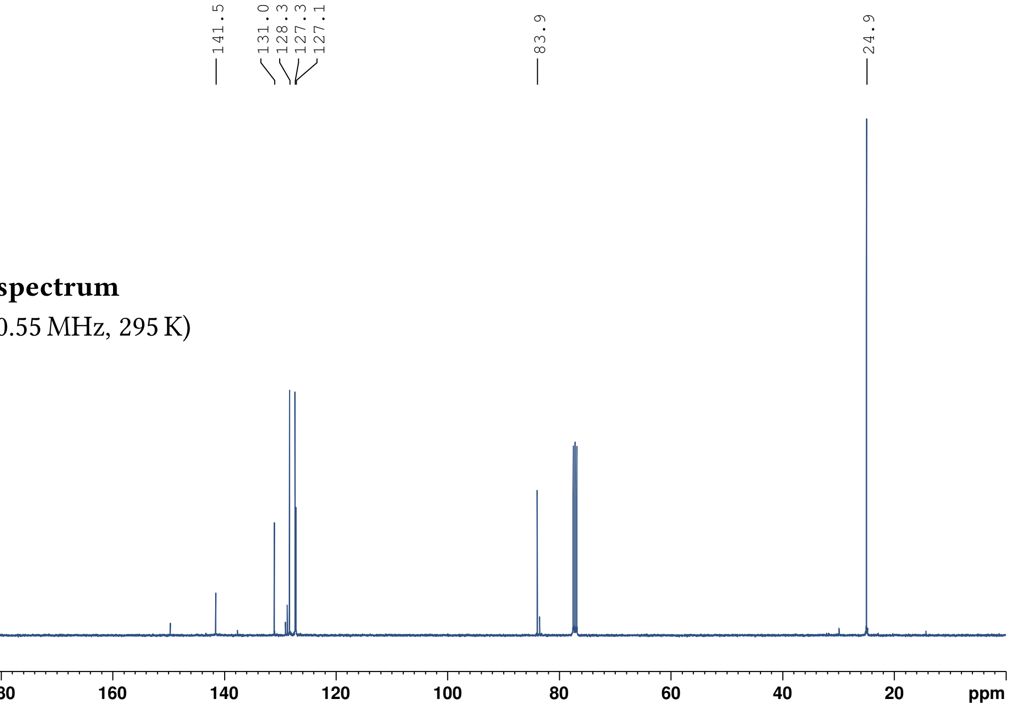

Compound α-3a

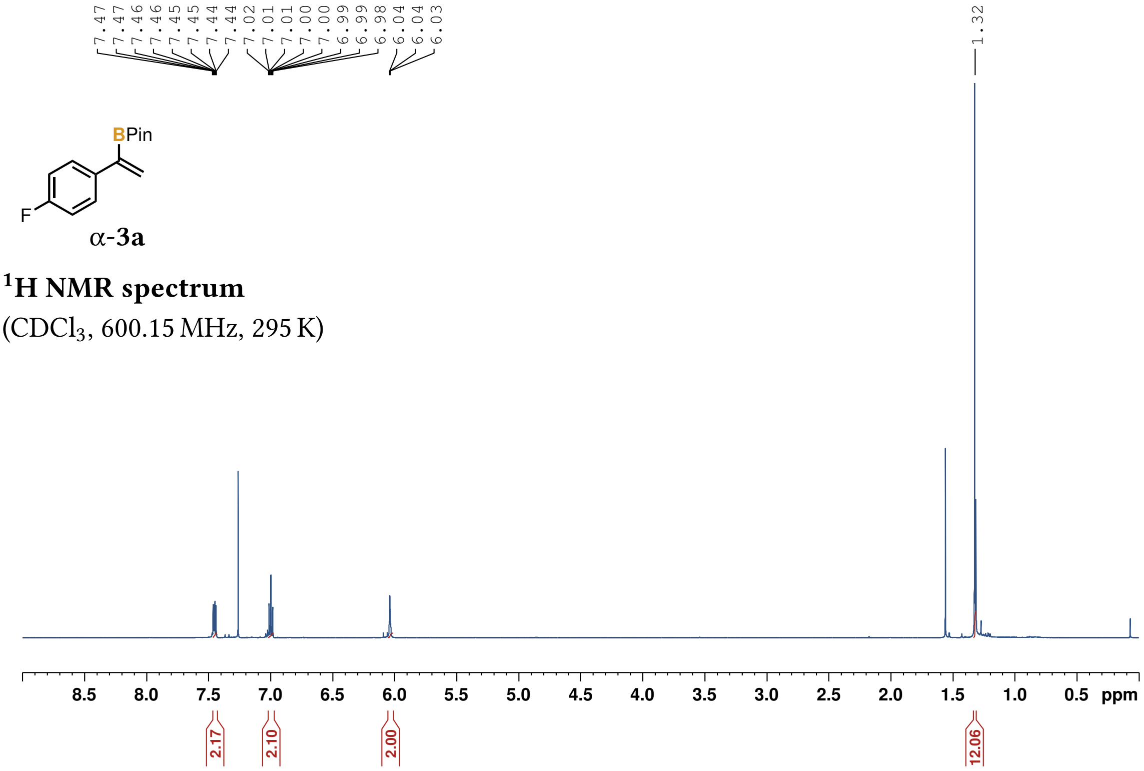

^{1}H NMR spectrum
($CDCl_3$, 600.15 MHz, 295 K)

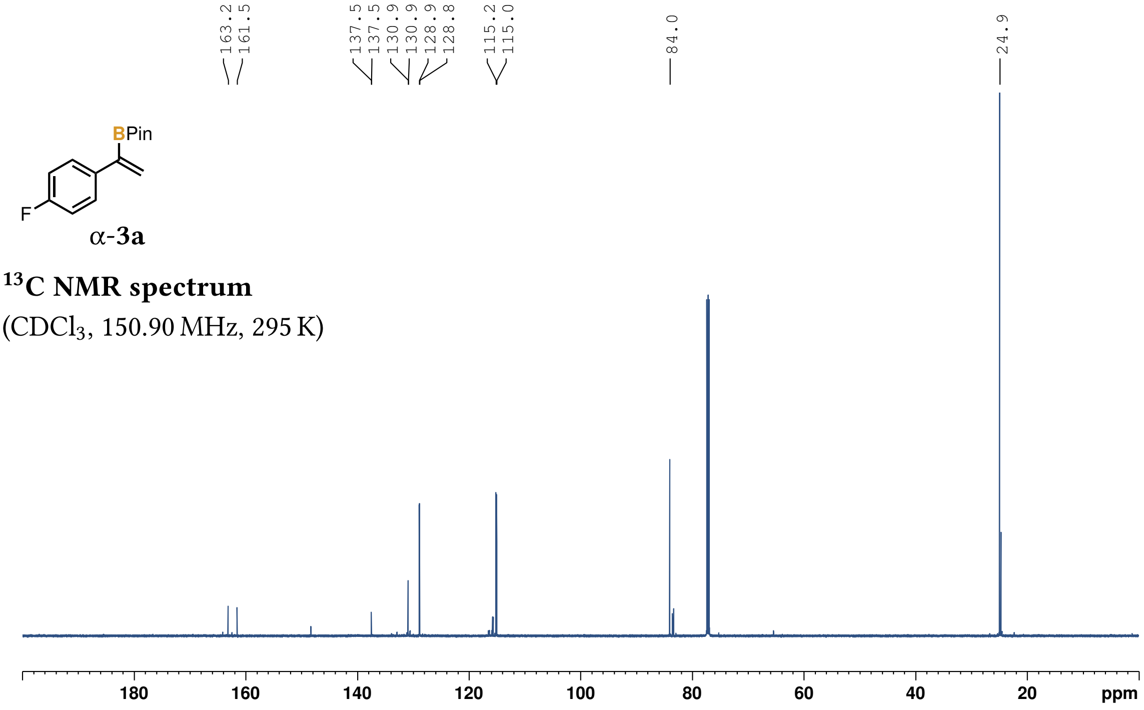

^{13}C NMR spectrum
($CDCl_3$, 150.90 MHz, 295 K)

Compound α-3c

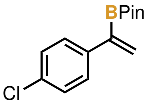

α-3c

^{1}H NMR spectrum

($CDCl_3$, 399.89 MHz, 295 K)

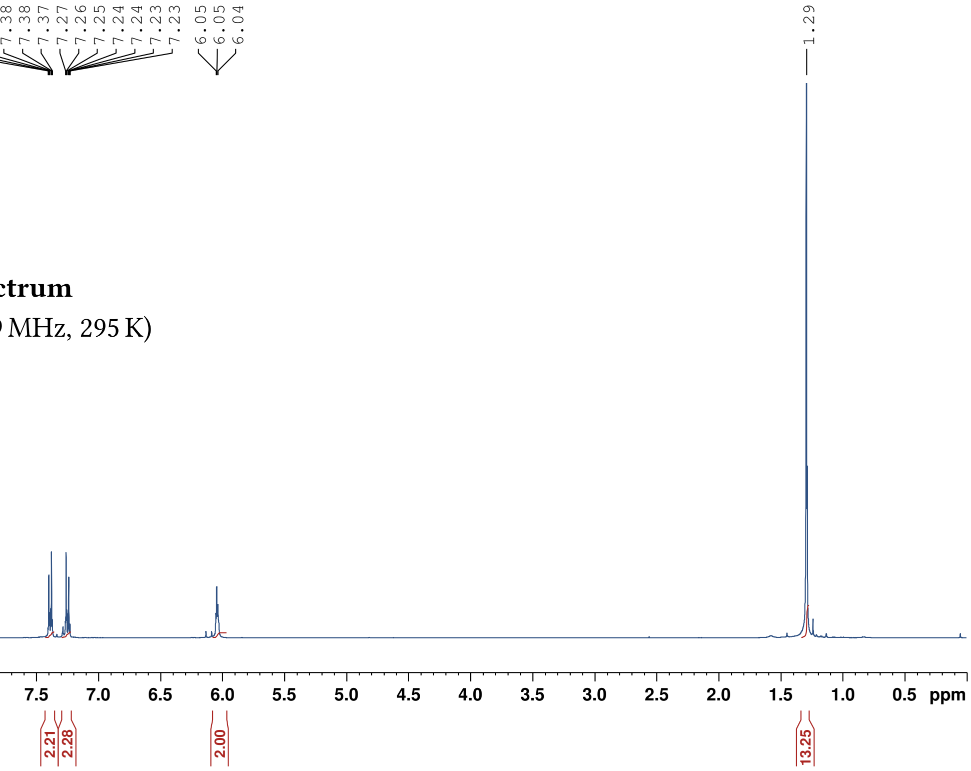

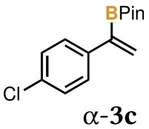

α-3c

^{13}C NMR spectrum

($CDCl_3$, 100.55 MHz, 295 K)

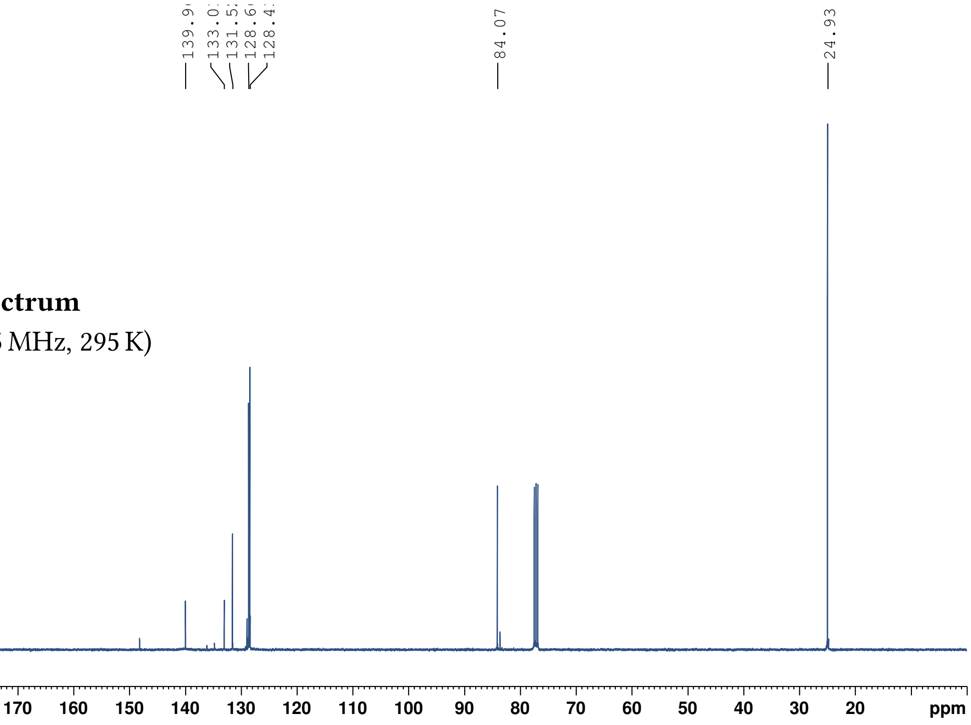

Compound α-3d

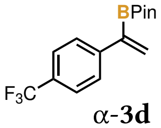

α-**3d**

^{1}H NMR spectrum

($CDCl_3$, 399.89 MHz, 295 K)

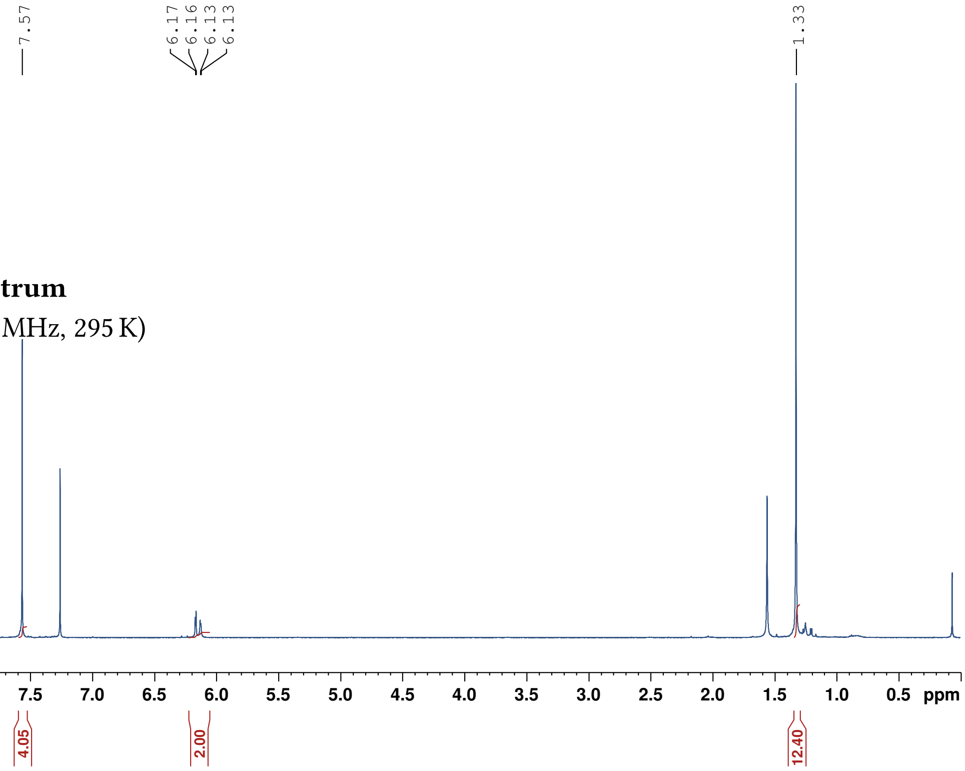

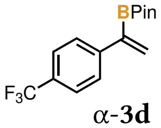

α-**3d**

^{13}C NMR spectrum

($CDCl_3$, 100.55 MHz, 295 K)

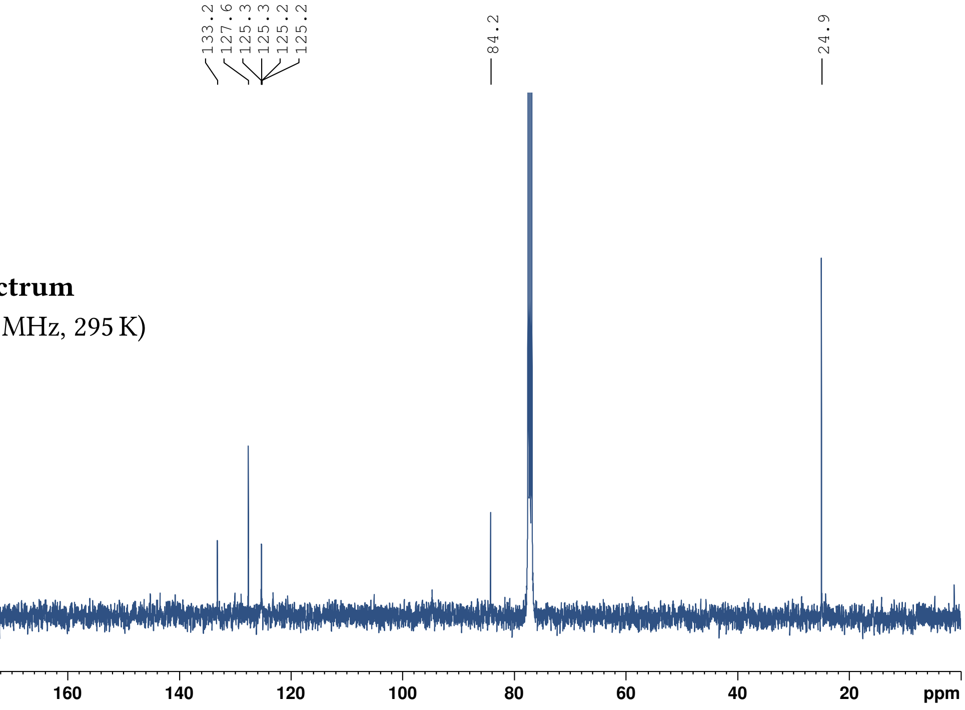

Compound α-3e

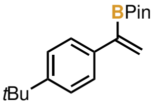

α-**3e**

^{1}H NMR spectrum

($CDCl_3$, 600.15 MHz, 295 K)

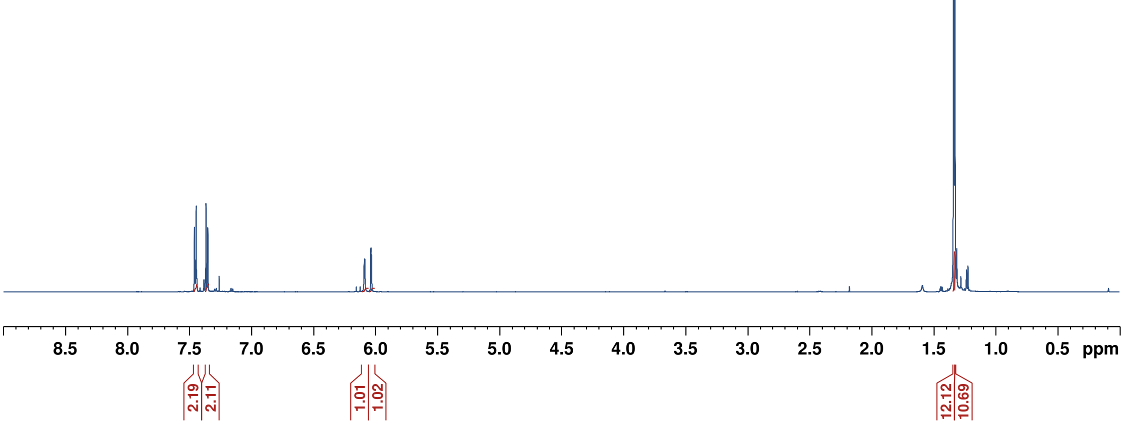

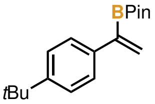

α-**3e**

^{13}C NMR spectrum

($CDCl_3$, 150.90 MHz, 295 K)

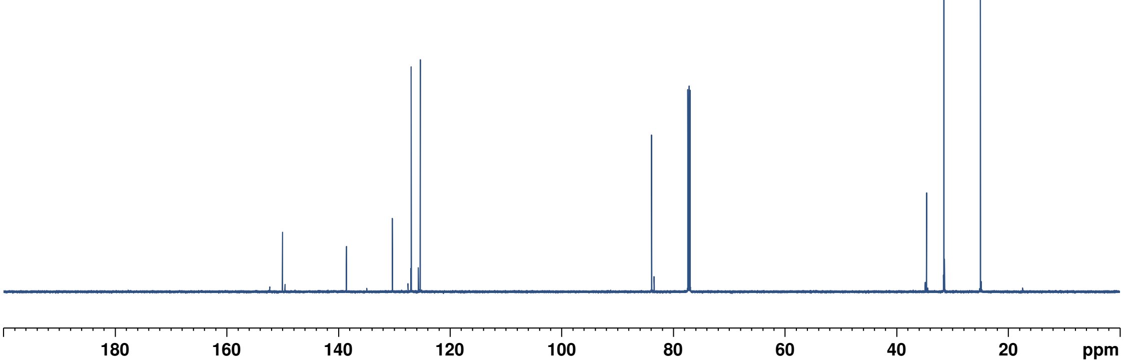

Compound α-3f

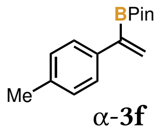

α-3f

^{1}H NMR spectrum

($CDCl_3$, 399.89 MHz, 295 K)

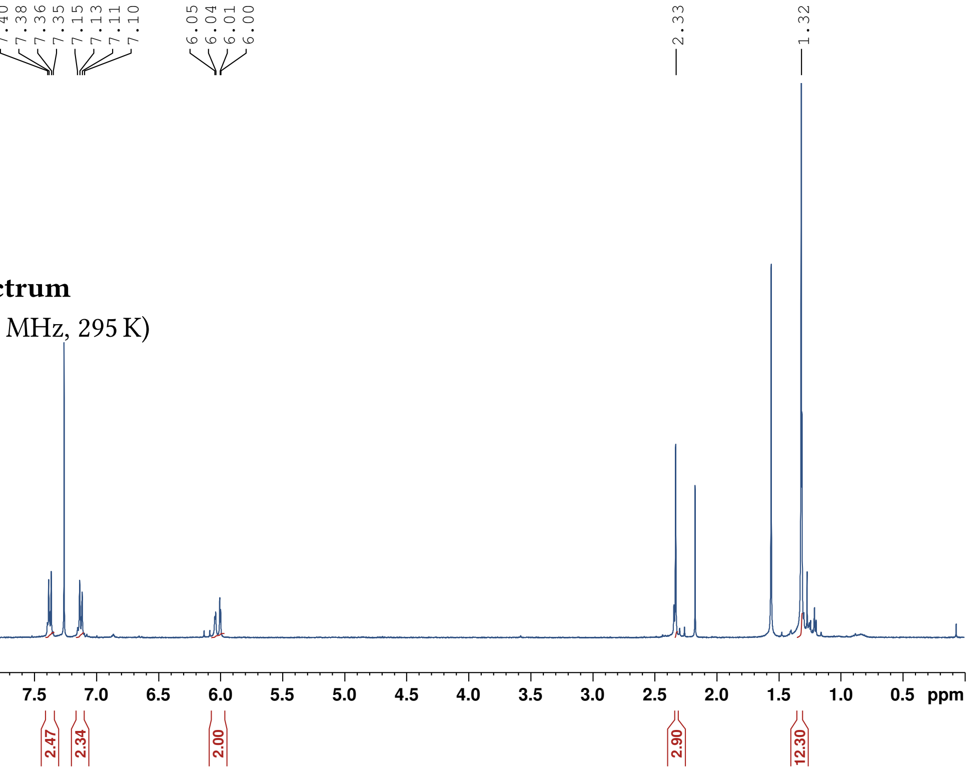

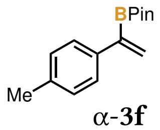

α-3f

^{13}C NMR spectrum

($CDCl_3$, 100.55 MHz, 295 K)

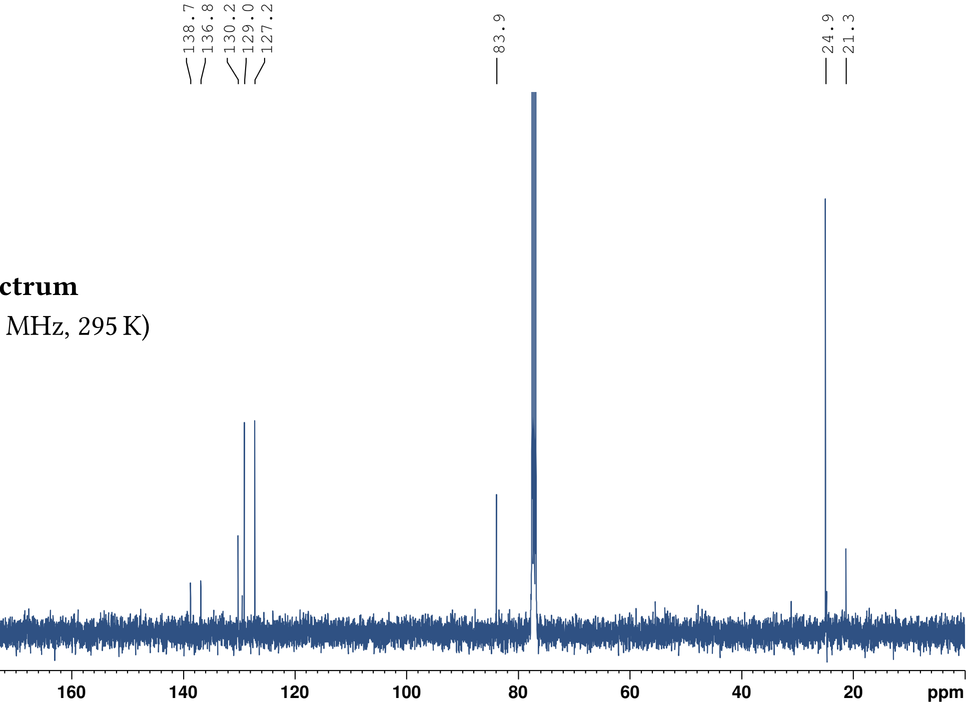

Compound α-3g

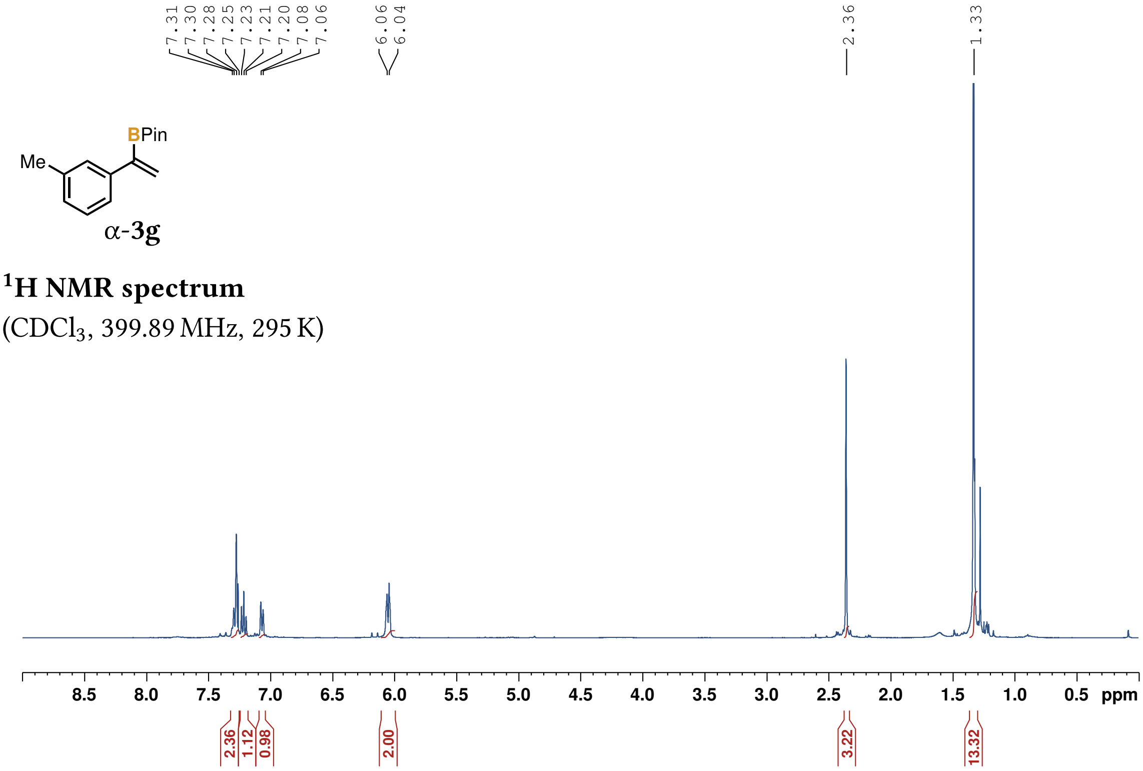

α-3g

^{1}H NMR spectrum

($CDCl_3$, 399.89 MHz, 295 K)

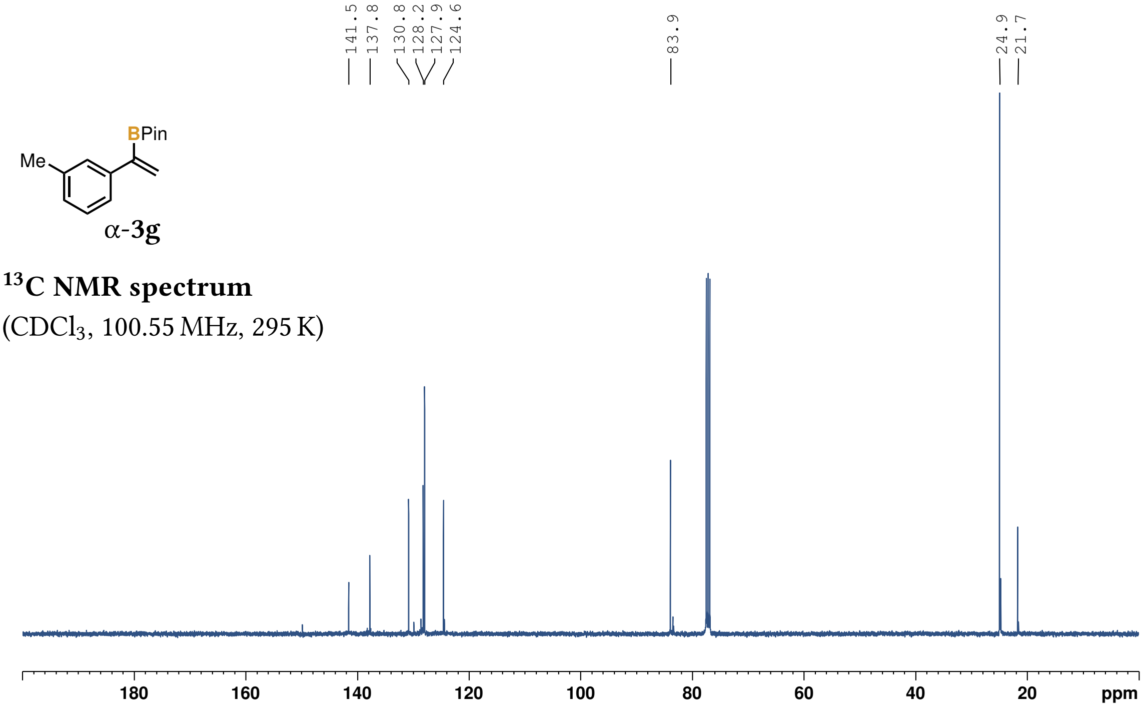

α-3g

^{13}C NMR spectrum

($CDCl_3$, 100.55 MHz, 295 K)

Compound α-3h

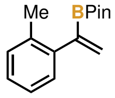

α-3h

^{1}H NMR spectrum

($CDCl_3$, 399.89 MHz, 295 K)

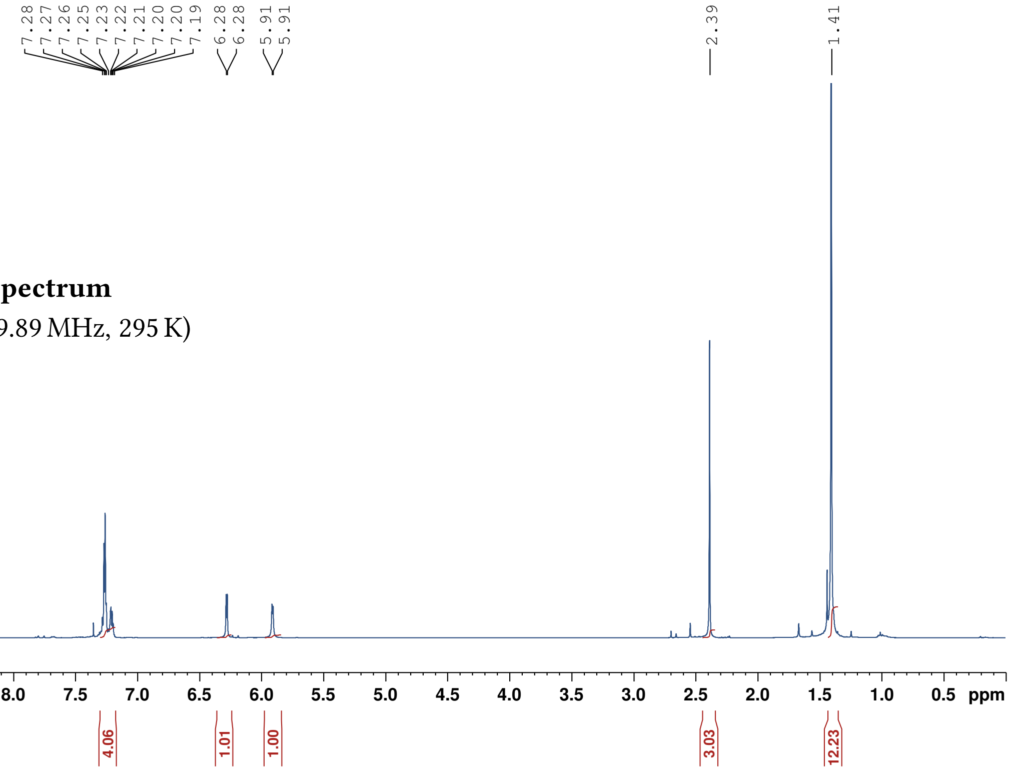

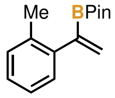

α-3h

^{13}C NMR spectrum

($CDCl_3$, 100.55 MHz, 295 K)

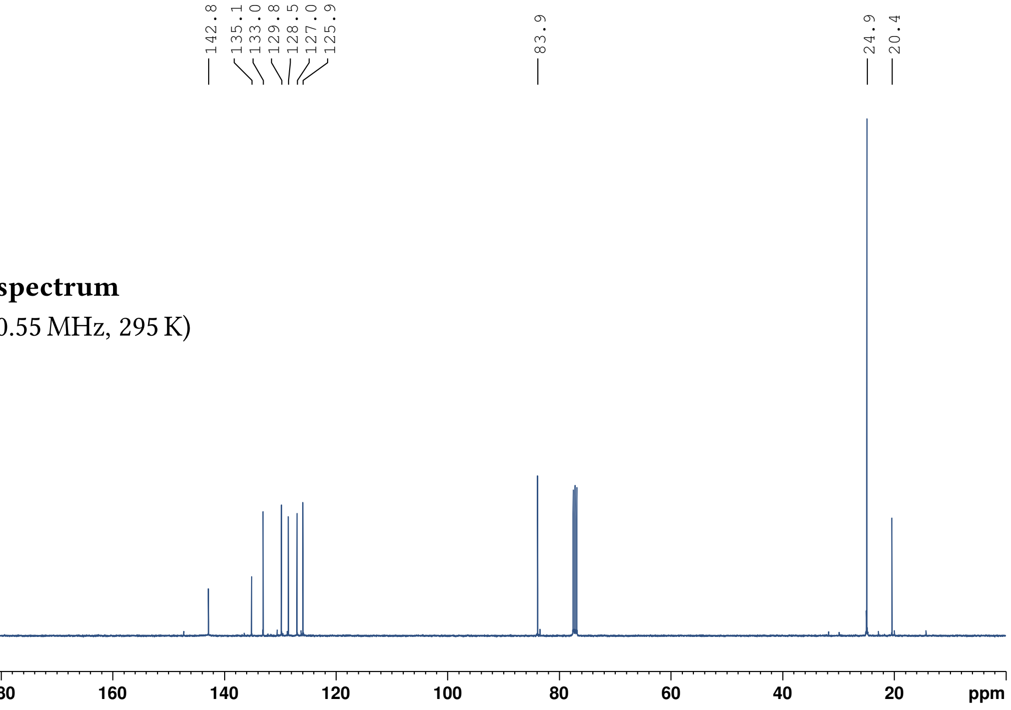

Compound α-3i

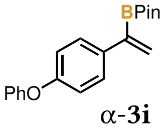

α-3i

^{1}H NMR spectrum

($CDCl_3$, 600.15 MHz, 295 K)

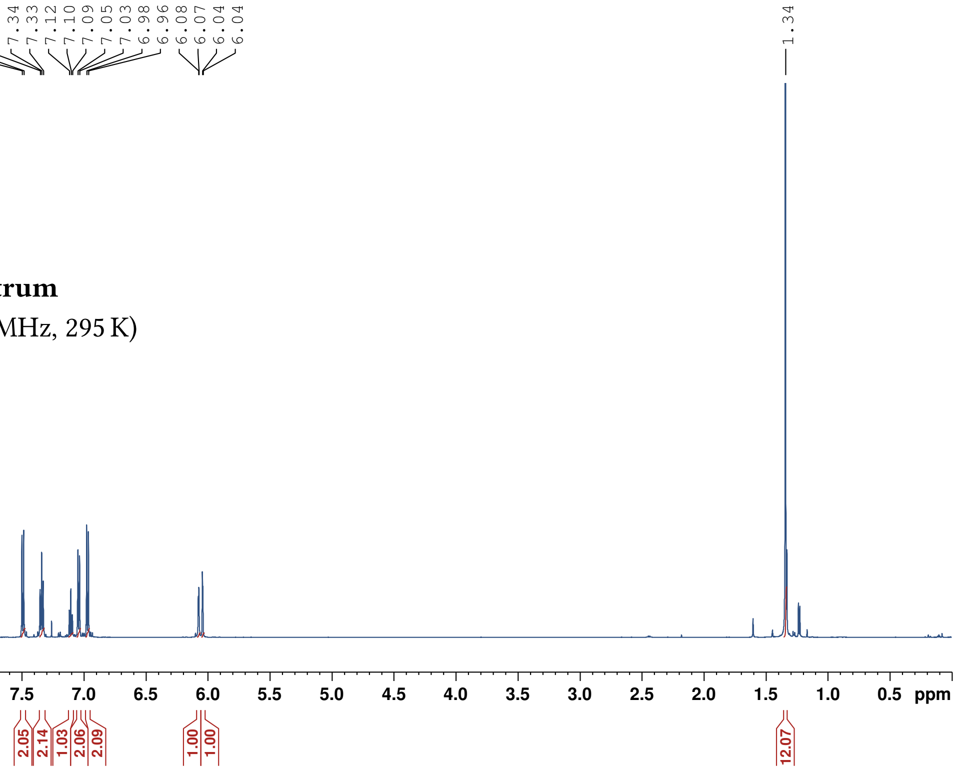

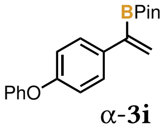

α-3i

^{13}C NMR spectrum

($CDCl_3$, 150.90 MHz, 295 K)

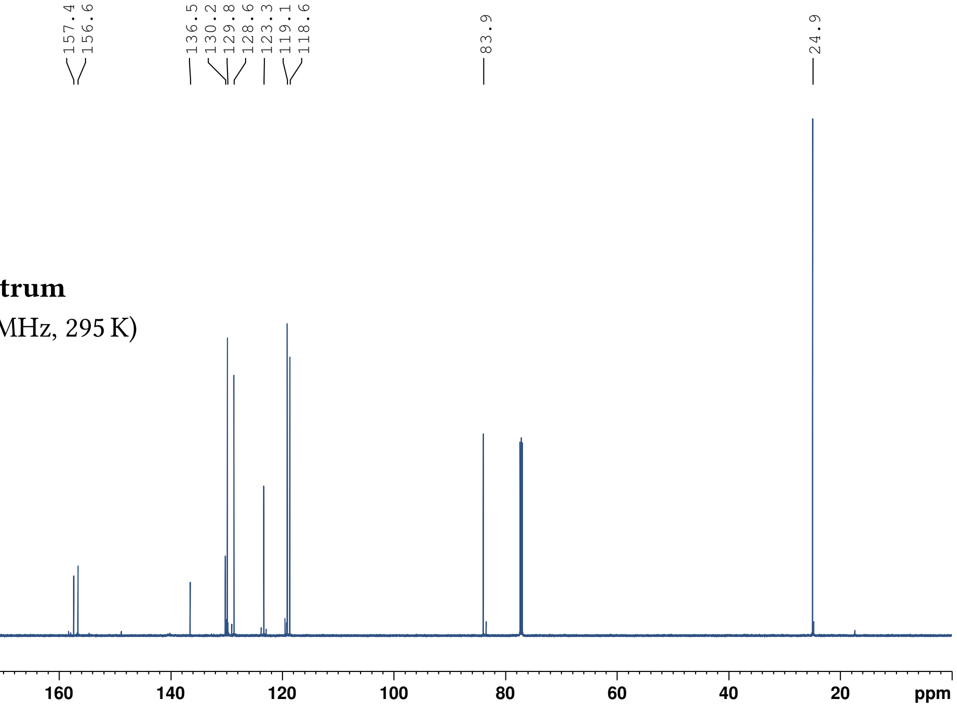

Compound α-3j

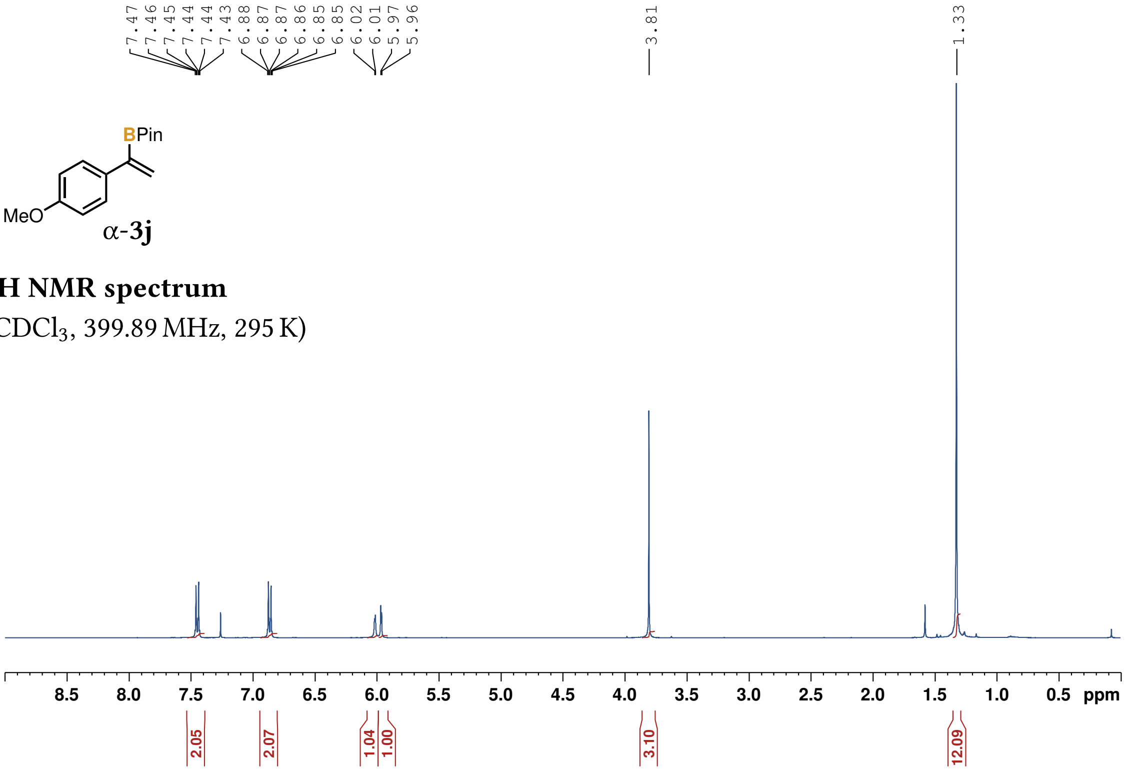

^{1}H NMR spectrum

($CDCl_3$, 399.89 MHz, 295 K)

α-3j

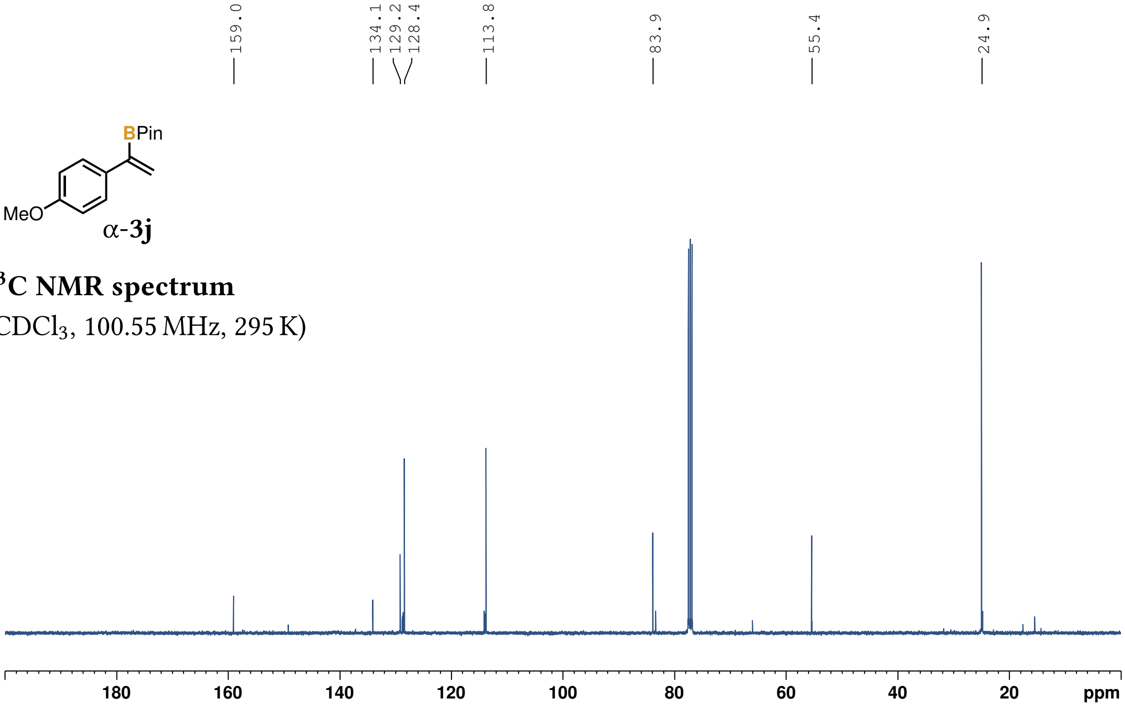

^{13}C NMR spectrum

($CDCl_3$, 100.55 MHz, 295 K)

Compound α-3k

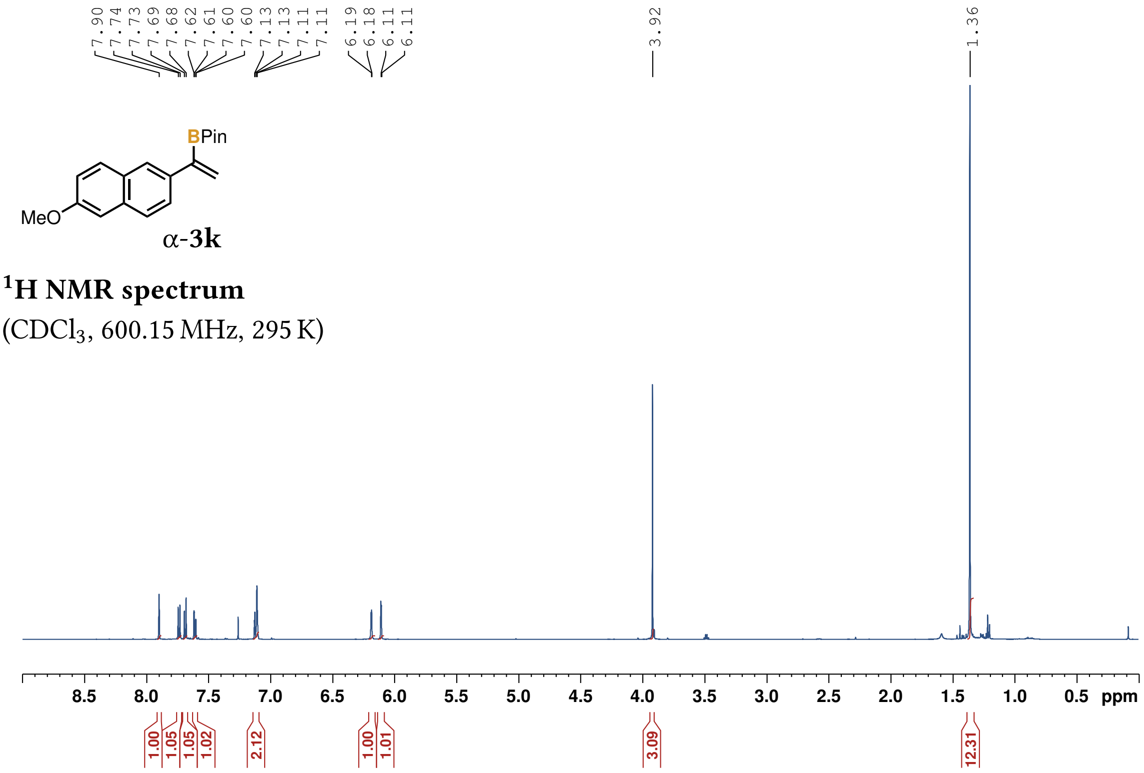

^{1}H NMR spectrum

($CDCl_3$, 600.15 MHz, 295 K)

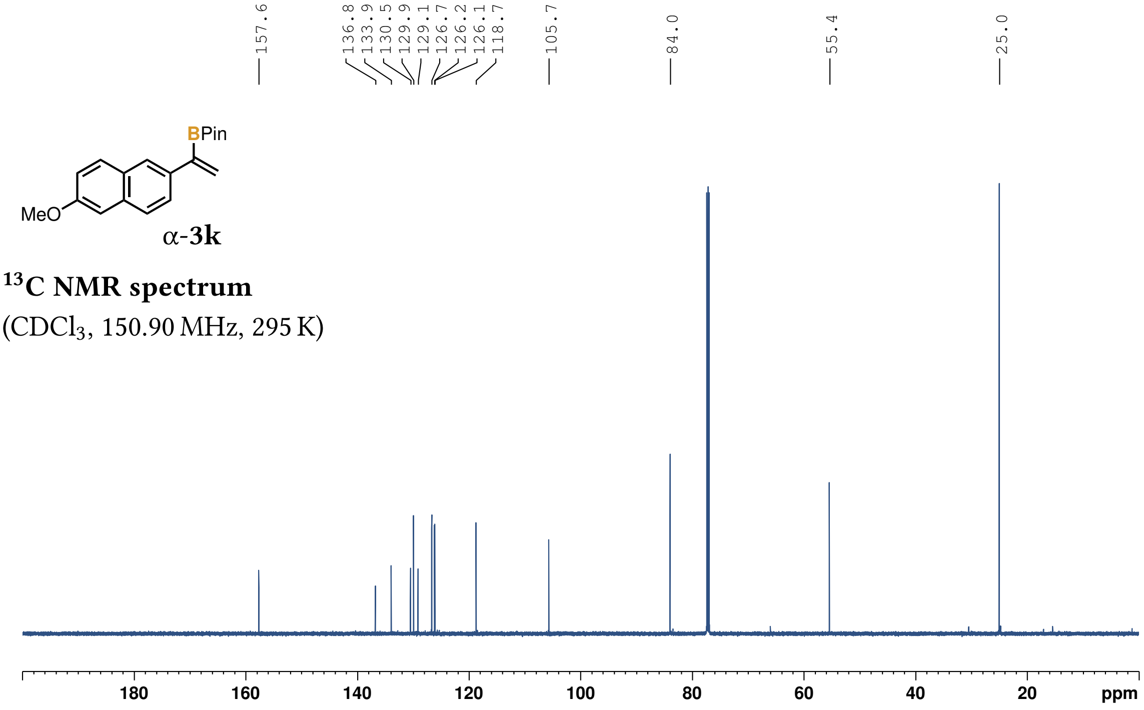

^{13}C NMR spectrum

($CDCl_3$, 150.90 MHz, 295 K)

Compound α-3l

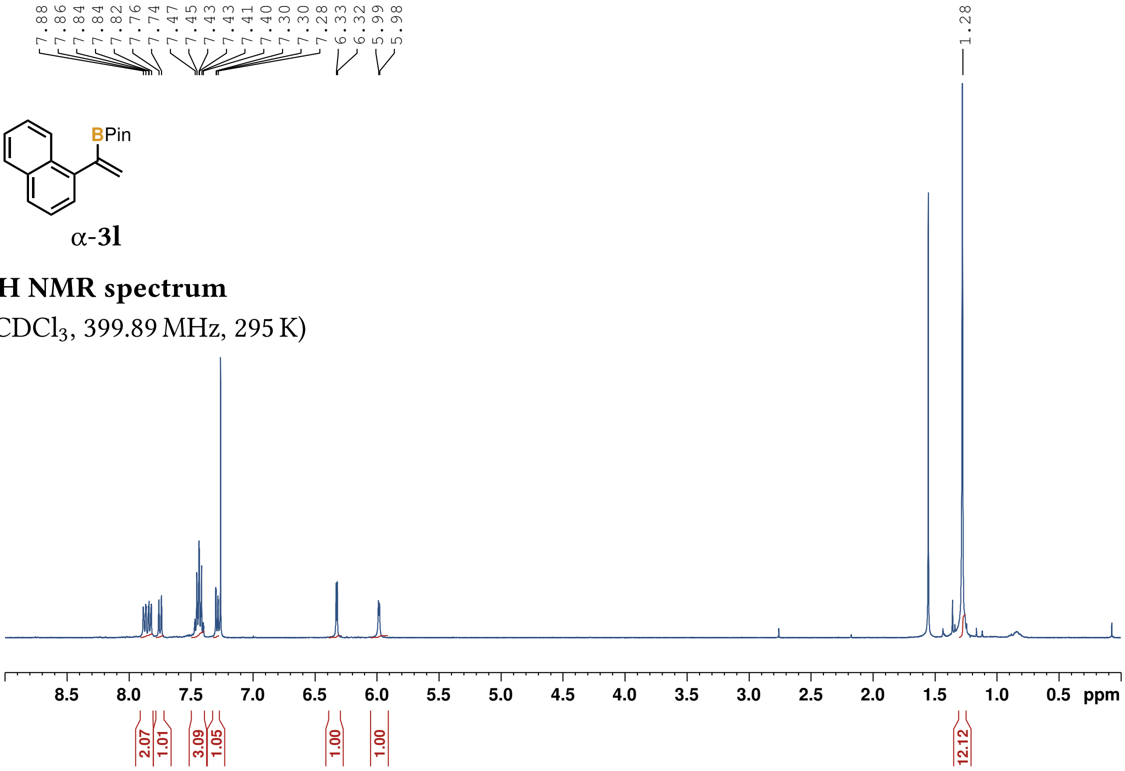

α-3l

^{1}H NMR spectrum
($CDCl_3$, 399.89 MHz, 295 K)

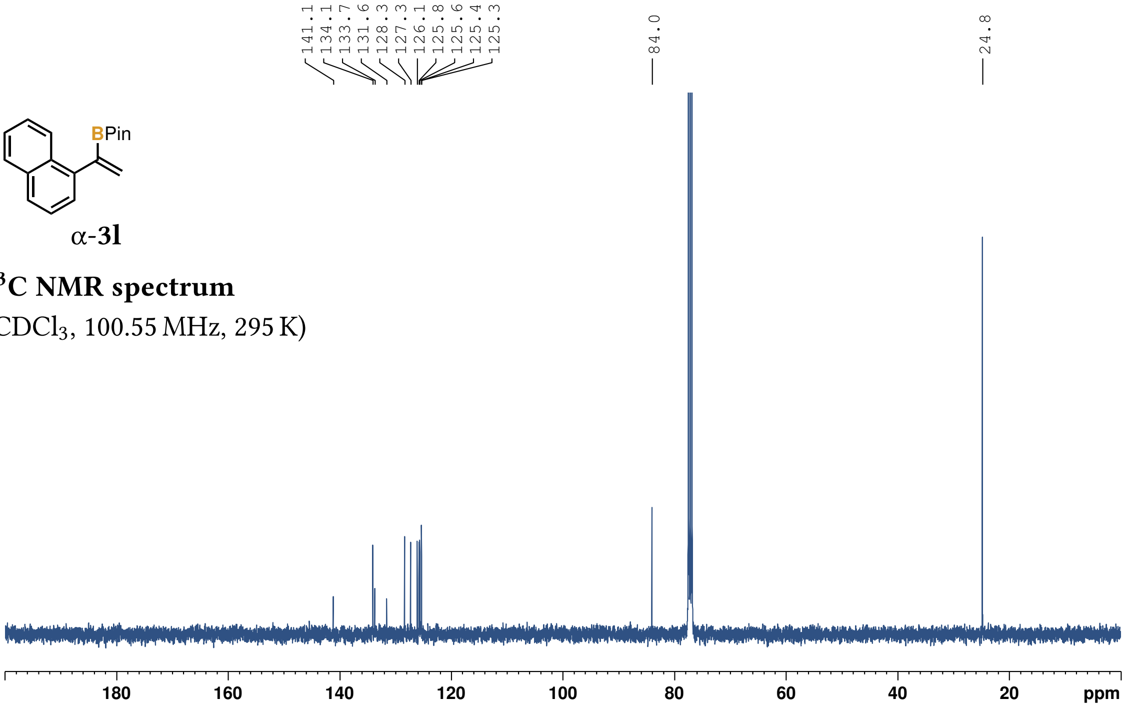

α-3l

^{13}C NMR spectrum
($CDCl_3$, 100.55 MHz, 295 K)

Compound α-3m

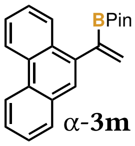

α-3m

^{1}H NMR spectrum

($CDCl_3$, 600.15 MHz, 295 K)

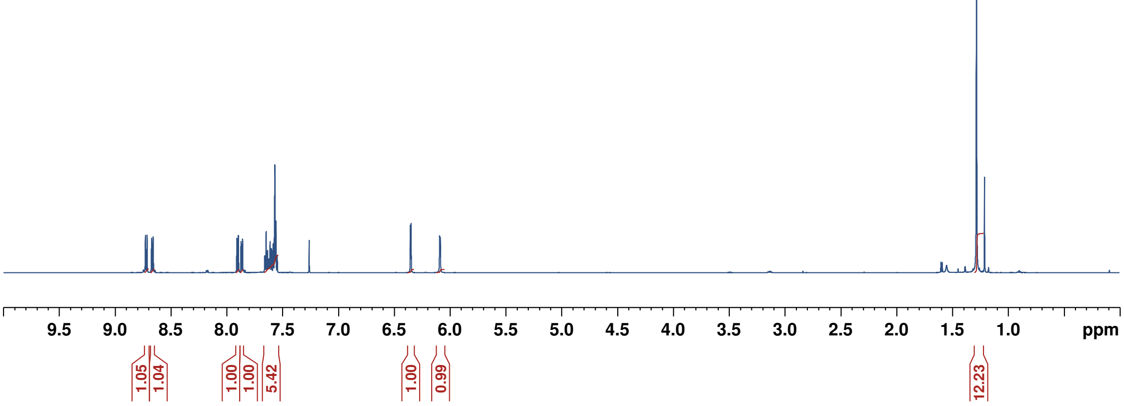

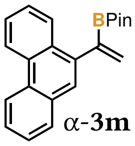

α-3m

^{13}C NMR spectrum

($CDCl_3$, 150.90 MHz, 295 K)

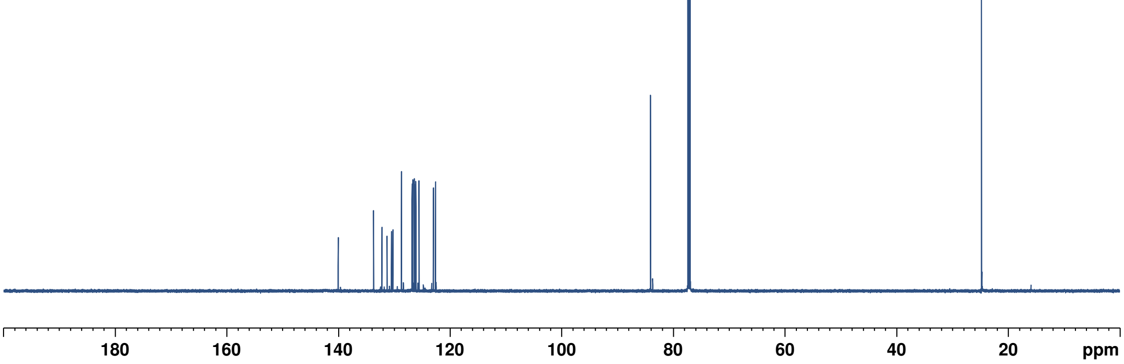

Compound α-3n

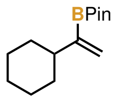

α-3n

^{1}H NMR spectrum

($CDCl_3$, 399.89 MHz, 295 K)

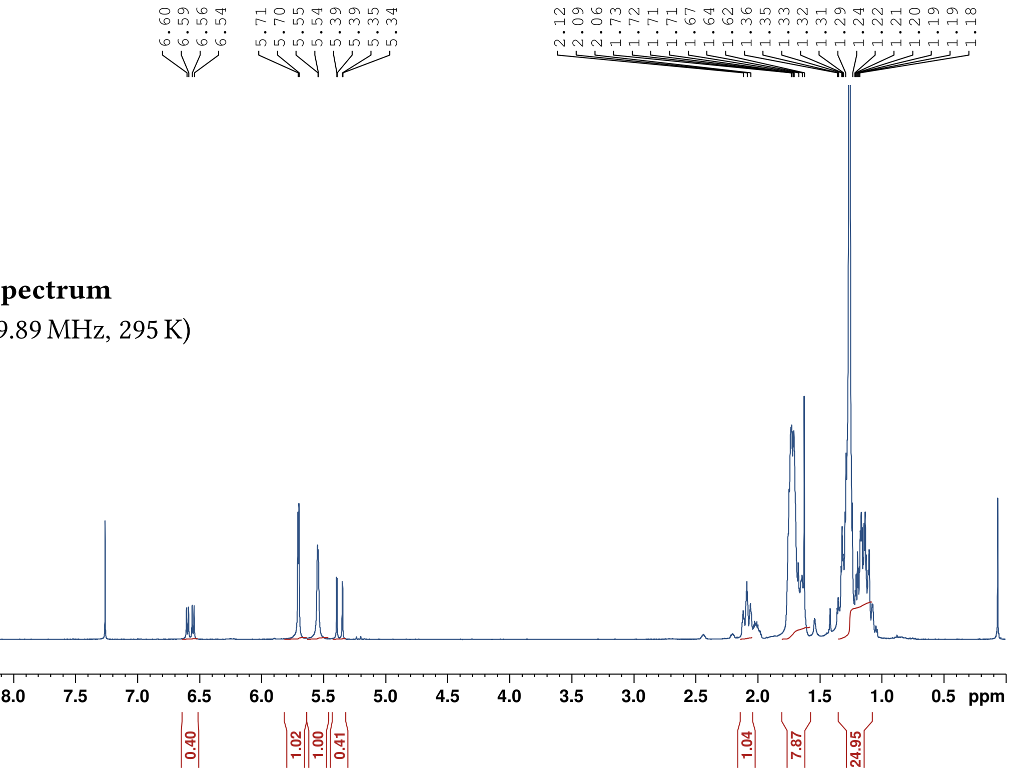

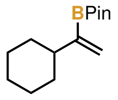

α-3n

^{13}C NMR spectrum

($CDCl_3$, 100.55 MHz, 295 K)

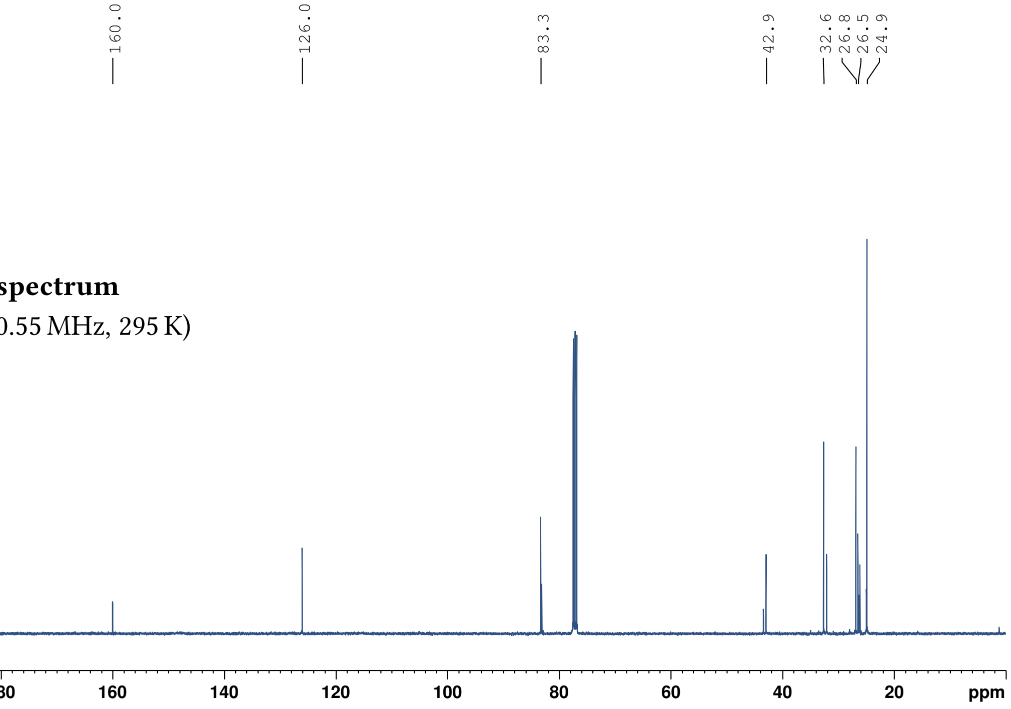

Compound α-3o

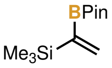

α-**3o**

^{1}H NMR spectrum

($CDCl_3$, 600.15 MHz, 295 K)

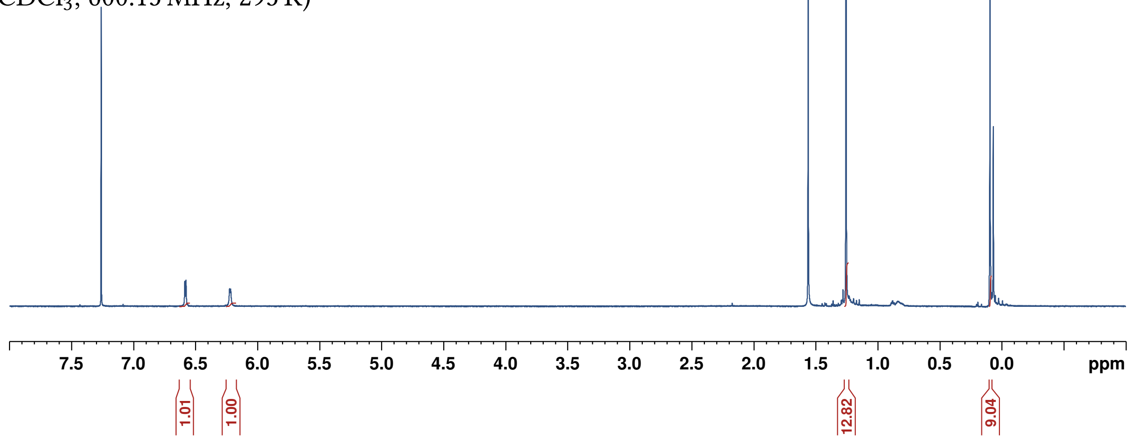

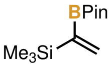

α-**3o**

^{13}C NMR spectrum

($CDCl_3$, 150.90 MHz, 295 K)

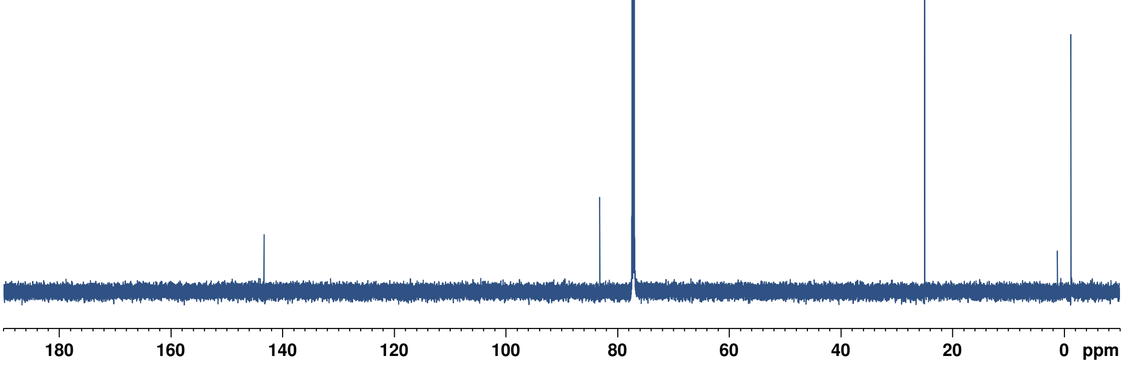

Compound α-3p

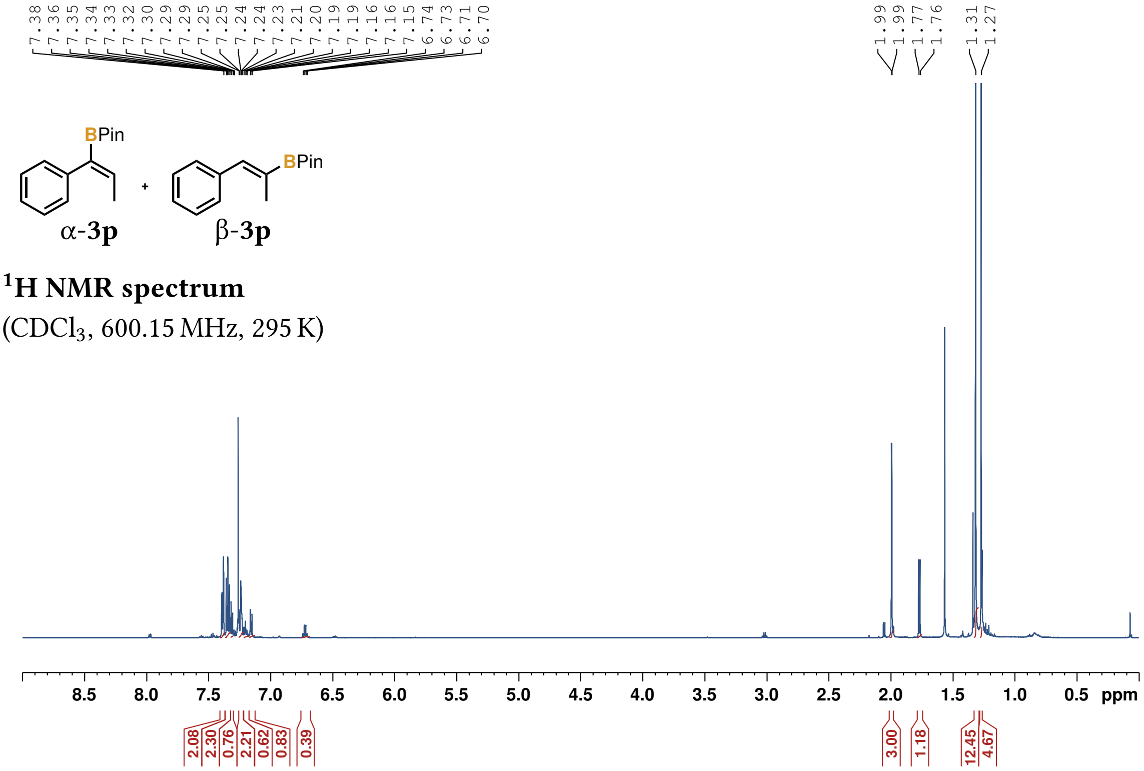

α-3p β-3p

^{1}H NMR spectrum

($CDCl_3$, 600.15 MHz, 295 K)

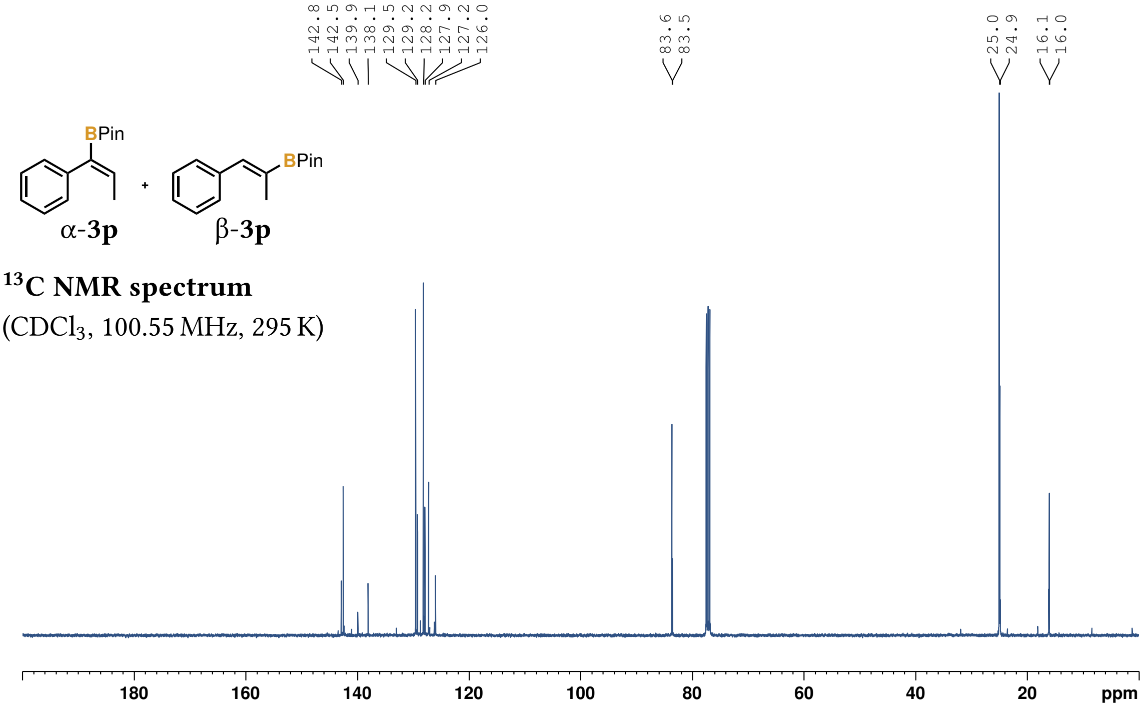

α-3p β-3p

^{13}C NMR spectrum

($CDCl_3$, 100.55 MHz, 295 K)

Compound α-3q

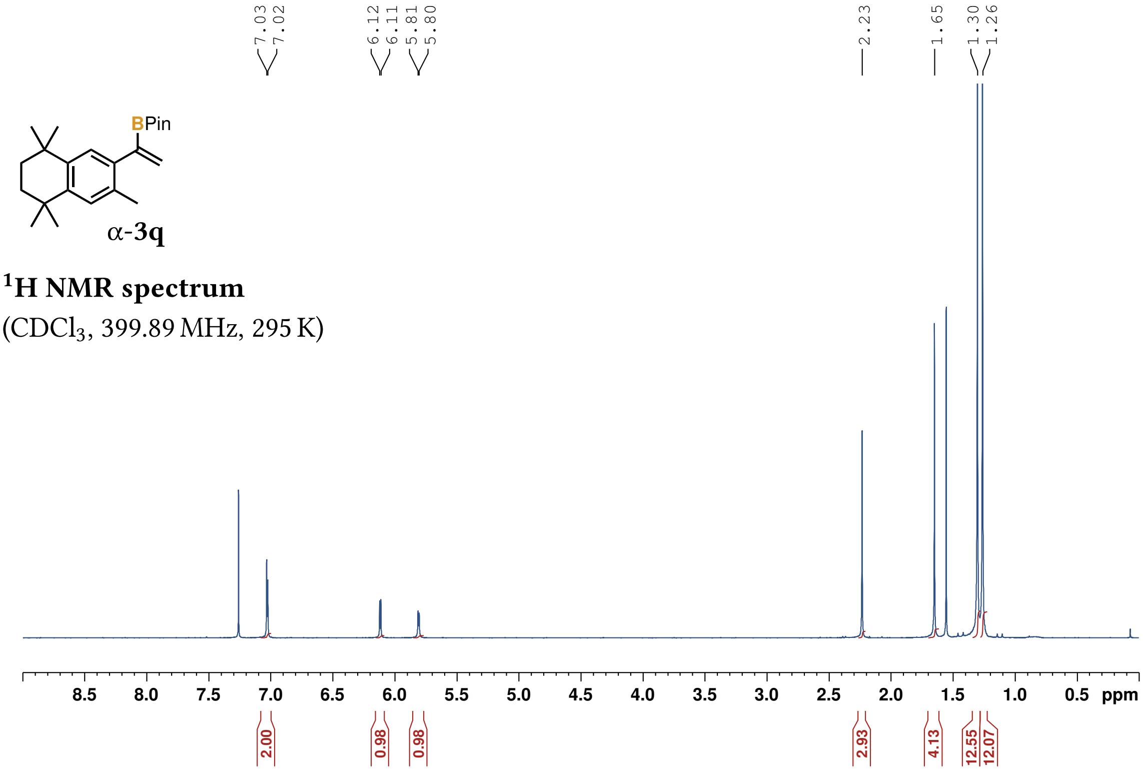

^{1}H NMR spectrum
($CDCl_3$, 399.89 MHz, 295 K)

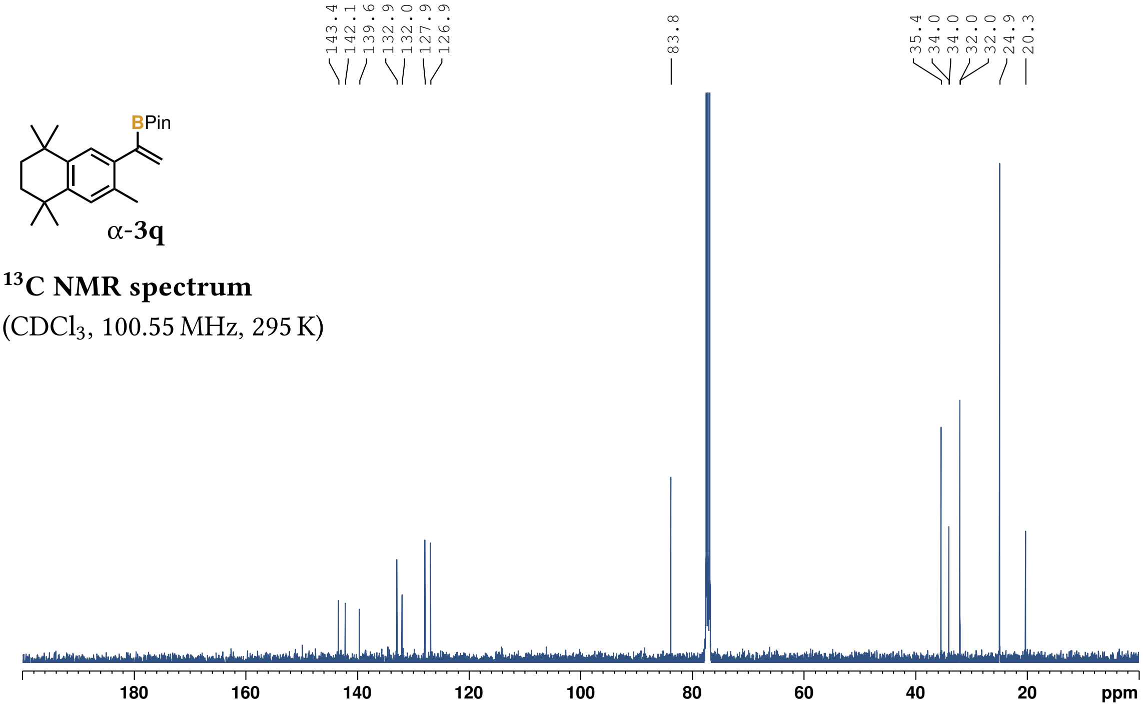

^{13}C NMR spectrum
($CDCl_3$, 100.55 MHz, 295 K)

Compound (*Z*)-*int*-**8a**

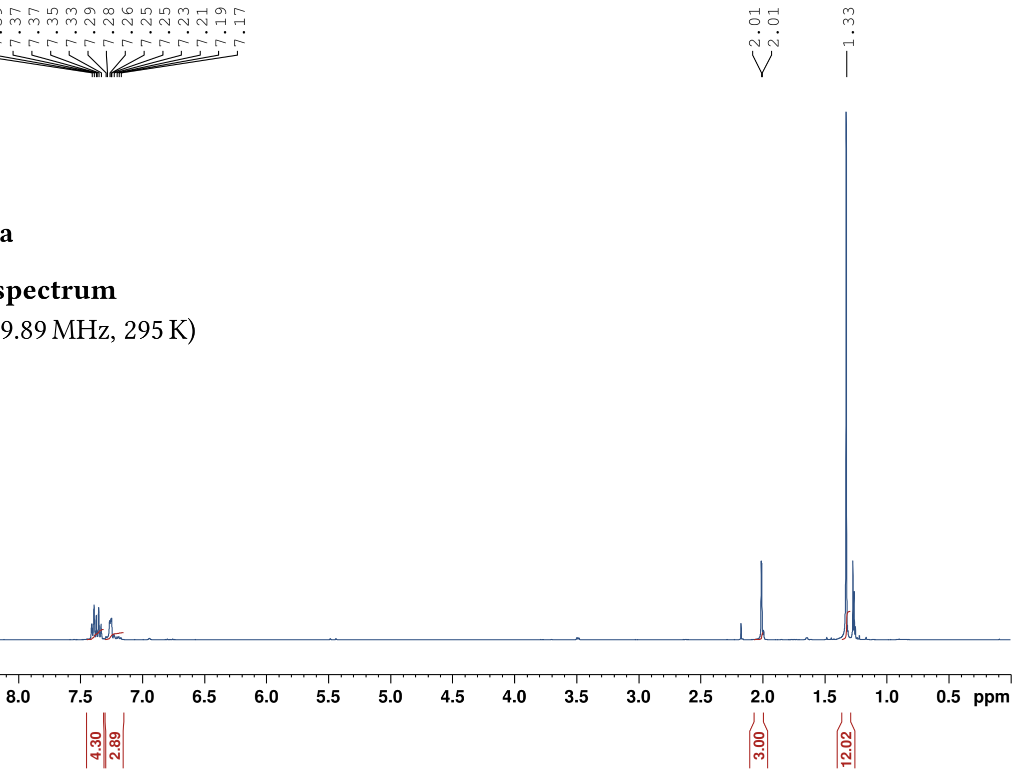

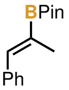

(*Z*)-*int*-**8a**

^{1}H NMR spectrum

($CDCl_3$, 399.89 MHz, 295 K)

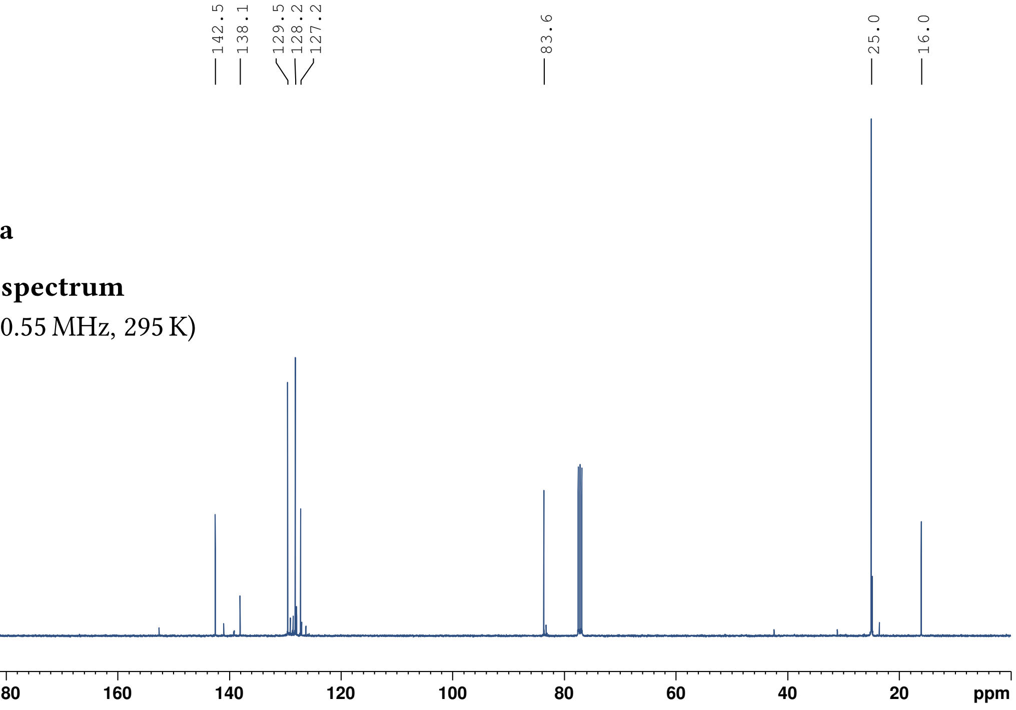

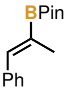

(*Z*)-*int*-**8a**

^{13}C NMR spectrum

($CDCl_3$, 100.55 MHz, 295 K)

Compound (*Z*)-*int*-8b

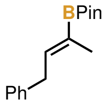

(*Z*)-*int*-**8b**

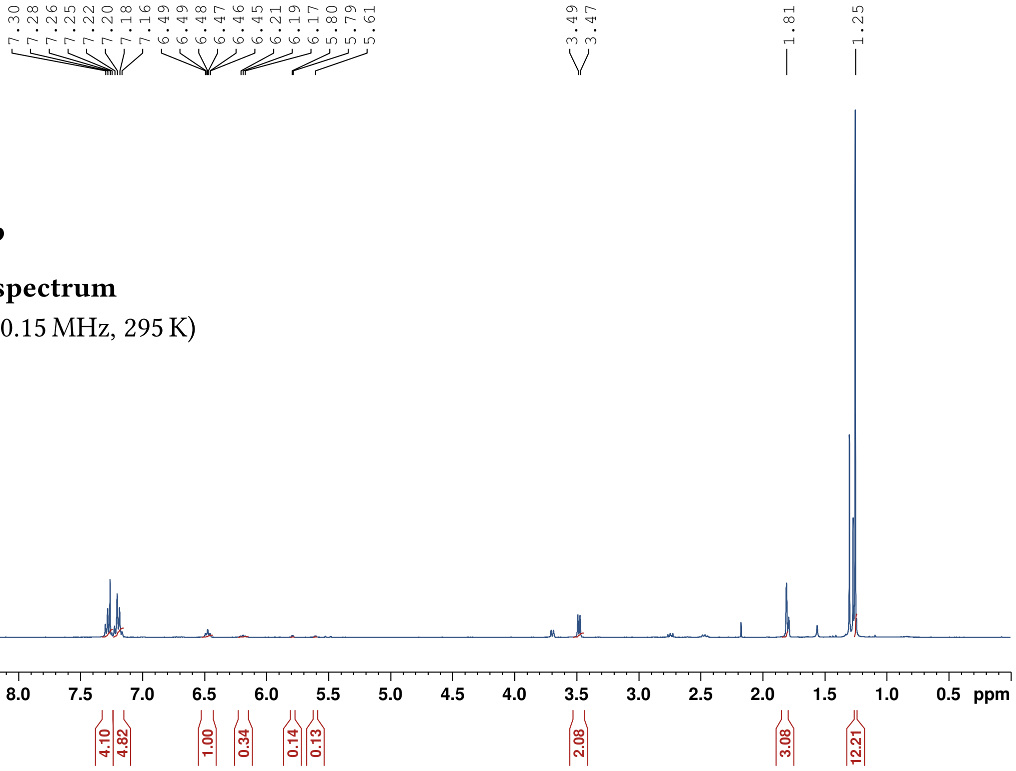

^{1}H NMR spectrum

($CDCl_3$, 600.15 MHz, 295 K)

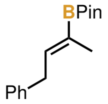

(*Z*)-*int*-**8b**

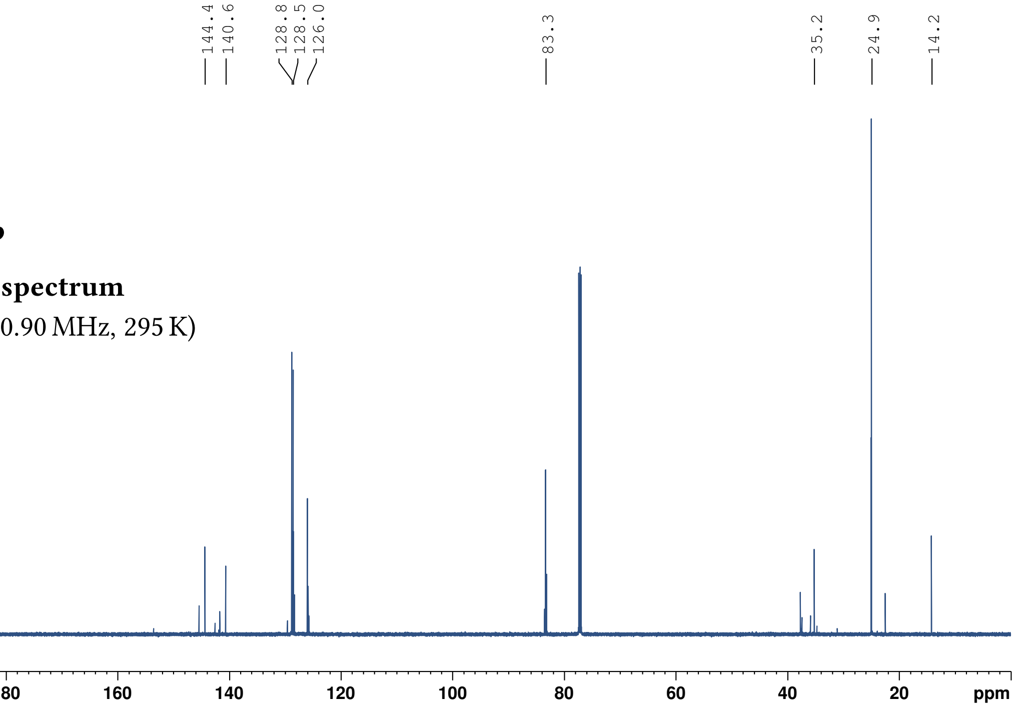

^{13}C NMR spectrum

($CDCl_3$, 150.90 MHz, 295 K)

Compound (*Z*)-*int*-8c

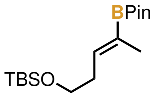

(*Z*)-*int*-**8c**

^{1}H NMR spectrum
($CDCl_3$, 600.15 MHz, 295 K)

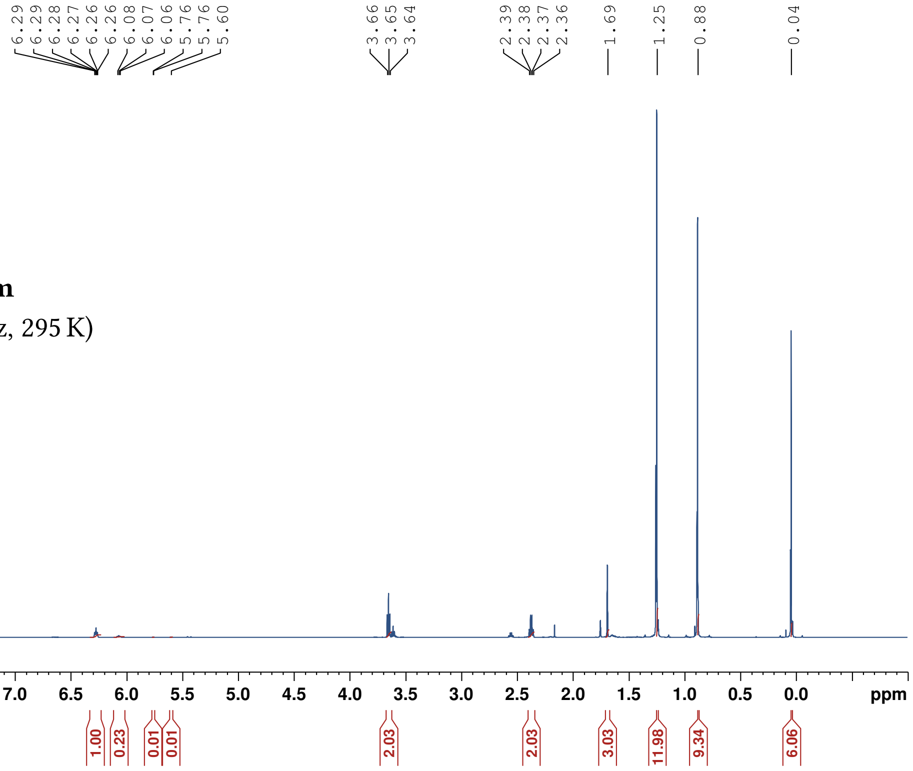

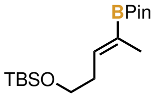

(*Z*)-*int*-**8c**

^{13}C NMR spectrum
($CDCl_3$, 150.90 MHz, 295 K)

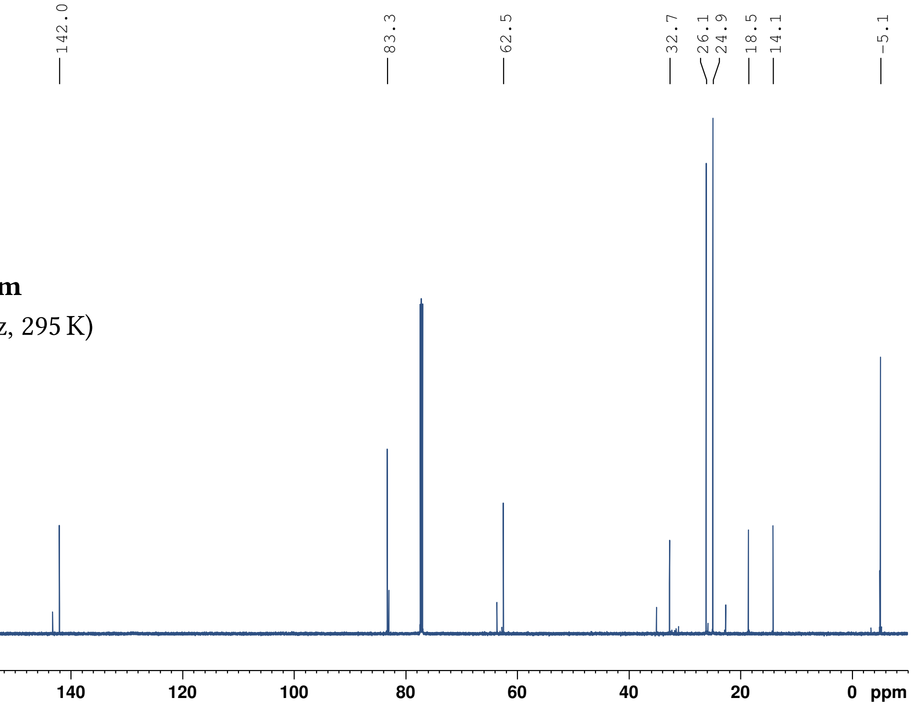

Compound (*Z*)-*int*-**8d**

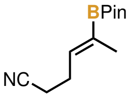

(*Z*)-*int*-**8d**

^{1}H NMR spectrum

($CDCl_3$, 600.15 MHz, 295 K)

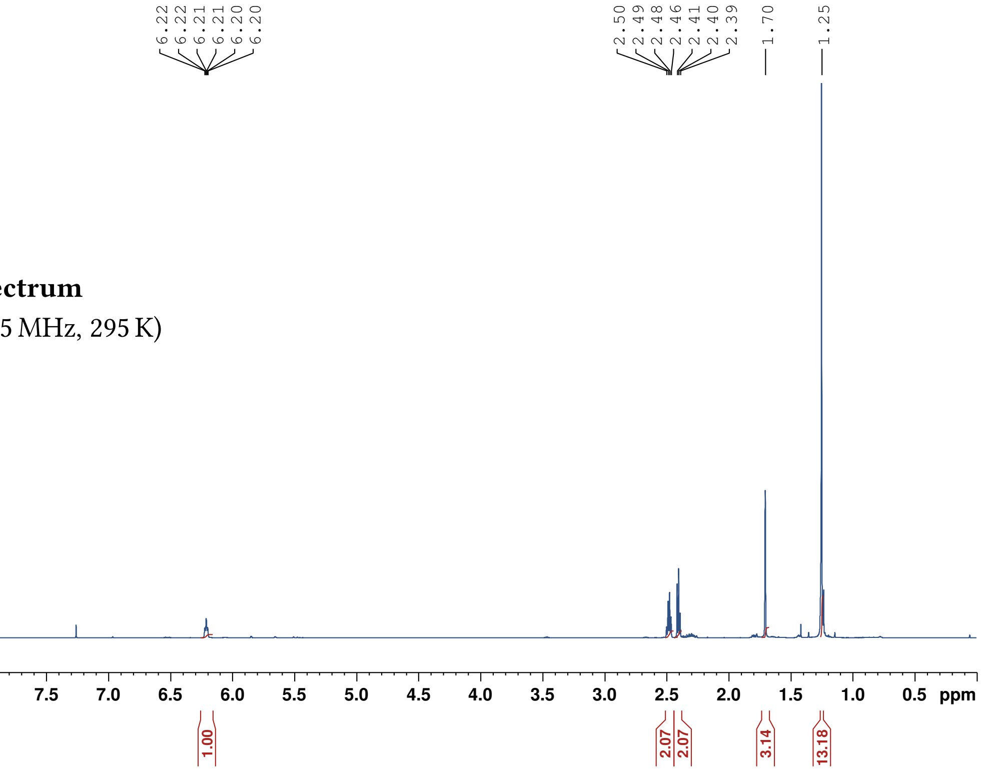

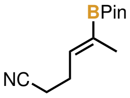

(*Z*)-*int*-**8d**

^{13}C NMR spectrum

($CDCl_3$, 150.90 MHz, 295 K)

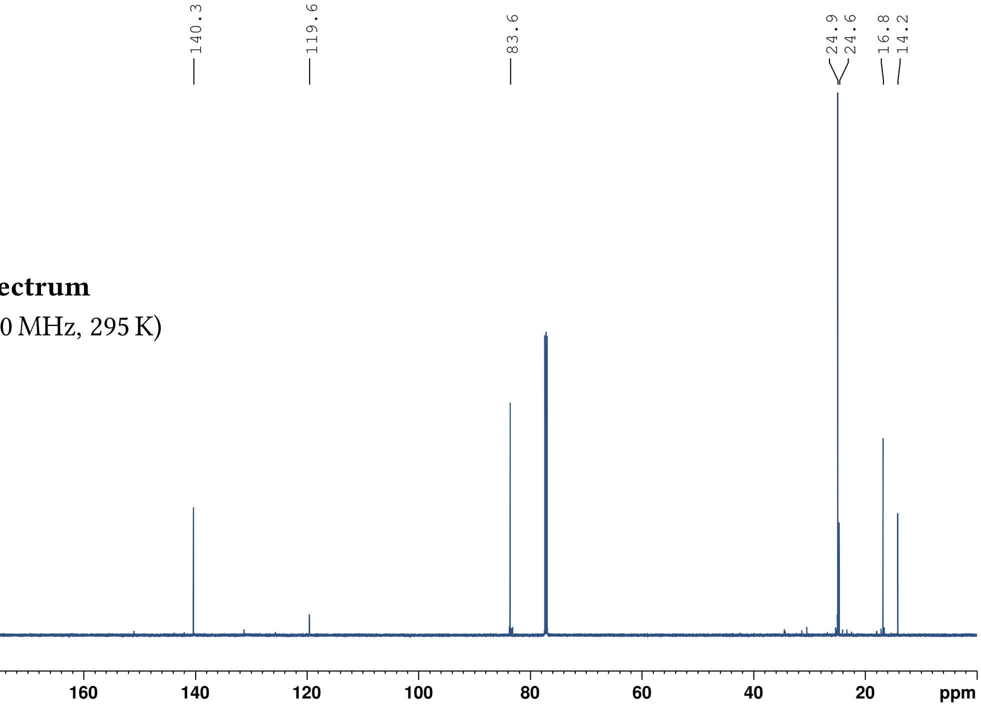

Compound (*E*)-*int*-**8b**

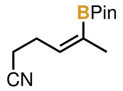

(*E*)-*int*-**8b**

^{1}H NMR spectrum
($CDCl_3$, 600.15 MHz, 295 K)

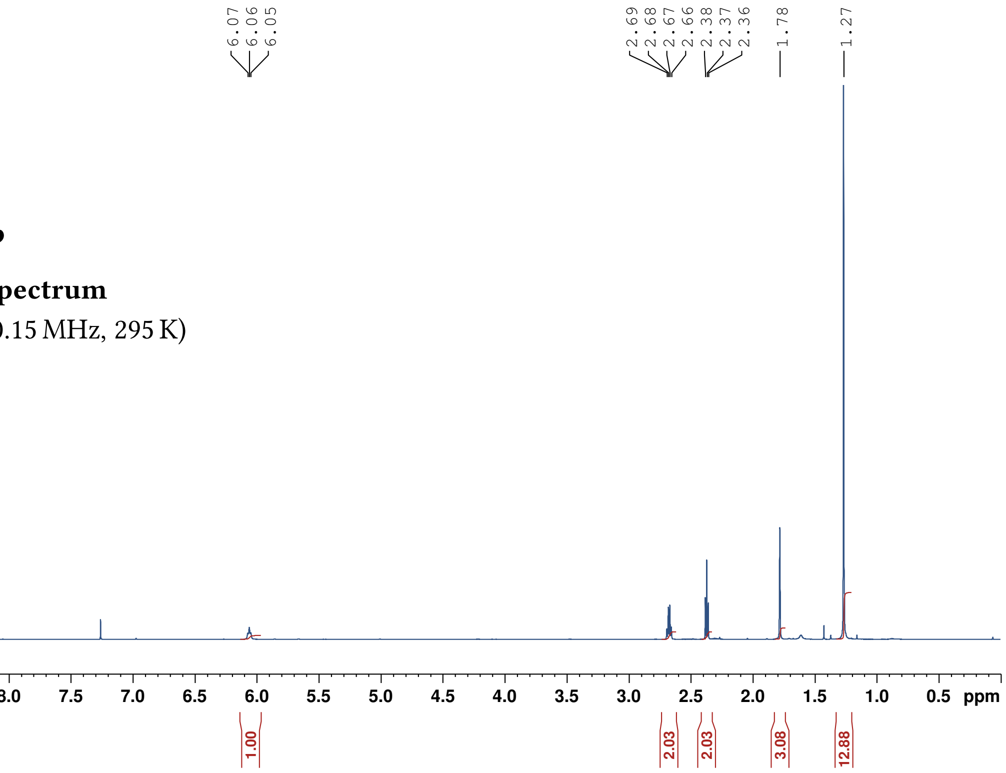

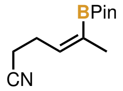

(*E*)-*int*-**8b**

^{13}C NMR spectrum
($CDCl_3$, 150.90 MHz, 295 K)

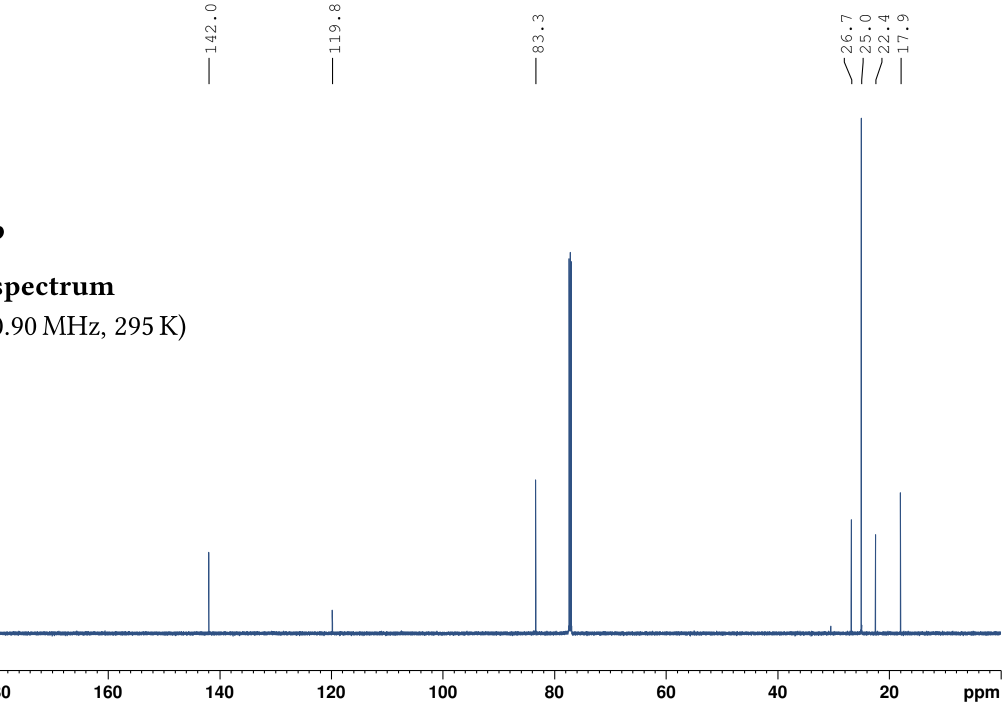

References

[1] W. L. F. Armarego, C. L. L. Chai, *Purification of laboratory chemicals*, Elsevier/Butterworth-Heinemann, Amsterdam, Boston, **2009**.

[2] H. E. Gottlieb, K. Vadim, A. Nudelman, *J. Org. Chem.* **1997**, *62*, 7512–7515.

[3] G. R. Fulmer, A. J. M. Miller, N. H. Sherden, H. E. Gottlieb, A. Nudelman, B. M. Stoltz, J. E. Bercaw, K. I. Goldberg, *Organometallics* **2010**, *29*, 2176–2179.

[4] Q.-H. Deng, H. Wadepohl, L. H. Gade, *Chem. Eur. J.* **2011**, *17*, 14922–14928.

[5] D. Zhu, F. F. B. J. Janssen, P. H. M. Budzelaar, *Organometallics* **2010**, *29*, 1897–1908.

[6] P. Alborés, L. M. Carrella, W. Clegg, P. García-Álvarez, A. R. Kennedy, J. Klett, R. E. Mulvey, E. Rentschler, L. Russo, *Angew. Chem. Int. Ed.* **2009**, *48*, 3317–3321.

[7] Y. Kitabayashi, T. Fukuyama, S. Yokoshima, *Org. Biomol. Chem.* **2018**, *16*, 3556–3559.

[8] P. Šafář, Š. Marchalín, M. Šoral, J. Moncol, A. Daïch, *Org. Lett.* **2017**, *19*, 4742–4745.

[9] P. Zhang, J. Meijide Suárez, T. Driant, E. Derat, Y. Zhang, M. Ménand, S. Roland, M. Sollogoub, *Angew. Chem. Int. Ed.* **2017**, *56*, 10821–10825.

[10] H. Jang, A. R. Zhugralin, Y. Lee, A. H. Hoveyda, *J. Am. Chem. Soc.* **2011**, *133*, 7859–7871.

[11] V. Němec, M. Hylsová, L. Maier, J. Flegel, S. Sievers, S. Ziegler, M. Schröder, B. T. Berger, A. Chaikuad, B. Valčíková, S. Uldrijan, S. Drápela, K. Souček, H. Waldmann, S. Knapp, K. Paruch, *Angew. Chem. Int. Ed.* **2019**, *58*, 1062–1066.

[12] M. D. Aparece, C. Gao, G. J. Lovinger, J. P. Morken, *Angew. Chem. Int. Ed.* **2019**, *58*, 592–595.

[13] T. Kurahashi, T. Hata, H. Masai, H. Kitagawa, M. Shimizu, T. Hiyama, *Tetrahedron* **2002**, *58*, 6381–6395.

[14] M. Magre, B. Maity, A. Falconnet, L. Cavallo, M. Rueping, *Angew. Chem. Int. Ed.* **2019**, 7025–7029.

[15] W. Guan, A. K. Michael, M. L. McIntosh, L. Koren-Selfridge, J. P. Scott, T. B. Clark, *J. Org. Chem.* **2014**, *79*, 7199–7204.

[16] M. J. Hesse, C. P. Butts, C. L. Willis, V. K. Aggarwal, *Angew. Chem. Int. Ed.* **2012**, *51*, 12444–12448.

[17] H. Yoshida, Y. Takemoto, K. Takaki, *Chem. Commun.* **2014**, *50*, 8299–8302.

[18] H. Braunschweig, F. Guethlein, L. Mailänder, T. B. Marder, *Chem. Eur. J.* **2013**, *19*, 14831–14835.

[19] G. Lesley, P. Nguyen, N. J. Taylor, T. B. Marder, A. J. Scott, W. Clegg, N. C. Norman, *Organometallics* **1996**, *15*, 5137–5154.

[20] K. Kabsch, in: M. G. Rossmann, E. Arnold (eds.), "*International Tables for Crystallography*" *Vol. F*, Ch. 11.3, Kluwer Academic Publishers, Dordrecht, The Netherlands, **2001**.

[21] *CrysAlisPro*, Agilent Technologies UK Ltd., Oxford, UK **2011–2014** and Rigaku Oxford Diffraction, Rigaku Polska Sp.z o.o., Wrocław, Poland **2015–2020**.

[22] *SCALE3 ABSPACK*, *CrysAlisPro*, Agilent Technologies UK Ltd., Oxford, UK **2011–2014** and Rigaku Oxford Diffraction, Rigaku Polska Sp.z o.o., Wrocław, Poland **2015–2020**.

[23] R. H. Blessing, *Acta Cryst.* **1995**, *A51*, 33–38.

[24] W. R. Busing, H. A. Levy, *Acta Cryst.* **1957**, *10*, 180.

[25] (a) L. Palatinus, *SUPERFLIP*, EPF Lausanne, Switzerland and Fyzikální ústav AV ČR, v. v. i., Prague, Czech Republic, **2007–2014**; (b) L. Palatinus, G. Chapuis, *J. Appl. Cryst.* **2007**, *40*, 786.

[26] (a) M. C. Burla, R. Caliandro, B. Carrozzini, G. L. Cascarano, C. Cuocci, C. Giacovazzo, M. Mallamo, A. Mazzone, G. Polidori, D. Siliqi *SIR2019*, CNR IC, Bari, Italy, **2019**; (b) M. C. Burla, R. Caliandro, B. Carrozzini, G. L. Cascarano, C. Cuocci, C. Giacovazzo, M. Mallamo, A. Mazzone, G. Polidori, *J. Appl. Cryst.* **2015**, *48*, 306.

[27] C. Giacovazzo "*Phasing in Crystallography*", IUCr and Oxford Science Publications, Oxford, UK, **2013**.

[28] (a) G. M. Sheldrick, *SHELXL-20xx*, University of Göttingen and Bruker AXS GmbH, Karlsruhe, Germany **2012–2018**; (b) W. Robinson, G. M. Sheldrick in: N. W. Isaaks, M. R. Taylor (eds.) "*Crystallographic Computing 4*", Ch. 22, IUCr and Oxford University Press, Oxford, UK, **1988**; (c) G. M. Sheldrick, *Acta Cryst.* **2008**, *A64*, 112; (d) G. M. Sheldrick, *Acta Cryst.* **2015**, *C71*, 3.

[29] (a) J. S. Rollett in: F. R. Ahmed, S. R. Hall, C. P. Huber (eds.) "*Crystallographic Computing*" p. 167, Munksgaard, Copenhagen, Denmark, **1970**; (b) D. Watkin in: N. W. Isaaks, M. R. Taylor (eds.) "*Crystallographic Computing 4*", Ch. 8, IUCr and Oxford University Press, Oxford, UK, **1988**; (c) P. Müller, R. Herbst-Irmer, A. L. Spek, T. R. Schneider, M. R. Sawaya in: P. Müller (ed.) "*Crystal Structure Refinement*", Ch. 5, Oxford University Press, Oxford, UK, **2006**; (d) D. Watkin, *J. Appl. Cryst.* **2008**, *41*, 491.

[30] A. Thorn, B. Dittrich, G. M. Sheldrick, *Acta Cryst.* **2012**, *A68*, 448.

[31] D. Ostfeld, I. A. Cohen, *J. Chem. Educ.* **1972**, *49*, 829.

[32] N. F. Chilton, R. P. Anderson, L. D. Turner, A. Soncini, K. S. Murray, *J. Comput. Chem.* **2013**, *34*, 1164–1175.
